# Multi-cohort transcriptomics and donor-aware single-cell analyses prioritize *S100A8* within an emergency-myeloid, HLA-DR-low state in sepsis

**DOI:** 10.1371/journal.pone.0359578

**Published:** 2026-10-21

**Authors:** Changlong Zhang, Qianqian Cui, Qiyun Wu, Wei Tang, Lei Zhang, Liang Chu

**Affiliations:** 1 The Second Affiliated Hospital of Bengbu Medical University, Bengbu, Anhui, China; 2 Bengbu Medical University, Bengbu, Anhui, China; 3 Anhui Provincial Key Laboratory of Infection and Immunity, Bengbu Medical University, Bengbu, Anhui, China; Abdul Wali Khan University Mardan, PAKISTAN

## Abstract

Sepsis is heterogeneous, and transcripts that distinguish sepsis from health may lack clinical specificity. We tested whether *S100A8* is reproducibly associated with sepsis and maps to a donor-aware myeloid state defined independently of *S100A8*. A discovery whole-blood cohort was analyzed on the log2 robust multi-array average scale using maximum-probe aggregation, limma, weighted gene co-expression network analysis (WGCNA), and leakage-controlled feature prioritization. The analysis identified 1,508 differentially expressed genes; 265 overlapped the strongest sepsis-positive WGCNA module. The network retained all 802 samples and 11,719 genes; the black-module eigengene correlated with sepsis status (r = 0.409; false-discovery rate 3.72 × 10 − 33), and *S100A8* belonged to this module (module membership 0.700). *S100A8* was upregulated (log2 fold change 1.87; false-discovery rate 2.15 × 10 − 171). Discrimination against clinical comparators was contrast dependent, and outcome analyses did not support a stable prognostic association. Donor-level monocyte pseudo-bulk analysis supported increased *S100A8* and decreased *HLA-DRA* but included only two control and four sepsis donors. Across single-cell datasets, *S100A8* tracked an emergency-myeloid program and inversely tracked antigen presentation. In THP-1 cells, *S100A8* knockdown attenuated lipopolysaccharide-associated reductions in *CD74* and HLA-DRα. Triptolide retained a similar NF-κB phosphorylation effect after *S100A8* knockdown, providing no evidence that this effect required *S100A8*. *S100A8* is therefore prioritized as a marker of a sepsis-associated emergency-myeloid, HLA-DR-low state, not as an established diagnostic, prognostic, direct drug-binding, or therapeutic target.

## Introduction

Sepsis is life-threatening organ dysfunction caused by a dysregulated host response to infection and remains a major cause of death worldwide [1,2]. Its biology spans simultaneous inflammatory activation, immune suppression, endothelial injury, coagulation abnormalities, and organ-specific responses [3]. This heterogeneity makes it unlikely that a transcript that distinguishes established sepsis from health will automatically provide a specific diagnostic test, a prognostic marker, or a causal therapeutic target.

Blood transcriptomic studies have identified reproducible sepsis endotypes and host-response programs, including variation in innate activation and antigen presentation [4,5]. Single-cell studies further show that these bulk signals reflect shifts in cell abundance and state [6,7], while experimental sepsis models have linked emergency myelopoiesis to expansion of immature myeloid populations [8]. *S100A8*, often acting with *S100A9* as calprotectin, is abundant in activated and immature myeloid cells and participates in inflammatory signaling [9]. Circulating calprotectin has been evaluated in infection and sepsis [10], and single-cell work has described HLA-DR-low, S100A-high monocyte states in sepsis [11,12]. Recent multi-cohort and single-cell studies further support conserved severity-associated myeloid responses and early monocyte activation across infectious states [13,14]. Human studies have also linked reduced *HLA-DRA*/HLA-DR expression to impaired innate responses, while emphasizing that this axis is not a stand-alone diagnostic test [15]. Nevertheless, these observations do not establish that *S100A8* is sepsis-specific, prognostic, causal, or directly druggable.

Accordingly, we designed the study to test whether *S100A8* is reproducibly associated with sepsis across bulk cohorts and whether it maps to a donor-aware emergency-myeloid, HLA-DR-low program defined independently of *S100A8*. Differential expression, co-expression, class-balance, clinically similar comparator, outcome, and single-cell analyses were integrated with exploratory compound/structural analyses and targeted THP-1 perturbations. Drug, docking, molecular-dynamics, CETSA, and cell-based findings were interpreted as exploratory or bounded functional evidence rather than proof of clinical efficacy or a validated therapeutic target.

## Materials and methods

### Study design and public datasets

This retrospective multi-cohort study integrated public bulk and single-cell transcriptomic datasets with experiments in the established THP-1 cell line. GSE65682 (Affymetrix Human Genome U219; 760 sepsis and 42 normal-control samples) was the discovery cohort. Prespecified healthy-control validation used GSE9692, GSE26440, GSE28750, and GSE69528. Additional cohorts were selected from the available project inventory to examine non-infectious ICU illness (GSE134347), cardiogenic shock (GSE131411), postoperative sterile inflammation (GSE28750), uninfected chronic-disease controls (GSE69528), mortality or severity (GSE272769, GSE54514, and GSE63042), and treatment response (GSE110487). Single-cell analyses used GSE167363, GSE205672, and the SCP548 collection. Each cohort was processed separately; expression matrices were not merged across platforms. Cohort composition, grouping, and analytic roles are summarized in S1 Table.

Sample labels were derived from GEO/SCP metadata and the grouping rules stored with the analysis. The experimental unit was the participant or donor whenever identifiers permitted. For GSE131411, only the first ICU sample (T1) from each eligible donor was used in the sepsis-versus-cardiogenic-shock comparison; later T2/T3 samples were not treated as independent observations. In GSE54514, baseline/day-1 observations were used for mortality and APACHE analyses, and GSE110487 treatment response was analyzed as a within-patient paired change. We report all eligible comparisons, including unfavorable results. Because the discovery and most external datasets were retrospective case-control collections, performance measures were interpreted as discrimination within the sampled contrasts rather than prospective diagnostic accuracy.

### Ethics statement

Ethics approval and informed consent were not required for this study because the human analyses used only publicly available, de-identified transcriptomic datasets and no new participants or human specimens were recruited or collected. Ethics approval and consent for the original studies were obtained and reported by the original investigators. The in vitro work used an established commercially available THP-1 cell line obtained directly from Procell (CL-0233) and involved no animals or newly collected clinical material.

### Discovery-cohort preprocessing and differential expression

Expression and phenotype data were retrieved using GEOquery [16]. GSE65682 values were retained on the log2 robust multi-array average scale. GPL13667 annotation was joined by probe identifier; empty, ambiguous, and unmapped gene symbols were removed. When several probes mapped to one gene, the maximum probe value within each sample was retained. Differential expression was performed on this log2 gene-level matrix with limma [17]. A Disease-versus-Normal contrast was fitted with lmFit and empirical-Bayes moderation. Differentially expressed genes (DEGs) were defined as Benjamini-Hochberg false-discovery rate (FDR) <0.05 and |log2 fold change| > 1.

To evaluate probe-aggregation concerns, mean, median, maximum, and highest-variance rules were compared for *S100A8*. The platform mapped *S100A8* to a single probe (11753823_a_at), so all four rules produced the same *S100A8* profile. This does not imply that aggregation choice is irrelevant for every multi-probe gene.

### WGCNA and enrichment

WGCNA was rerun using the same final log2 RMA gene-level maximum-probe matrix used for differential expression [18]. All 11,719 genes with finite, nonzero variance were retained to avoid outcome-dependent variability filtering; goodSamplesGenes identified no additional exclusions. Hierarchical clustering, principal-component analysis, and sample-connectivity z scores were used as diagnostics, and no sample was excluded. A signed network and signed topological overlap matrix were constructed with Pearson correlation. pickSoftThreshold selected power 19. One-block network construction used a minimum module size of 200, deepSplit = 2, pamRespectsDendro = FALSE, and mergeCutHeight = 0.25, with random seed 20260908. Module eigengenes were correlated with disease status coded as sepsis = 1 and normal = 0, and Benjamini-Hochberg correction was applied across all eigengene-trait tests. Module membership was the correlation between each gene and its assigned module eigengene; gene significance was its correlation with disease status. Two prespecified sensitivity analyses were performed without result-directed tuning. The top-50%-median-absolute-deviation maximum-probe network used the same rules and selected power 20; *S100A8* fell below this variability threshold and was not manually restored. A separate mean-probe gene-level network retained 11,518 finite, nonzero-variance genes and all 802 samples; because no candidate power reached signed R² > 0.90, the predefined fallback power 6 was used with the same remaining network parameters. Analysis scripts and numerical source data supporting the study, together with the RDS reassembly script and SHA-256 checksum manifest, are provided in S2 File. The complete maximum-probe WGCNA RDS object is supplied as four lossless binary parts in S3 File, S4 File, S5 File, and S6 File. The corresponding mean-probe sensitivity-analysis RDS object is supplied as four lossless binary parts in S7 File, S8 File, S9 File, and S10 File.

The most strongly sepsis-positive module was intersected with the corrected DEG set. Gene Ontology (GO) and KEGG enrichment was performed with clusterProfiler and org.Hs.e.g.,db [19], using all mapped genes in the 11,719-gene network as the background, a minimum gene-set size of 10, and Benjamini-Hochberg correction. All tested module-trait and enrichment results were retained; GO or KEGG terms with FDR < 0.05 were considered significant.

### Feature prioritization, class imbalance, and leakage control

Machine learning was used to prioritize features, not to construct a deployable clinical model. Inputs were the log2 gene-level expression values. LASSO, component-wise gradient boosting (glmBoost), gradient-boosted trees (XGBoost), and Ranger random forests were evaluated [20–23]. In the main stability analysis, all preprocessing, variance filtering, scaling, feature screening, hyperparameter tuning, and balancing were performed within training folds. Stratified five-fold outer cross-validation was repeated 20 times, yielding 100 outer validation folds. Hyperparameters were selected only within each outer-training set by three-fold inner cross-validation using ROC AUC; outer folds were used once for validation. Class-weighted models, unweighted models, and random downsampling restricted to each training fold were compared. Prespecified random seeds are supplied with the code.

Complementary analyses comprised 1,000 repeated 1:1 samples retaining all 42 controls and randomly selecting 42 sepsis samples for the *S100A8* distribution, 200 repeated balanced feature-selection runs, and 1,000 class-stratified bootstrap resamples. We summarized ROC area under the curve (AUC), precision-recall AUC, balanced accuracy, sensitivity, specificity, Matthews correlation coefficient, F1 score, Brier score, calibration slope, feature rank, and selection frequency. SHAP values were used only to describe fitted-model feature contributions [24]. The full-cohort diagnostic plots in S1 Fig are descriptive and are not external validation. Performance and feature-stability summaries are provided in S5 Table.

### Clinical-comparator, decision-curve, timing, and outcome analyses

For each external cohort, *S100A8* probes were identified from the platform annotation and summarized using the prespecified rule. ROC analyses used group levels fixed as comparator followed by sepsis and direction ‘ < ,’ so higher *S100A8* was consistently oriented toward sepsis rather than selected automatically by pROC [25]. ROC AUCs and bootstrap 95% confidence intervals were calculated with pROC, and precision-recall curves with precrec [26]. Cohort-specific exploratory Youden thresholds were used for threshold-dependent measures; they were not treated as universal clinical cutoffs. Decision-curve analysis compared the *S100A8* model with treat-all and treat-none strategies over the displayed threshold range. Because these were case-control samples with study-defined case fractions, calibration intercepts, Brier scores, precision-recall AUCs, and decision curves are conditional on the sampled prevalence and should not be interpreted as estimates of prospective clinical utility. Traditional biomarkers such as C-reactive protein, procalcitonin, interleukin-6, and lactate were not consistently available and were not imputed. Direction-explicit distributions, likelihood ratios, calibration, and decision-curve status are provided in S4 Table.

Mortality and binary severity outcomes were modeled using *S100A8* standardized within cohort. Comparable mortality effects were pooled using a random-effects model. APACHE score and treatment-response associations were evaluated where metadata permitted. GSE65682 metadata were audited for sampling stage, ICU-acquired infection-event labels, title-derived suspected repeated subjects, and MARS endotype. Samples explicitly annotated as infection-event samples were removed in a sensitivity analysis. Title suffixes were used only to flag possible repeated subjects, not as definitive identifiers.

### Single-cell preprocessing and donor-level inference

GSE167363 was processed with Seurat [27]. Cells with 200−6,000 detected genes, mitochondrial fraction <20%, and erythrocyte-gene fraction <1% were retained; predicted doublets were removed using DoubletFinder [28]. SCTransform normalization [29], principal-component analysis, Harmony integration by donor [30], graph clustering, and UMAP were used for visualization and cell-class annotation. T cells, B cells, NK cells, and monocytes were identified from canonical markers.

For primary inference, raw monocyte counts were summed by donor. This donor-level unit was chosen because cells from the same individual are not statistically independent and treating them as independent observations can inflate inferential certainty [31]. Donors with at least 20 monocytes were analyzed with edgeR robust quasi-likelihood models after low-expression filtering and TMM normalization [32,33]. This threshold yielded two control and four sepsis donors; a prespecified 50-cell sensitivity threshold yielded only three donors and was considered underpowered. Cell-level mixed models were used only as sensitivity analyses. Donor-level transcription-factor activity was inferred with decoupleR/DoRothEA [34,35] and FDR corrected. Donor counts, threshold feasibility, focus-gene results, and TF-activity results are provided in S6 Table.

### Unsupervised monocyte states and non-circular signatures

Monocytes were reclustered without using *S100A8* as a grouping variable. Candidate resolutions were evaluated for donor coverage, donor dominance, cluster size, stability, and multi-gene coherence. States were annotated from inflammatory, emergency-myeloid/immature-neutrophil-like, antigen-presentation, and HLA-DR programs. The main independent state signature comprised 30 donor-stable marker genes and excluded *S100A8*, mitochondrial, ribosomal, and hemoglobin genes. A stricter sensitivity signature excluded both *S100A8* and *S100A9*. Leave-one-donor-out analyses were described as internal robustness rather than independent validation.

The independent programs were projected into GSE205672 and SCP548 and compared with *S100A8* at donor level. Antigen-presentation and NF-κB-related scores were computed from genes fixed before testing. Associations were summarized by Spearman correlation. These analyses describe program concordance and do not estimate absolute whole-blood cell proportions or establish causality.

### Drug repositioning and matched molecular docking

L1000CDS2 reverse-signature searching [36] was applied to a query formed from prioritized upregulated genes and the most strongly downregulated genes. Sixteen-hydroxytriptolide was among the computationally prioritized compounds. Triptolide, the compound available for downstream experiments, was treated as a distinct molecule. Structural similarity between PubChem-defined triptolide (CID 107985) and 16-hydroxytriptolide (CID 126556) was measured using chiral Morgan and MACCS fingerprints and maximum-common-substructure coverage.

The calcium- and zinc-bound human S100A8 structure (PDB 5HLO) was obtained from the Protein Data Bank [37]. The A/C and B/D homodimers were analyzed independently. Waters and buffer additives were removed; native calcium and zinc ions were retained for docking. Structures were protonated at pH 7.4 with PDBFixer/OpenMM [38] and prepared with Meeko 0.7.1. Twenty stereochemically defined conformers of each ligand were generated with ETKDGv3, minimized with MMFF94s, and the lowest-energy conformer was used.

AutoDock Vina 1.2.7 [39] first searched overlapping 28 × 28 × 28 Å boxes covering each dimer surface (exhaustiveness 32). The shared interface region was then evaluated in 24 × 24 × 24 Å boxes using Vina and Vinardo scoring, exhaustiveness 64, 20 output modes, and 10 predefined seeds per receptor and scoring function. Contact residues used a 4.5 Å heavy-atom cutoff. Scores from Vina and Vinardo were not compared directly. Docking evaluated predicted site compatibility; it did not establish binding affinity or direct binding.

### Molecular-dynamics simulations

Matched S100A8-triptolide complex and apo systems were rebuilt from the 5HLO-based representative pose and simulated with GROMACS 2023.2 [40]. Zinc and crystallographic additives were removed and structural calcium ions retained. The protein and water were modeled using AMBER99SB-ILDN and TIP3P [41]; triptolide was modeled using OpenFF Sage 2.2.1 with AM1-BCC ELF10 charges [42]. Systems were solvated in dodecahedral boxes with 0.15 mol/L NaCl, minimized, and equilibrated at 300 K and 1 bar. Production used a 2-fs time step for 100 ns in three independently seeded replicates per condition. The apo NPT equilibration was prospectively extended to 5 ns after its initial pressure QC hold.

Accepted trajectories underwent protein clustering across periodic boundaries followed by no-jump correction. The primary equilibrium window was 20–100 ns. Replicate-level summaries were calculated for backbone RMSD, chain distance, radius of gyration, solvent-accessible surface area, hydrogen bonds, ligand displacement from the original pocket, minimum protein-ligand distance, contacts, residue occupancy, and pooled-basis PCA/free-energy landscapes. The independent unit was the trajectory replicate (n = 3 per condition); trajectory frames were not treated as independent observations.

### THP-1 culture, treatments, RT-qPCR, and immunoblotting

THP-1 cells (Procell, CL-0233) were cultured in RPMI-1640 with 10% fetal bovine serum, 100 U/mL penicillin, and 100 μg/mL streptomycin at 37°C and 5% CO2. The medium was replaced every 2−3 days and cells were passaged at a 1:3 ratio every 5−6 days. Cells were confirmed mycoplasma negative before experiments. Triptolide (MedChemExpress, HY-32735) was prepared as a DMSO stock and diluted immediately before use; all treatment groups had the same final DMSO concentration. Lipopolysaccharide from Escherichia coli O55:B5 (Sigma-Aldrich, 93572-42-0) was used. The CCK-8 screen used 5 × 10^3 cells per well in 96-well plates, three technical replicate wells per condition, and medium-only blank wells. Cells were exposed for 24 h to LPS at 100, 200, or 500 ng/mL and triptolide at 0, 10, 25, 50, or 100 nmol/L; 10 μL CCK-8 reagent was then added, the plates were incubated for 3 h at 37°C, and absorbance was read at 450 nm after blank subtraction. This technical-well screen was used only to choose 10 nmol/L triptolide for subsequent experiments and did not support a formal cytotoxicity conclusion. Verified resource identifiers and explicitly unavailable catalogue or dilution details are listed in S3 Table.

For the initial three-group experiment, cells were assigned to Control, lipopolysaccharide (100 ng/mL), or lipopolysaccharide plus triptolide (10 nmol/L) conditions. Lipopolysaccharide and triptolide were added simultaneously in the combined-treatment group, and all conditions were maintained for 24 h. Total RNA was extracted with TRIzol (Thermo Fisher/Invitrogen, 10177071); 1 μg total RNA was reverse-transcribed with a Vazyme kit (R323-01), and IL6, TNF, IL1B, and S100A8 were measured on a LightCycler 480 instrument (Roche) using Vazyme qPCR master mix (Q311-02). Primer sequences are provided in S2 Table. Relative expression was calculated by the 2 − ΔΔCt method [43] with GAPDH as the reference gene. The archived project contained relative-expression plots but no sample-level Ct or ΔCt table; consequently, the displayed relative-expression analyses could not be independently re-estimated on the ΔCt scale and are treated as exploratory. All primer pairs produced single melting-curve peaks; each sample included a no-reverse-transcription control and a no-template control, and all no-RT Ct values were >35. DNase I treatment was used for S100A8 together with no-RT controls to limit genomic-DNA contamination. Protein was extracted in RIPA buffer with protease/phosphatase inhibitors; protein concentration was measured by BCA, and 20−30 μg per lane was separated by SDS-PAGE and transferred to PVDF. Membranes were blocked with rapid blocking buffer, incubated with primary antibodies overnight at 4°C and HRP-conjugated secondary antibody (Biosharp, BL003A) for 1 h, and detected with ECL (UUBIO, U10010B-2) on a ChemiDoc MP system (Bio-Rad). NF-κB p65 and phospho-NF-κB p65 were detected sequentially on the same membrane, with phospho-NF-κB detected first and total NF-κB detected after an approximately 40-min stripping step. Target proteins were acquired at 30-s exposure and the loading control at 5-s exposure; pilot loading-gradient experiments indicated a linear range (R^2^ > 0.95; approximately 5,000−40,000 in 8-bit grayscale). Densitometry used ImageJ with consistent regions of interest and background subtraction. For Figs 7 and 8, final protein normalization used β-actin, and p-NF-κB was normalized to total NF-κB. Initial-experiment antibodies included S100A8 (Proteintech, 15792–1-AP), NF-κB p65 (CST, 8242S), and phospho-NF-κB p65 (CST, 3033S); their batch-specific dilutions were not recoverable from the archive. The β-actin supplier, catalogue number, and dilution were also not recoverable (S3 Table). Full-field blot images, exposure pages, lane and crop mappings, and repeat-level source images underlying Figs 7 and 8 are provided in S1 Raw images.

For loss-of-function experiments, THP-1 cells were transfected with S100A8-targeting siRNA or Qiagen Negative Control siRNA (catalogue 1027310; 20 nmol product format) using a Lonza Nucleofector. The exact Nucleofector model, solution/kit, and program were not recoverable from the archived record and are disclosed in S3 Table. The S100A8-targeting and negative-control siRNA sequences are provided in S2 Table. Forty-eight hours after transfection, cells were assigned to Control, triptolide, lipopolysaccharide, or lipopolysaccharide plus triptolide conditions. In the combined-treatment groups, lipopolysaccharide and triptolide were added simultaneously, all groups had the same final DMSO concentration, and treatment was maintained for 24 h before harvesting. A separate 2 × 2 experiment compared siRNA and lipopolysaccharide effects on CD74 (CST, 77274; 1:1,000), HLA-DRα (Proteintech, 17221–1-AP; 1:2,000), and S100A8. All loss-of-function immunoblots comprised three independent experiments. Antibody details and record limitations are provided in S3 Table.

### Cellular thermal shift assay

THP-1 cells were treated with vehicle or 10 nmol/L triptolide and divided across 40, 46, 52, 58, 64, and 70°C. Samples were heated for 3 min, cooled for 3 min, transferred to ice, lysed by freeze-thawing, and centrifuged to remove aggregated protein. Soluble S100A8 was measured by immunoblot and normalized within treatment to the 40°C signal [44]. CETSA was interpreted as compatible with target engagement or indirect stabilization, not proof of direct binding.

### Statistical analysis

Tests were two-sided. FDR was controlled with the Benjamini-Hochberg procedure when multiple features or pathways were tested. Initial three-group in vitro comparisons used one-way ANOVA with Tukey adjustment on the archived relative-expression values; because sample-level Ct/ΔCt values were unavailable, these RT-qPCR P values are exploratory and could not be independently verified on the preferred ΔCt scale. Loss-of-function data used experimental repeat as a blocking factor in siRNA×lipopolysaccharide or siRNA×lipopolysaccharide×triptolide factorial linear models; prespecified contrasts were Holm adjusted within endpoint. Effect estimates are reported with 95% confidence intervals. P < 0.05 or FDR < 0.05 was considered statistically significant, as appropriate. Replicate-aware factorial models and prespecified contrasts are reported in S7 Table.

## Results

### Log2-RMA WGCNA supports *S100A8* within the strongest sepsis-positive module

The corrected log2 RMA/maximum-probe/limma analysis tested 11,719 genes and identified 1,508 DEGs: 492 were upregulated and 1,016 downregulated. *S100A8* was increased in sepsis (log2 fold change 1.875; FDR 2.15 × 10 − 171). The WGCNA rerun retained all 802 samples (760 sepsis and 42 normal controls) and all 11,719 finite, nonzero-variance genes. Eight non-grey modules were identified. Four non-grey modules were positively associated with sepsis after correction, with black showing the strongest positive association (713 genes; r = 0.409; P = 1.24 × 10 − 33; FDR = 3.72 × 10 − 33); red, pink, and brown were also positively associated, whereas turquoise, blue, and green were negatively associated. The grey unassigned-gene eigengene showed a weak positive association and yellow was not significant; all tests are displayed in Fig 1B. *S100A8* belonged to black (module membership 0.700; gene significance 0.795). The black module shared 265 genes with the corrected DEG set (Fig 1D). Fifty-two GO terms survived FDR correction and included response to bacterium, regulation of inflammatory response, myeloid leukocyte activation, and granulocyte-related compartments. None of 241 tested KEGG pathways survived FDR correction (Fig 1E,1F). In the mean-probe sensitivity network, the strongest sepsis-positive module was red (757 genes; r = 0.405; P = 4.77 × 10 − 33; FDR = 1.91 × 10 − 32); *S100A8* remained in that module (module membership 0.669; gene significance 0.795), and 241 module genes overlapped the corrected DEG set. The main black and mean-probe red modules shared 519 genes (Jaccard index 0.546), supporting the same S100A8-associated co-expression signal while showing that module color and boundaries depend on aggregation.

**Fig 1:**
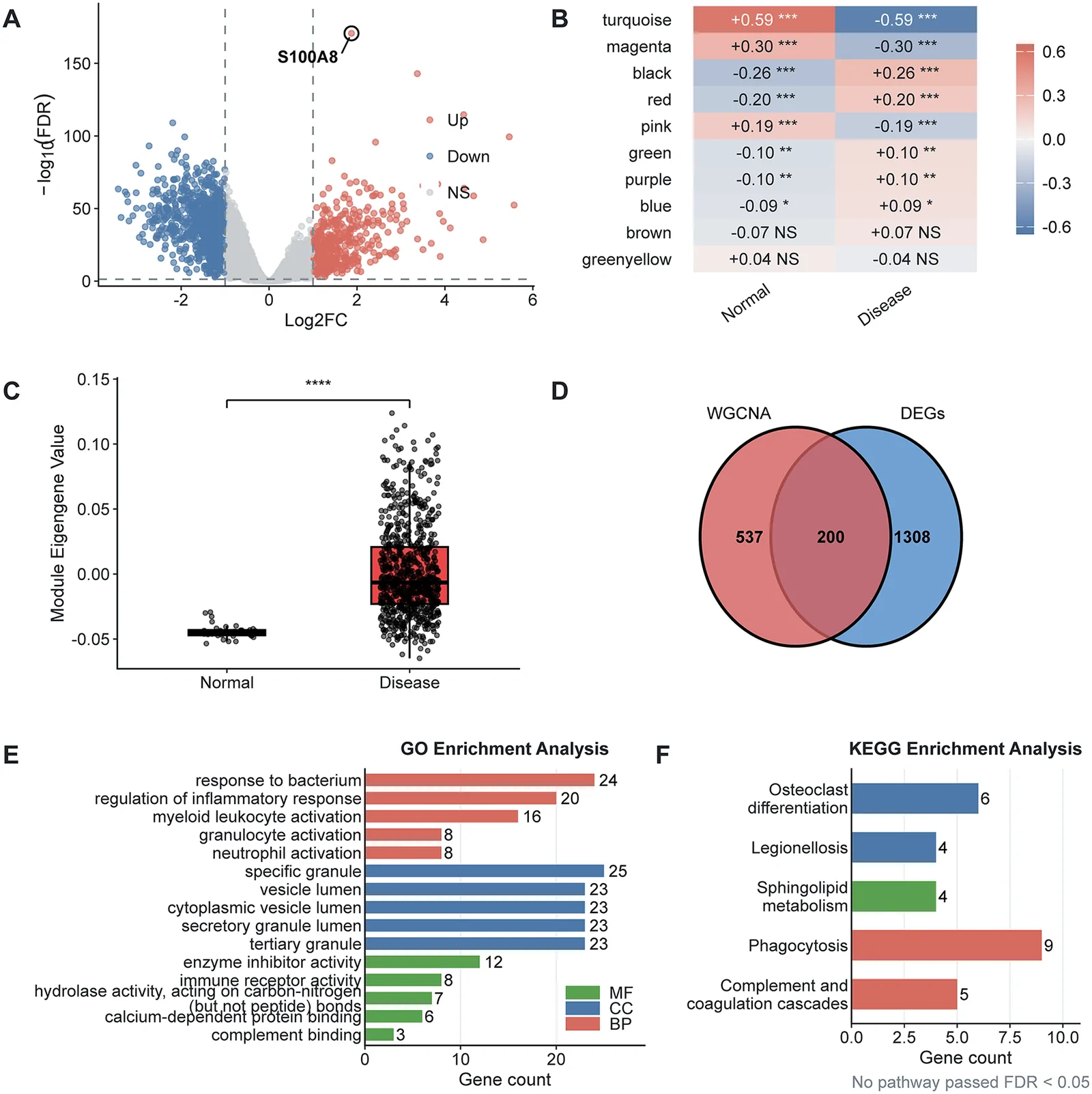
Corrected differential expression and log2-RMA WGCNA prioritize *S100A8* within a sepsis-associated black module. (A) Limma volcano plot using log2 RMA expression; red and blue indicate upregulated and downregulated genes at FDR < 0.05 and |log2 fold change| > 1. *S100A8* is labeled. (B) Correlations of all module eigengenes with sepsis status; raw correlations and BH-adjusted FDR values are shown. (C) Black-module eigengene distribution in 42 normal-control and 760 sepsis samples; the displayed Wilcoxon P value is descriptive. (D) Intersection of 713 black-module genes and 1,508 corrected DEGs: 448 module-only, 265 overlapping, and 1,243 DEG-only genes. (E) Representative FDR-significant GO terms among the 265 genes. (F) Highest-ranked nominal KEGG terms; none of 241 tested pathways passed FDR < 0.05.

### Class-balanced analyses support *S100A8* prioritization but not a deployable multigene model

In corrected full-cohort descriptive rankings, *S100A8* appeared among the five highest-ranked features for LASSO, glmBoost, XGBoost, and Ranger; the broader selected sets differed by method (S1 Fig). In leakage-controlled repeated cross-validation, high case-control separation persisted across weighting and training-fold downsampling strategies (Fig 2A-2C). In 1,000 balanced 1:1 samples, the median *S100A8* AUC was 1.000 (empirical 95% interval 0.991–1.000), and the standardized mean difference was 4.32 (95% interval 3.80–4.71). The stratified-bootstrap AUC was 0.9986 (95% interval 0.9963–0.9996).

**Fig 2:**
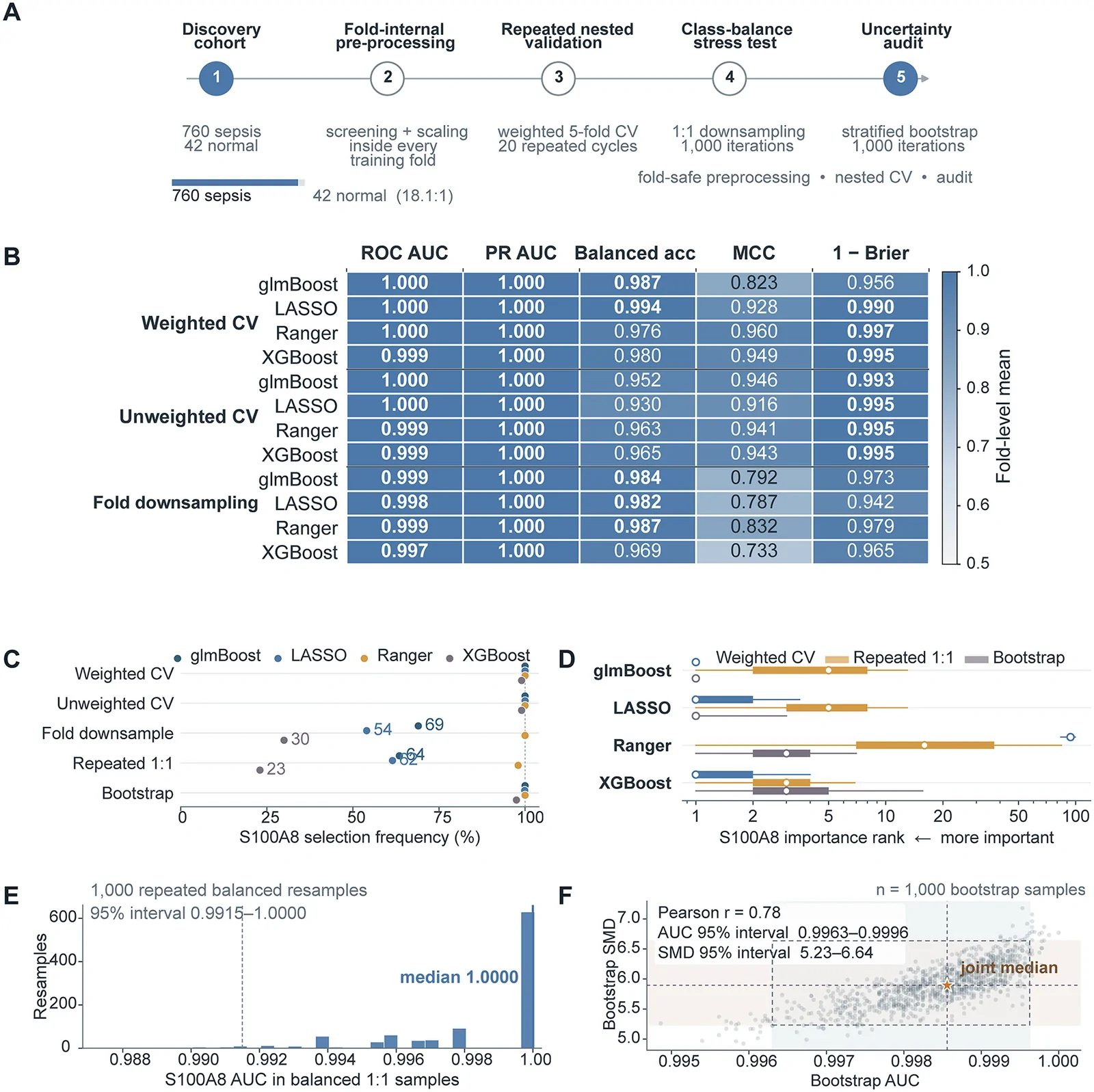
Leakage-controlled class-imbalance analyses support feature prioritization rather than clinical deployment. (A-C) Performance across class-weighted, unweighted, and training-fold-downsampled repeated nested cross-validation. (D) *S100A8* feature-selection frequency by model under sensitivity strategies. (E) Distribution of *S100A8* AUC across 1,000 balanced 1:1 samples. (F) Calibration and selection-stability summary. Preprocessing and balancing were restricted to training folds; outer folds were used only for validation.

Feature stability was model- and sampling-dependent. Under 200 repeated balanced feature-selection runs, *S100A8* selection frequencies were 0.615 for LASSO, 0.635 for glmBoost, 0.980 for Ranger, and 0.230 for XGBoost; only 0.210 of resamples selected *S100A8* in all four models. Calibration slopes also varied despite high AUCs (Fig 2D-2F). These results support machine learning as a prioritization device and argue against presenting the fitted signatures as externally validated clinical predictors.

### Clinical-comparator and outcome analyses define the boundary of the signal

In the prespecified sepsis-versus-healthy cohorts, *S100A8* AUCs were 0.944 (GSE9692), 0.889 (GSE26440), 1.000 (GSE28750), and 0.983 (GSE69528) (Fig 3A). Clinically similar comparisons were heterogeneous: AUC was 0.727 (95% CI 0.654–0.802) against non-infectious ICU controls, 0.932 (0.821–1.000) against cardiogenic shock, 0.927 (0.782–1.000) against postoperative sterile inflammation, and 0.972 (0.937–0.996) against uninfected chronic-disease controls (Fig 3B-3D; S4 Table). In the postoperative contrast, median *S100A8* expression remained higher in sepsis (14.826; IQR 14.753–14.877) than after sterile surgery (14.447; IQR 14.031–14.598). The ROC direction was fixed a priori toward higher expression in sepsis and was not automatically reversed by pROC. Thus, the high AUC reflects separation in the same direction as the healthy-control comparison, with a smaller between-group difference. Decision curves showed context-dependent net benefit and did not justify a universal threshold (Fig 3E; S2 Fig). The GSE131411 estimate used only T1, the first ICU sample per eligible donor.

**Fig 3:**
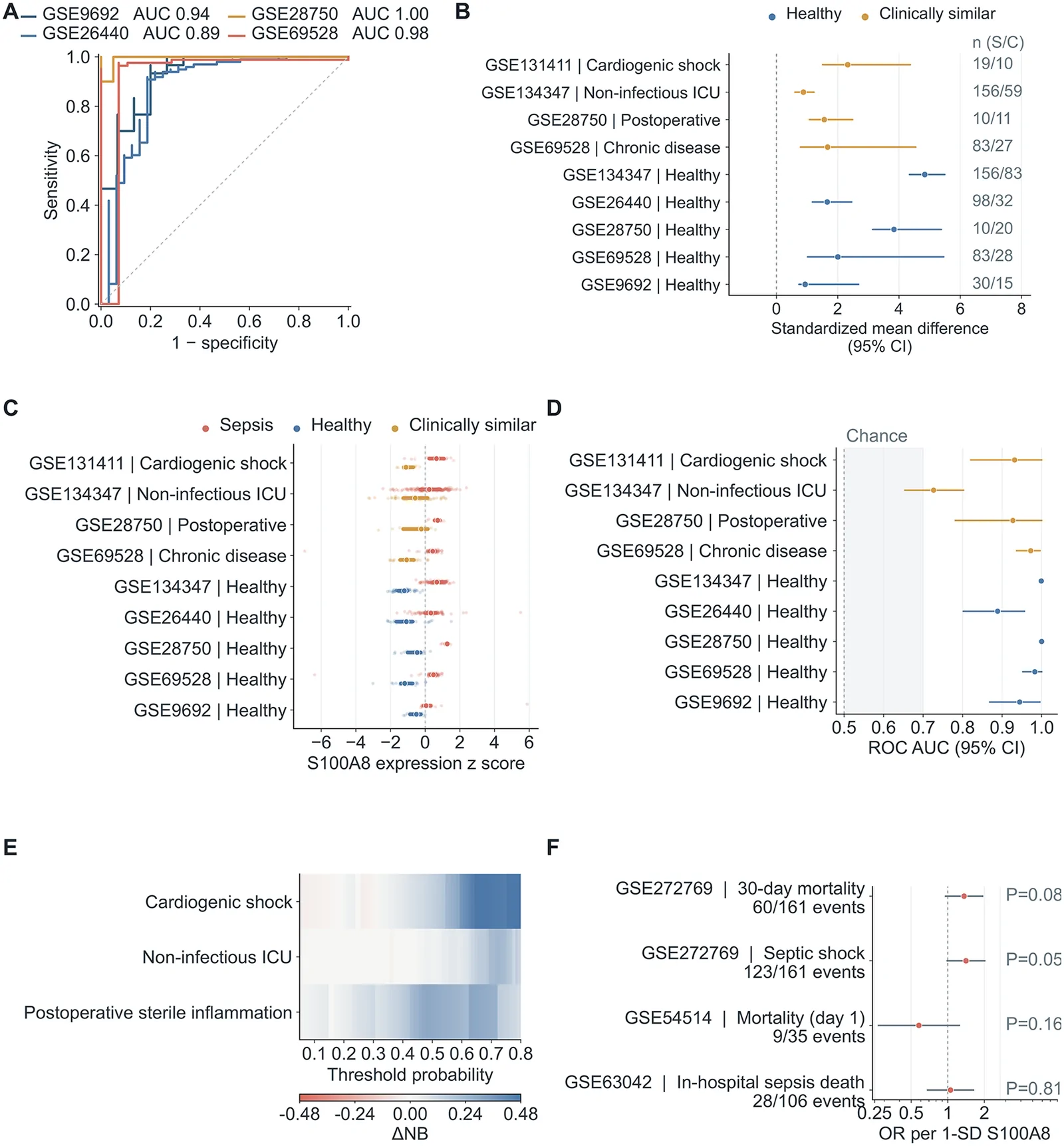
Clinical-comparator and outcome analyses define the scope of *S100A8.* (A) ROC curves in the prespecified sepsis-versus-healthy external cohorts. (B) *S100A8* distributions in clinically similar comparator cohorts. (C) Standardized effect sizes across healthy and clinically similar comparisons. (D) ROC curves for non-infectious ICU, cardiogenic shock, postoperative sterile inflammation, and uninfected chronic-disease controls. For the postoperative comparison, expression was higher in sepsis than in sterile inflammation; the ROC direction was fixed toward higher *S100A8* in sepsis, so AUC = 0.927 was not produced by automatic orientation reversal. (E) Decision-curve analysis; thresholds are cohort specific and exploratory. (F) Associations with 30-day mortality, septic shock, and mortality in available cohorts. AUC, area under the curve; CI, confidence interval.

*S100A8* was not a stable outcome marker. In GSE272769, the standardized odds ratios were 1.36 (95% CI 0.96–1.94; P = 0.084) for 30-day mortality and 1.41 (0.99–2.02; P = 0.057) for septic shock. Mortality estimates were also null in GSE54514 (OR 0.58, 0.27–1.24; P = 0.161) and GSE63042 (OR 1.05, 0.68–1.63; P = 0.814) (Fig 3F). *S100A8* did not correlate with APACHE score in GSE54514 (Spearman ρ = −0.073; P = 0.677) and did not differ by treatment response in GSE110487 (P = 0.982). In GSE63042, *S100A8* remained associated with sepsis versus sterile SIRS within cohort (OR per SD 2.27, 95% CI 1.36–3.77; P = 0.00165), illustrating that performance depends on the sampled comparator.

Sampling-time metadata were incomplete for 733 of 760 discovery sepsis samples. Excluding three explicitly annotated infection-event samples changed the *S100A8* AUC only from 0.99837 to 0.99836. Among 479 samples with MARS annotation, *S100A8* varied across MARS1-MARS4 (Kruskal-Wallis P < 2.2 × 10 − 16), confirming substantial endotype heterogeneity.

### Donor-aware single-cell analysis localizes *S100A8* to an emergency-myeloid, HLA-DR-low state

The GSE167363 PBMC atlas contained T-cell, B-cell, NK-cell, and monocyte compartments (Fig 4A-4C). Unsupervised monocyte reclustering identified three states. C0 combined an emergency-myeloid/immature-neutrophil-like program with inflammatory and HLA-DR-low features; C1 resembled VCAN-positive classical monocytes; C2 had a lymphoid-like contamination profile (Fig 4D,4E). The C0 fraction was higher in sepsis by 0.641 (95% CI 0.493–0.789), but the donor-label permutation FDR was 0.095; this abundance result was therefore treated as suggestive.

**Fig 4:**
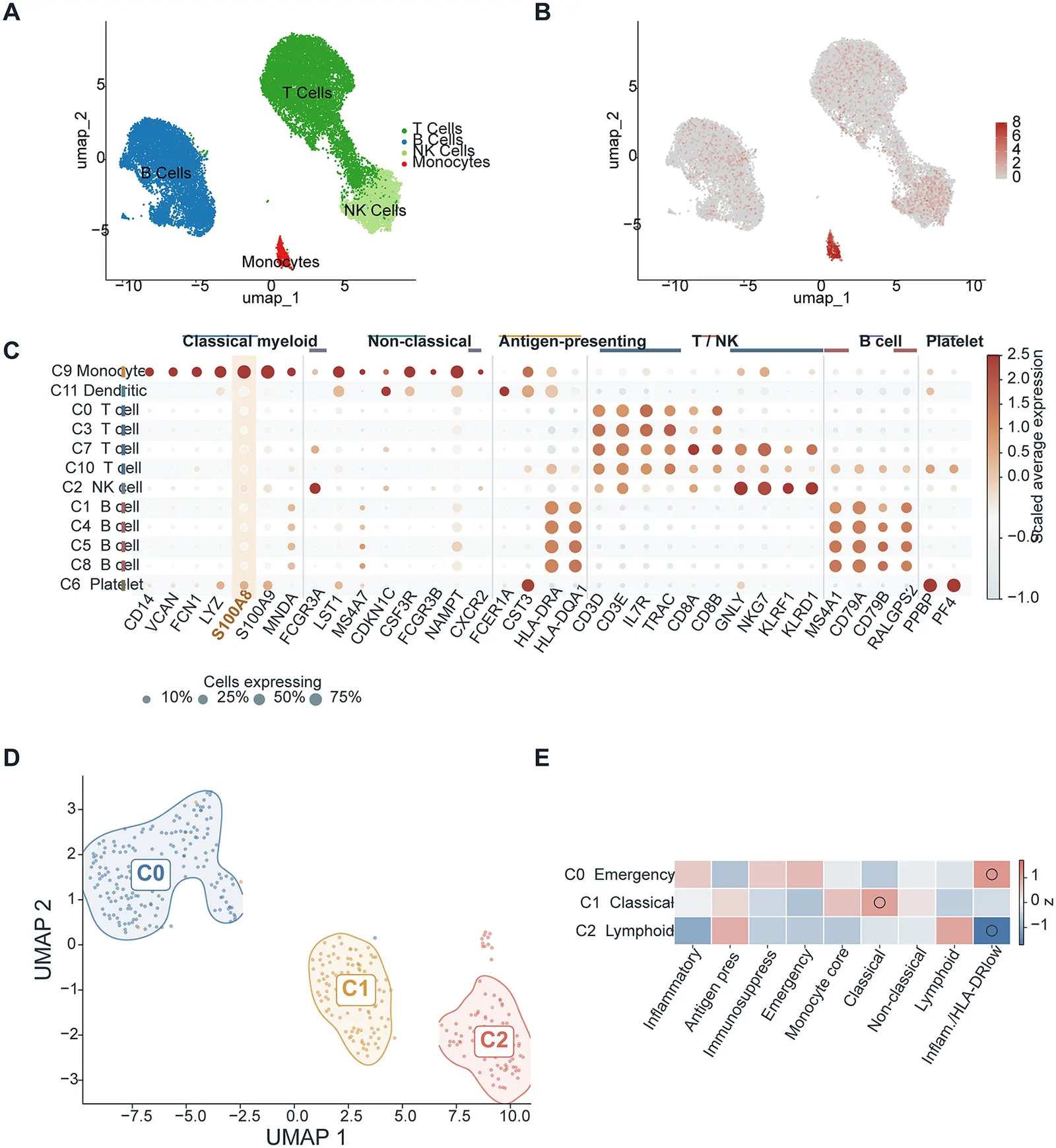
Donor-aware localization of *S100A8* within the GSE167363 PBMC atlas. (A) UMAP of major cell classes. (B) *S100A8* expression in UMAP space. (C) Canonical-marker dot plot across clusters C0-C11. (D) Unsupervised monocyte clusters C0-C2. (E) State-program scores showing an emergency-myeloid/immature-neutrophil-like, inflammatory, HLA-DR-low C0 state; a VCAN-positive classical C1 state; and a lymphoid-like C2 contamination profile. Cell-level plots are descriptive; formal differential expression used donor-level pseudo-bulk profiles.

At the primary 20-monocyte threshold, donor-level pseudo-bulk inference included two control and four sepsis donors. *S100A8* (log2 fold change 3.31; FDR 2.75 × 10 − 5), *S100A9* (2.82; FDR 3.40 × 10 − 4), *S100A12* (3.63; FDR 7.26 × 10 − 6), and *ARG1* (5.82; FDR 0.00305) were increased, whereas *HLA-DRA* was decreased (−2.37; FDR 0.0277). *CD74* (−1.79; FDR 0.289) and *HLA-DPB1* (−1.48; FDR 0.278) changed in the same direction but were not FDR significant. Donor-level *RELA*, *NFKB1*, *STAT3*, and other tested transcription-factor activities did not survive FDR correction (S3 Fig).

### Independent, non-circular programs show cross-dataset concordance with heterogeneity

The 30-gene emergency-myeloid signature excluded *S100A8*, and the sensitivity signature excluded *S100A8* and *S100A9*. In GSE205672, sepsis donors had higher *S100A8* (FDR 2.97 × 10 − 5) and emergency-myeloid score (FDR 2.97 × 10 − 5) and lower antigen-presentation score (FDR 0.00559); the NF-κB-related score did not differ (P = 0.926) (Fig 5D,5E; S4 Fig). Across donors, *S100A8* correlated positively with the emergency-myeloid score in GSE205672 (ρ = 0.89) and GSE167363 (ρ = 0.94), and inversely with antigen presentation in GSE205672 (ρ = −0.46), GSE167363 (ρ = −0.94), and SCP548 (ρ = −0.30) (Fig 5F).

**Fig 5:**
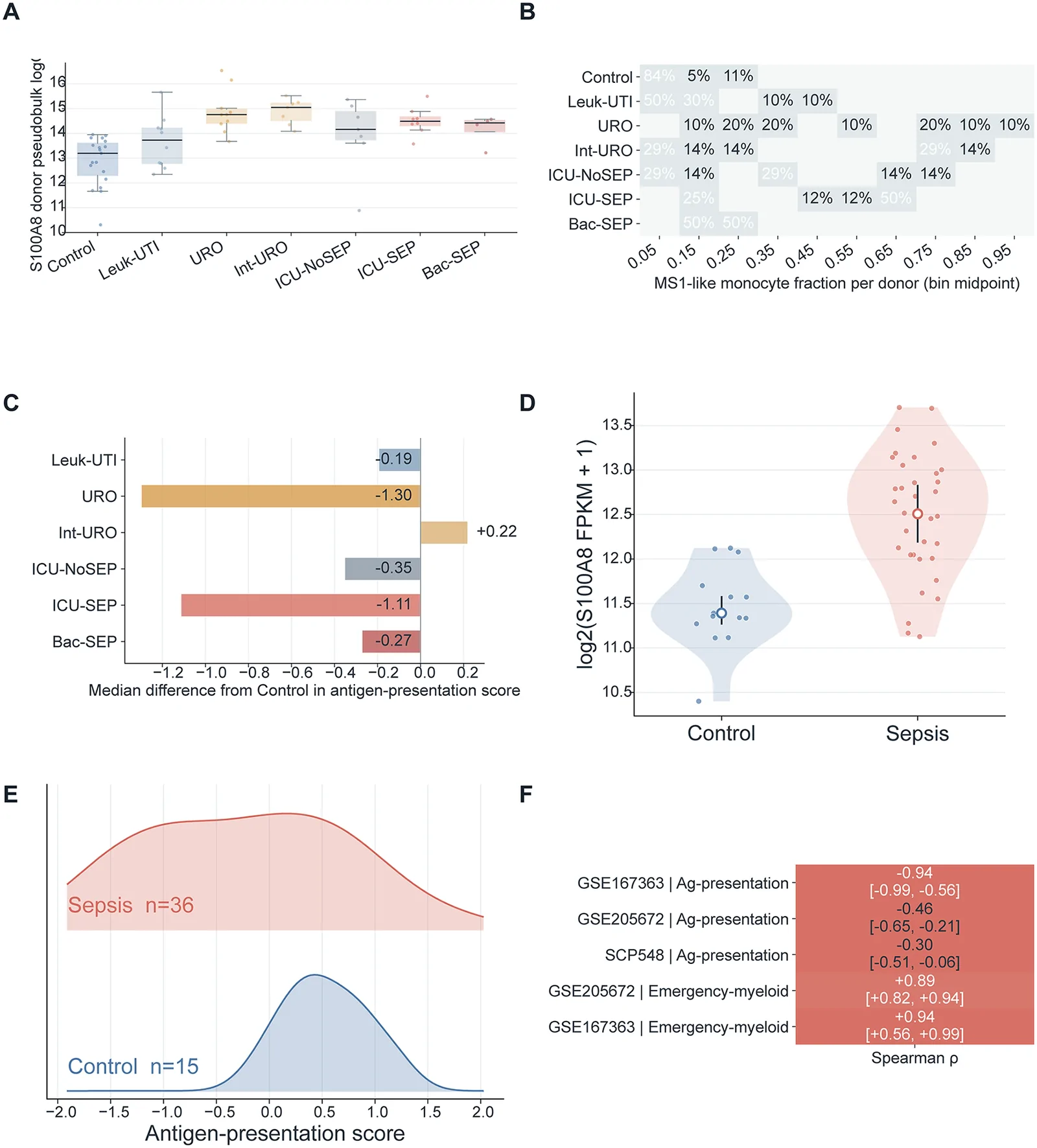
Independent single-cell datasets provide heterogeneous support for an S100A8-associated myeloid/antigen-presentation axis. (A) SCP548 donor pseudo-bulk *S100A8* across clinical groups. (B) Donor distribution of the MS1-like monocyte fraction. (C) Median antigen-presentation score difference from control by SCP548 group. (D,E) GSE205672 donor *S100A8* and antigen-presentation scores. (F) Cross-dataset Spearman correlations of *S100A8* with emergency-myeloid and antigen-presentation programs. State signatures exclude *S100A8*; the stricter sensitivity signature also excludes *S100A9*.

SCP548 showed graded and heterogeneous donor distributions across urinary-tract infection, urosepsis, ICU sepsis, non-septic ICU illness, and bacterial sepsis groups (Fig 5A-5C). For example, ICU sepsis versus non-septic ICU *S100A8* did not differ significantly after correction. Thus, the external analyses support context-dependent concordance between *S100A8* and a myeloid/antigen-presentation axis, not uniform independent validation or a sepsis-specific cell state.

### Matched docking supports site compatibility, whereas molecular dynamics shows limited pose stability

L1000CDS2 prioritized 16-hydroxytriptolide, whereas all subsequent simulations and experiments used triptolide (Fig 6A,6B). The compounds were structurally similar (Morgan Tanimoto 0.831; MACCS Tanimoto 0.885), and the maximum common substructure covered all 26 triptolide heavy atoms and 26 of 27 16-hydroxytriptolide heavy atoms. Matched surface-wide docking placed both compounds in the same predicted S100A8 homodimer-interface region in both A/C and B/D receptor representations. Common-scaffold RMSDs were 0.13–0.62 Å and contact-set Jaccard indices 0.90–1.00 (Fig 6C-6F). These results support predicted site compatibility but not functional equivalence or experimental binding.

**Fig 6:**
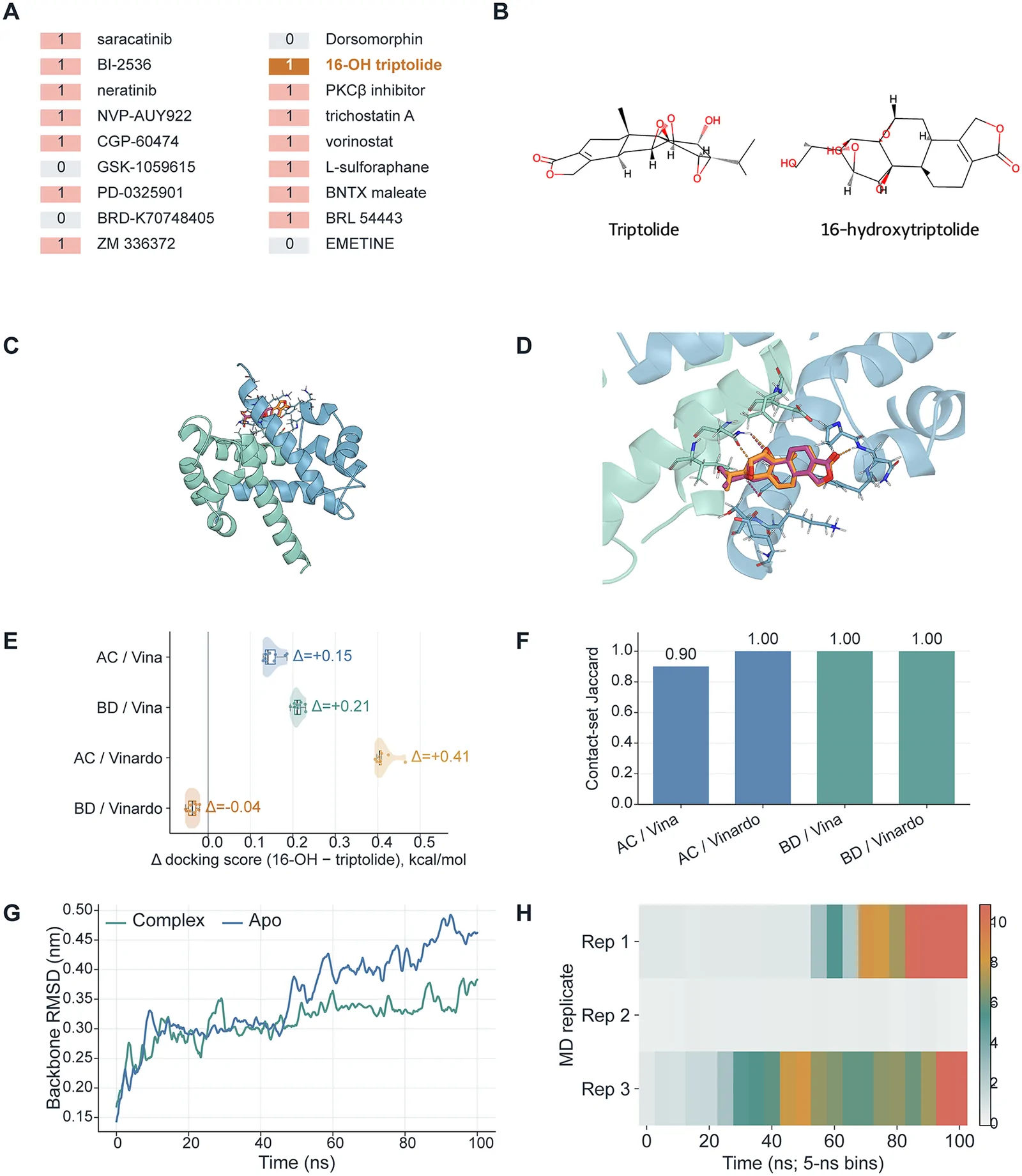
Exploratory compound prioritization, matched docking, and replicate-aware molecular dynamics. (A) L1000CDS2 candidate screen highlighting 16-hydroxytriptolide. (B) Structures of triptolide and 16-hydroxytriptolide. (C,D) Representative triptolide docking pose and interface contacts in the S100A8 homodimer (PDB 5HLO). (E) Matched score differences between compounds across A/C and B/D receptors and Vina/Vinardo scoring. Scores from different functions are not directly comparable. (F) Contact-set Jaccard indices. (G) Complex and apo backbone RMSD across three 100-ns replicates. (H) Triptolide displacement from the original pocket, showing retention in replicate 2 and migration in replicates 1 and 3. Docking and dynamics do not establish direct binding.

Across three 100-ns complex and three matched apo simulations, both protein systems remained folded. In the 20–100-ns window, the median of replicate-mean backbone RMSDs was 0.323 nm for the complex and 0.380 nm for apo (S5 Fig). Triptolide behavior was replicate dependent: mean displacement from the original pocket was 5.16, 0.44, and 6.25 nm in replicates 1–3. Replicate 2 remained within 1 nm of the starting pocket in 100% of analyzed frames, whereas replicate 1 remained there in 43.2% and replicate 3 in 0%. Nevertheless, replicates 1 and 3 often remained in contact with other protein surfaces (Fig 6G,6H; S6 Fig). PCA showed multiple basins but also strong replicate effects (S7 Fig). Thus, the simulations do not support a single, uniformly stable binding mode. Docking retained the zinc ions present in 5HLO, whereas zinc was removed before molecular dynamics because a validated zinc-parameterization protocol for this system was not available; this difference limits direct comparison between the docked metal-bound site and the simulated system.

### THP-1 experiments provide bounded functional support and a negative dependency result

In the three-group THP-1 experiment, lipopolysaccharide increased IL6, TNF, IL1B, and S100A8 transcripts and increased S100A8 and NF-κB phosphorylation readouts; triptolide attenuated these changes (Fig 7A-7C). CETSA showed greater soluble S100A8 signal at some higher temperatures after triptolide treatment (Fig 7D,7E), a pattern compatible with target engagement or indirect stabilization but not proof of direct binding. The CCK-8 screen used technical wells only and served solely to select the 10-nmol/L working concentration (S8 Fig).

**Fig 7:**
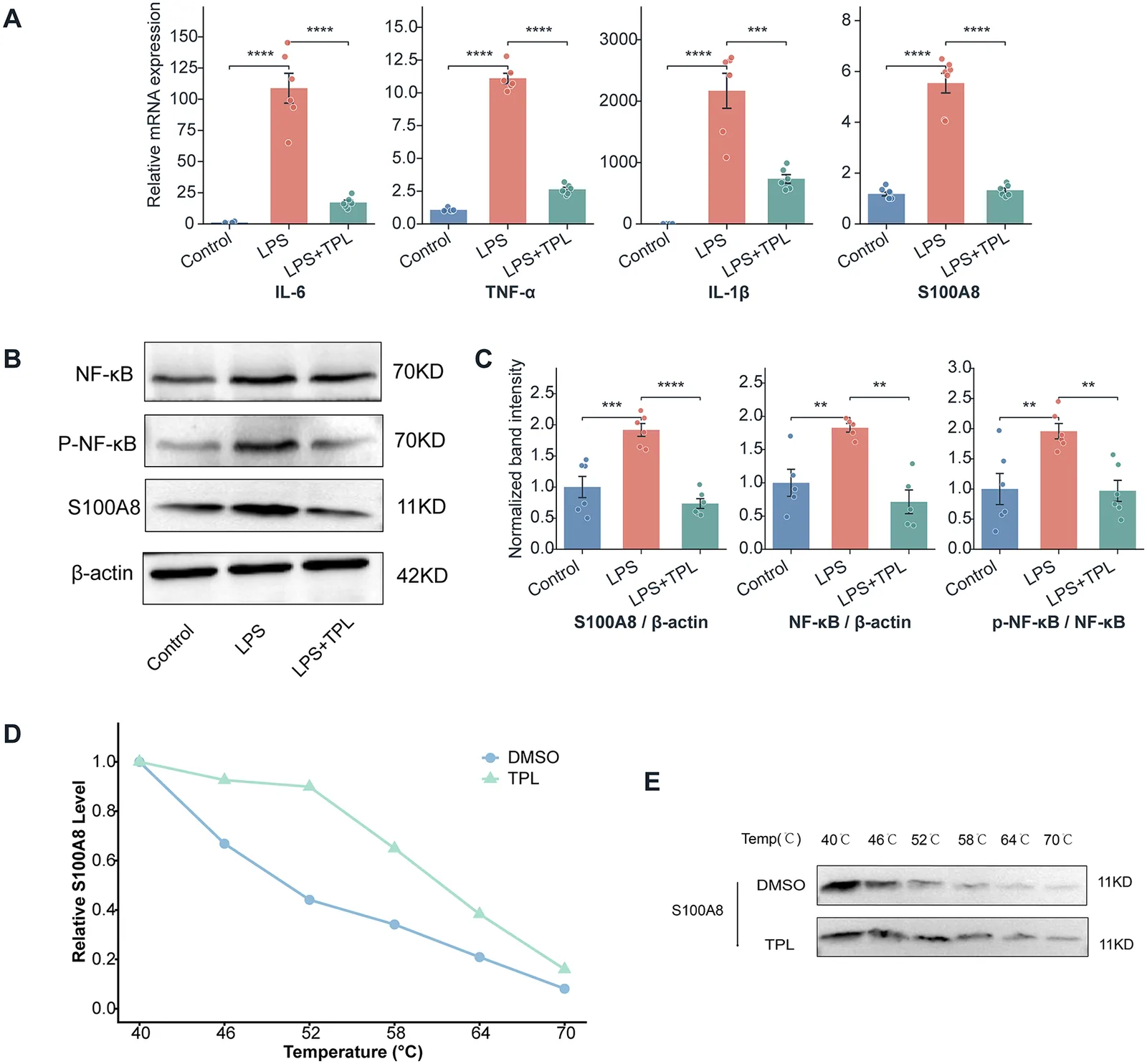
Triptolide-associated inflammatory readouts and S100A8 thermal stability in THP-1 cells. (A) IL6, TNF, IL1B, and S100A8 RT-qPCR in Control, LPS, and LPS + TPL groups. (B) Representative immunoblots. (C) S100A8/β-actin, total NF-κB/β-actin, and p-NF-κB/total NF-κB quantification. (D) CETSA soluble-S100A8 curves across 40-70°C. (E) Representative CETSA blots. Points denote independent experiments as specified in the source data; bars show means with the displayed uncertainty. CETSA is compatible with target engagement or indirect stabilization and does not prove direct binding.

S100A8 knockdown was confirmed under control and lipopolysaccharide conditions. In siNC cells, lipopolysaccharide reduced CD74 by 0.371 normalized units (95% CI −0.466 to −0.276) and HLA-DRα by 0.429 (−0.516 to −0.341). Knockdown attenuated these reductions: the siRNA×lipopolysaccharide interaction was + 0.337 (95% CI 0.285–0.390; P = 4.08 × 10 − 6) for CD74 and +0.374 (0.290–0.458; P = 3.59 × 10 − 5) for HLA-DRα (Fig 8D-8G). HLA-DPB1 was not tested.

**Fig 8:**
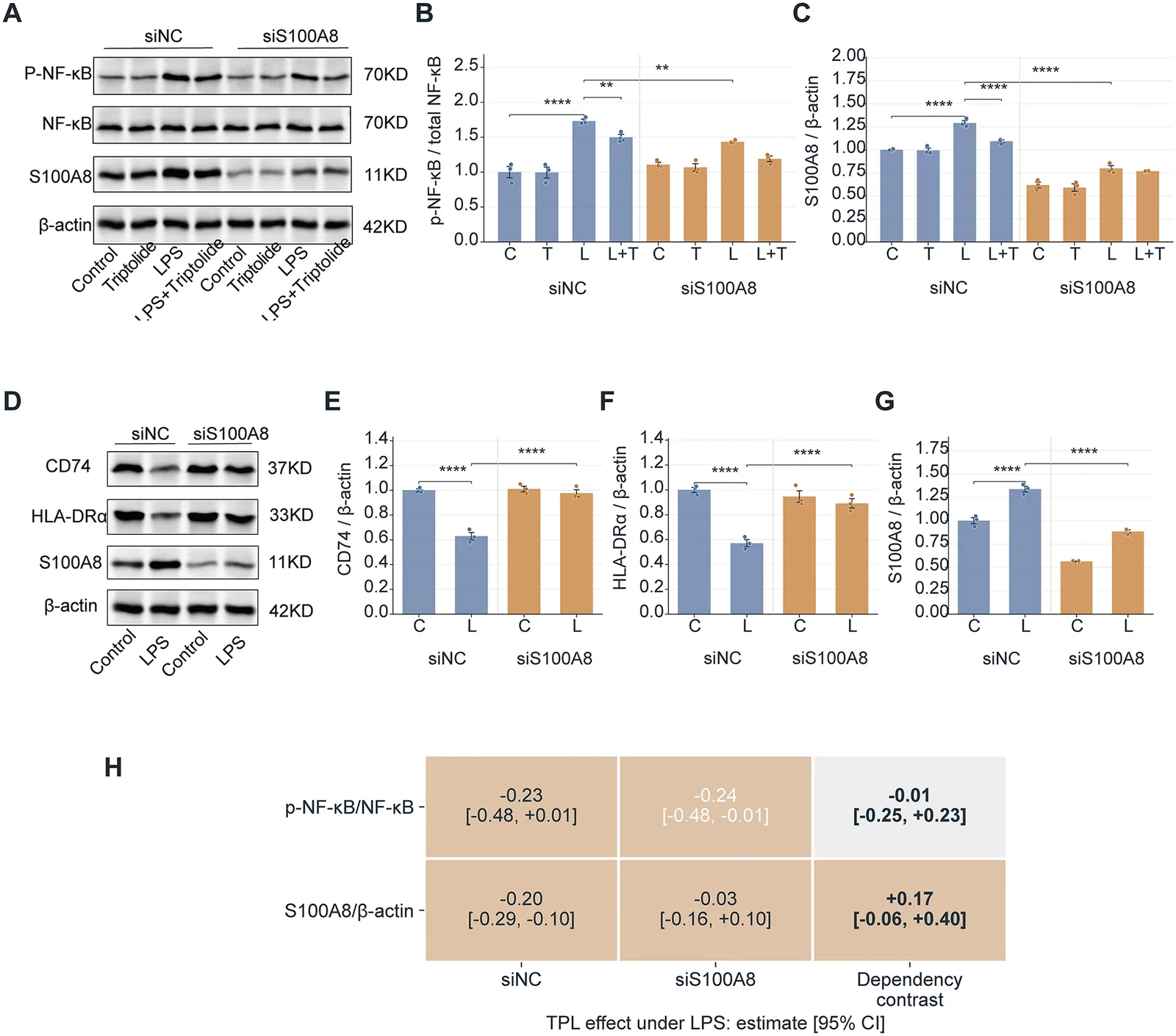
S100A8 knockdown links the myeloid state to antigen-presentation and NF-κB readouts without establishing triptolide dependency. (A) Eight-condition siNC/siS100A8×Control/TPL/LPS/LPS + TPL immunoblots. (B,C) p-NF-κB/total NF-κB and S100A8/β-actin across three independent experiments. (D) Four-condition siRNA × LPS immunoblots. (E-G) CD74/β-actin, HLA-DRα/β-actin, and S100A8/β-actin. (H) TPL effects under LPS and the dependency contrast. The p-NF-κB dependency contrast was −0.011 (95% CI −0.249 to 0.226; P = 0.857); the three-way interaction was P = 0.848.

S100A8 knockdown also attenuated the lipopolysaccharide-associated p-NF-κB/total NF-κB increase (interaction −0.406, 95% CI −0.600 to −0.211; P = 0.000522) (Fig 8A-8C). However, under lipopolysaccharide, triptolide changed this ratio by −0.233 in siNC cells and −0.244 in siS100A8 cells; the dependency contrast was −0.011 (95% CI −0.249 to 0.226; P = 0.857), and the three-way interaction was null (P = 0.848) (Fig 8H). The data therefore do not show that the triptolide effect requires S100A8.

## Discussion

The integrated analyses converge on a focused conclusion: *S100A8* is a reproducible sepsis-associated transcript and a marker of an emergency-myeloid, HLA-DR-low axis. This interpretation is supported by corrected limma differential expression, its membership in the strongest sepsis-positive module in the log2-RMA WGCNA rerun, preservation of that assignment in the mean-probe sensitivity network, stability under balanced sampling, donor-level monocyte pseudo-bulk inference, non-circular state signatures, and targeted loss-of-function readouts. It is bounded by clinically similar comparators, null outcome analyses, small single-cell donor numbers, heterogeneous external state projections, variable molecular-dynamics behavior, and a negative triptolide-dependency experiment.

The very high AUCs against healthy controls are biologically informative but clinically insufficient. Performance fell against non-infectious ICU illness. In the postoperative comparison, *S100A8* remained higher in sepsis than in sterile inflammation and the fixed-direction AUC was 0.927; this result therefore indicates separation between these sampled groups, not an orientation-reversed ROC and not universal sepsis specificity. The contrast-dependent performance is compatible with *S100A8* reflecting a broader acute myeloid inflammatory response. The MARS endotype differences and incomplete sampling-time information add further heterogeneity. Accordingly, we avoid a single clinical cutoff and do not compare *S100A8* with unavailable routine biomarkers. Prospective studies must enroll consecutive patients with clinically relevant mimics and prespecify timing, diagnostic adjudication, and thresholds. Moreover, calibration, precision-recall, Brier-score, and decision-curve summaries from case-control cohorts remain conditional on the sampled case fractions and cannot establish population-level clinical utility.

Donor-level single-cell analysis reduced the pseudoreplication problem, consistent with statistical guidance that cells from the same individual should not be treated as independent replicates [31], but exposed its own limitation: only two control and four sepsis donors met the primary monocyte threshold. Within this small dataset, increased *S100A8* and decreased *HLA-DRA* were FDR significant, whereas *CD74*, *HLA-DPB1*, and transcription-factor activities were not. The direction of this myeloid/antigen-presentation axis agrees with prior sepsis single-cell studies [11,12,15], but the independent signature and cross-dataset correlations do not establish that the state is identical across studies. SCP548 further demonstrates that the axis is not uniformly separated across every clinical subgroup. Transcriptional annotation also cannot establish suppressive function. Recent multi-cohort work likewise identifies conserved severity-associated myeloid responses across bacterial and viral infections, reinforcing that this axis is not uniquely sepsis specific [13].

The THP-1 perturbation provides targeted functional support. *S100A8* knockdown attenuated lipopolysaccharide-associated reductions in *CD74* and HLA-DRα and altered the p-NF-κB/total NF-κB readout. These results link the transcriptomic state to perturbable protein phenotypes in a simplified model. They do not reproduce patient sepsis, establish *HLA-DPB1* regulation, demonstrate NF-κB nuclear translocation or transcriptional activity, or prove that *S100A8* is a therapeutic target. Primary-cell, rescue, and orthogonal pathway assays are needed.

The compound analyses require equally strict separation. Sixteen-hydroxytriptolide was the computational hit; triptolide was the structurally related compound used experimentally. Matched docking supports predicted S100A8-site compatibility but not equivalence. CETSA is compatible with engagement or indirect stabilization, while molecular dynamics shows that the starting pocket pose is stable in only one of three trajectories. Most importantly, triptolide retained a similar NF-κB phosphorylation effect after S100A8 knockdown. The present evidence therefore does not establish direct S100A8 binding or an S100A8-dependent mechanism for triptolide. The different metal treatment in docking and molecular dynamics further prevents the simulations from being treated as a direct stability test of the docked zinc-bound complex.

Further limitations include retrospective public datasets, marked discovery-class imbalance, incomplete patient identifiers and timing, platform and phenotype heterogeneity, maximum-probe aggregation for multi-probe genes, no external validation of a multigene predictor, and no harmonized CRP, procalcitonin, interleukin-6, lactate, SOFA, or organ-failure data. Although the WGCNA rerun used the final log2 RMA matrix and retained all eligible samples and genes, module labels and boundaries remain specification dependent. The mean-probe sensitivity analysis retained *S100A8* in the strongest sepsis-positive module but changed its color, size, and DEG overlap; the 50% MAD sensitivity network did not retain *S100A8* because it fell below the prespecified variability threshold. Molecular docking and 3 × 100-ns simulations remain computational hypotheses, and their differing zinc treatment limits direct comparison. The archived qPCR materials lacked sample-level Ct/ΔCt values, preventing independent re-analysis on the preferred ΔCt scale. The CCK-8 screen used technical replicates, and all loss-of-function experiments used one cell line and three independent repeats.

Despite these limitations, the project illustrates how negative and heterogeneous results can sharpen a multi-omics conclusion. Rather than claiming a diagnostic biomarker or drug mechanism, the integrated evidence prioritizes an S100A8-associated emergency-myeloid, HLA-DR-low state for prospective clinical characterization and mechanistically specific perturbation.

## Conclusion

Multi-cohort transcriptomics and donor-aware single-cell analyses prioritize *S100A8* within a sepsis-associated emergency-myeloid, HLA-DR-low state. Clinical specificity, prognosis, direct compound binding, S100A8-dependent triptolide action, and therapeutic relevance remain unestablished.

## Supporting information

S1 Fig Descriptive feature-prioritization analyses using the 265 genes shared by the final log2-RMA WGCNA black module and corrected DEG set. LASSO diagnostics, top-ranked features from LASSO, glmBoost, XGBoost, and random forest, and TreeSHAP summaries are shown. *S100A8* was among the top five features in all four descriptive rankings. These full-discovery-cohort plots are not external model validation. The leakage-controlled Fig 2 analysis performs feature screening within each training fold and does not use this fixed overlap as its input.
(PDF)

S2 Fig Clinical and outcome sensitivity analyses. Precision-recall, calibration, decision-curve, mortality, severity, and treatment-response results are displayed for cohorts with usable metadata. Thresholds are exploratory and cohort specific.
(PDF)

S3 Fig Donor composition, pseudo-bulk robustness, and transcription-factor activity in GSE167363. Donor cell counts, threshold sensitivity, leave-one-donor-out summaries, and donor-level TF results are shown. No tested TF activity passed FDR < 0.05.
(PDF)

S4 Fig Non-circular myeloid-state sensitivity analyses. Results use the 30-gene signature excluding S100A8 and a stricter signature excluding S100A8 and S100A9; cross-dataset emergency-myeloid, antigen-presentation, and NF-κB score associations are shown.
(PDF)

S5 Fig Replicate-aware S100A8-triptolide complex and apo system metrics. Backbone RMSD, chain distance, RMSF, radius of gyration, solvent-accessible surface area, and inter-chain hydrogen bonds are summarized for three 100-ns trajectories per condition. Frames are not treated as independent replicates.
(PDF)

S6 Fig Triptolide trajectory behavior and protein contacts. Unwrapped ligand RMSD, displacement from the original pocket, PBC-aware minimum distance, contact counts, ligand-protein hydrogen bonds, and residue-contact occupancies are shown by replicate.
(PDF)

S7 Fig Pooled-basis principal-component and free-energy-landscape analyses of the six molecular-dynamics trajectories. Separation of conditions is intertwined with replicate-specific sampling and is not interpreted as a definitive ligand-induced transition.
(PDF)

S8 Fig Descriptive CCK-8 working-concentration screen. Values are technical-well summaries used only to select 10 nmol/L triptolide; they are not biological replicates and do not support a formal cytotoxicity claim.
(PDF)

S1 Table Public datasets, cohort composition, grouping, and analytic roles. (XLSX)

S2 Table RT-qPCR primer, S100A8-targeting siRNA, and negative-control siRNA sequences. (XLSX)

S3 Table Key resources, reagents, antibodies, software versions, author-confirmed treatment details, and explicitly unrecorded information. (XLSX)

S4 Table Direction-explicit clinical-comparator and external-validation results for *S100A8*, including group distributions, fixed ROC direction, likelihood ratios, calibration, and decision-curve status. Thresholds are cohort specific and exploratory, not universal clinical cutoffs.
(XLSX)

S5 Table Leakage-controlled class-balance performance and *S100A8* feature-stability results. (XLSX)

S6 Table Donor-level pseudo-bulk threshold, focus-gene, cell-count, and transcription-factor activity results. (XLSX)

S7 Table Replicate-aware in vitro factorial models and prespecified planned contrasts underlying Figs 7 and 8. (XLSX)

S1 Raw images Author-provided full-field blot exports, exposure pages, lane/crop mappings, and repeat-level source images underlying Figs 7 and 8. The project archive did not contain the proprietary instrument-native acquisition files; this limitation is disclosed in the cover letter.
(PDF)

S2 File Core code and source data. Analysis scripts, figure-level numerical source data, WGCNA outputs other than the two large RDS matrices, corrected DEG and clinical-validation inputs, donor-level single-cell outputs, docking and molecular-dynamics summaries, quantitative in vitro source files, a SHA-256 manifest, and the lossless reassembly script.
(ZIP)

S3 File WGCNA maximum-probe RDS matrix, lossless binary part 1 of 4. (ZIP)

S4 File WGCNA maximum-probe RDS matrix, lossless binary part 2 of 4. (ZIP)

S5 File WGCNA maximum-probe RDS matrix, lossless binary part 3 of 4. (ZIP)

S6 File WGCNA maximum-probe RDS matrix, lossless binary part 4 of 4. (ZIP)

S7 File WGCNA mean-probe sensitivity RDS matrix, lossless binary part 1 of 4. (ZIP)

S8 File WGCNA mean-probe sensitivity RDS matrix, lossless binary part 2 of 4. (ZIP)

S9 File WGCNA mean-probe sensitivity RDS matrix, lossless binary part 3 of 4. (ZIP)

S10 File WGCNA mean-probe sensitivity RDS matrix, lossless binary part 4 of 4. (ZIP)
