# Supplementary material for: Multi-cohort transcriptomics and donor-aware single-cell analyses prioritize S100A8 within an emergency-myeloid, HLA-DR-low state in sepsis
Source: PLoS One. 2026 Oct 21;21(10):e0359578. doi: 10.1371/journal.pone.0359578 (PMC13686462; doi:10.1371/journal.pone.0359578)
Supplement: Supplementary file 1 [file pone.0359578.s001.pdf]

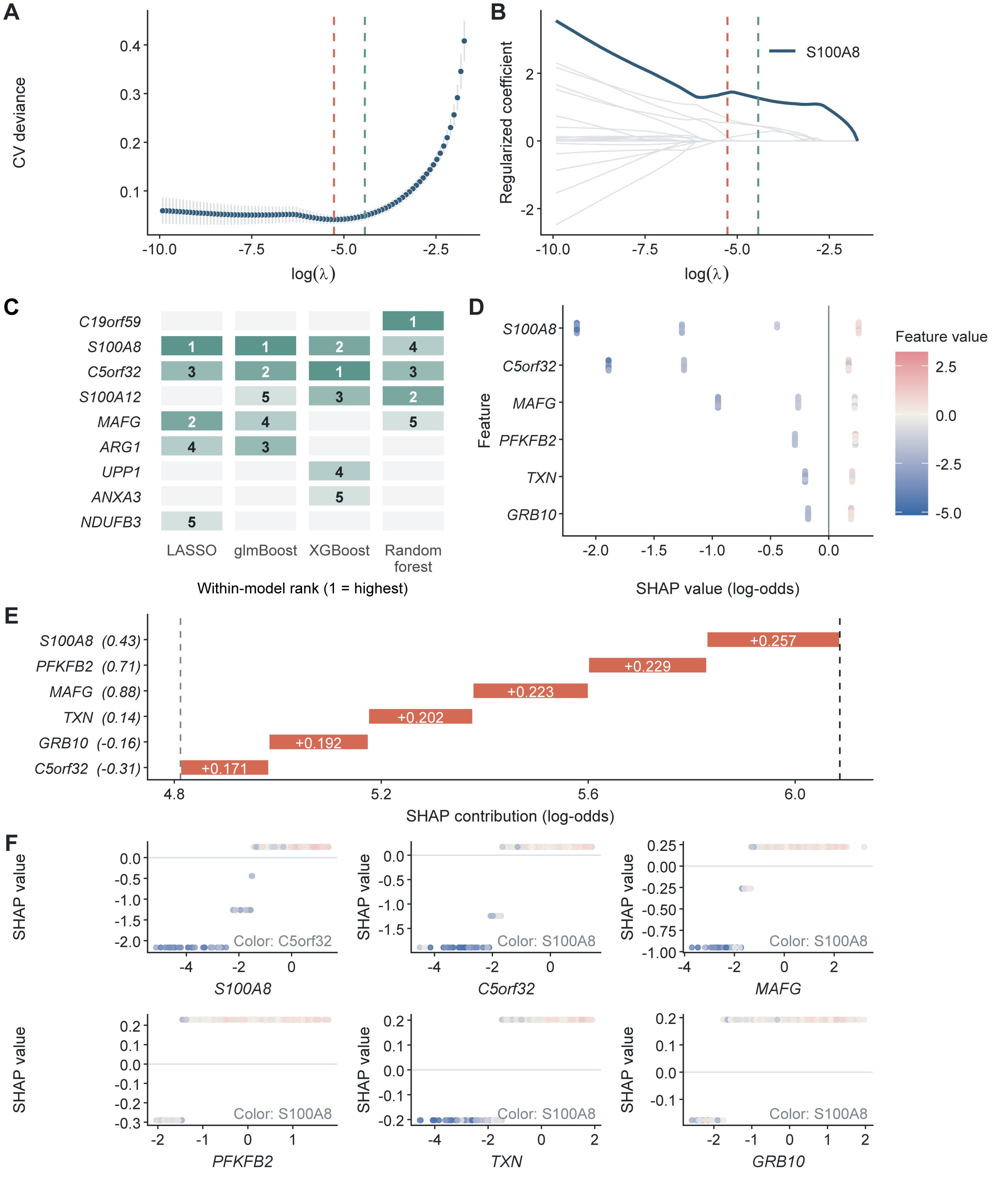

A
CV deviance
log(λ)
B
Regularized coefficient
S100A8
log(λ)
C
C19orf59
S100A8
C5orf32
S100A12
MAFG
ARG1
UPP1
ANXA3
NDUFB3
LASSO
glmBoost
XGBoost
Random forest
Within-model rank (1 = highest)
D
Feature
Feature value
SHAP value (log-odds)
E
S100A8 (0.43)
PFKFB2 (0.71)
MAFG (0.88)
TXN (0.14)
GRB10 (-0.16)
C5orf32 (-0.31)
+0.257
+0.229
+0.223
+0.202
+0.192
+0.171
SHAP contribution (log-odds)
F
SHAP value
Color: C5orf32
Color: S100A8
S100A8
C5orf32
MAFG
PFKFB2
TXN
GRB10
