# Supplementary material for: Multi-cohort transcriptomics and donor-aware single-cell analyses prioritize S100A8 within an emergency-myeloid, HLA-DR-low state in sepsis
Source: PLoS One. 2026 Oct 21;21(10):e0359578. doi: 10.1371/journal.pone.0359578 (PMC13686462; doi:10.1371/journal.pone.0359578)
Supplement: Supplementary file 2 [file pone.0359578.s002.pdf]

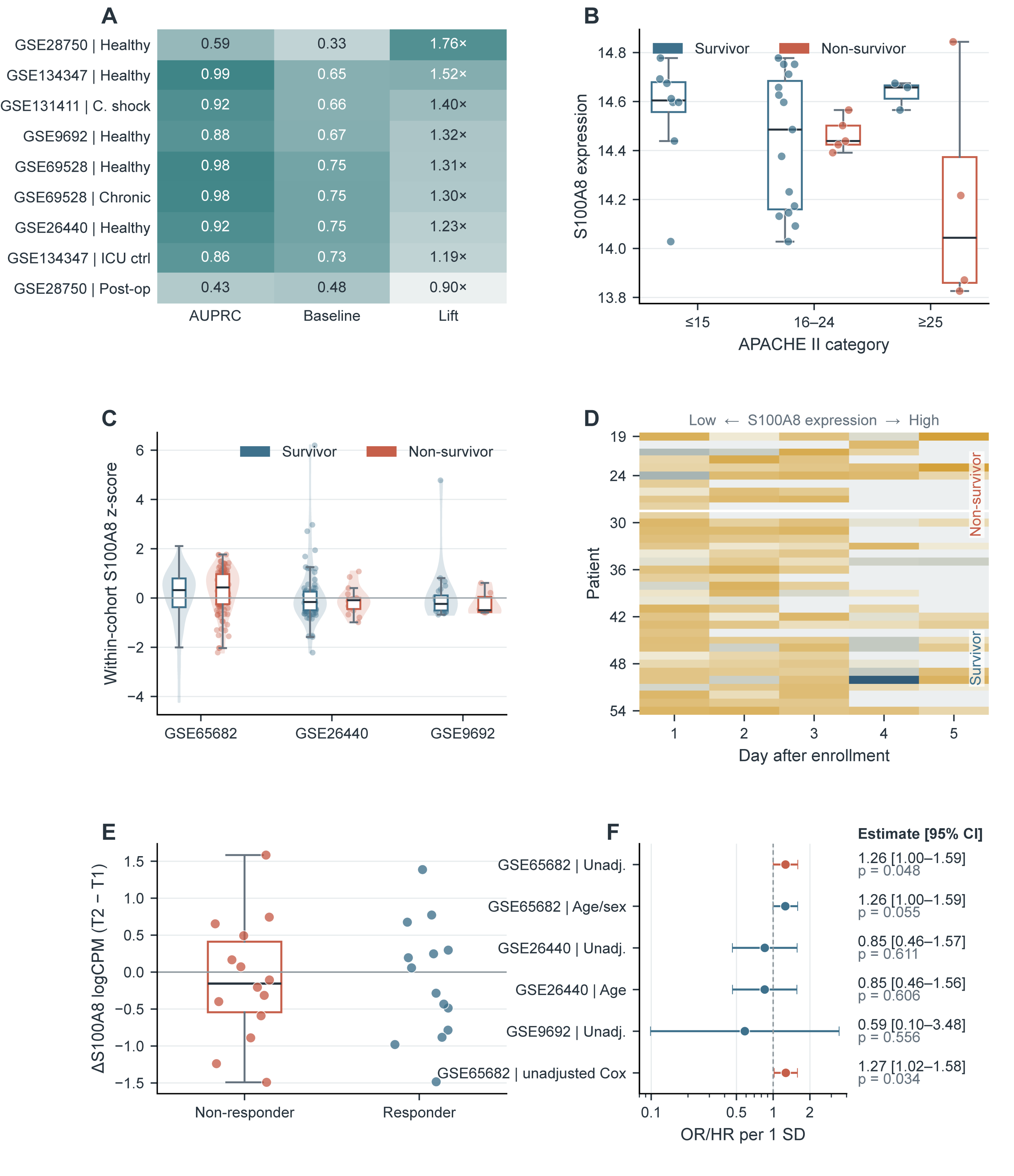

	AUPRC	Baseline	Lift
GSE28750 | Healthy	0.59	0.33	1.76×
GSE134347 | Healthy	0.99	0.65	1.52×
GSE131411 | C. shock	0.92	0.66	1.40×
GSE9692 | Healthy	0.88	0.67	1.32×
GSE69528 | Healthy	0.98	0.75	1.31×
GSE69528 | Chronic	0.98	0.75	1.30×
GSE26440 | Healthy	0.92	0.75	1.23×
GSE134347 | ICU ctrl	0.86	0.73	1.19×
GSE28750 | Post-op	0.43	0.48	0.90×
