# Supplementary material for: Multi-cohort transcriptomics and donor-aware single-cell analyses prioritize S100A8 within an emergency-myeloid, HLA-DR-low state in sepsis
Source: PLoS One. 2026 Oct 21;21(10):e0359578. doi: 10.1371/journal.pone.0359578 (PMC13686462; doi:10.1371/journal.pone.0359578)
Supplement: Supplementary file 3 [file pone.0359578.s003.pdf]

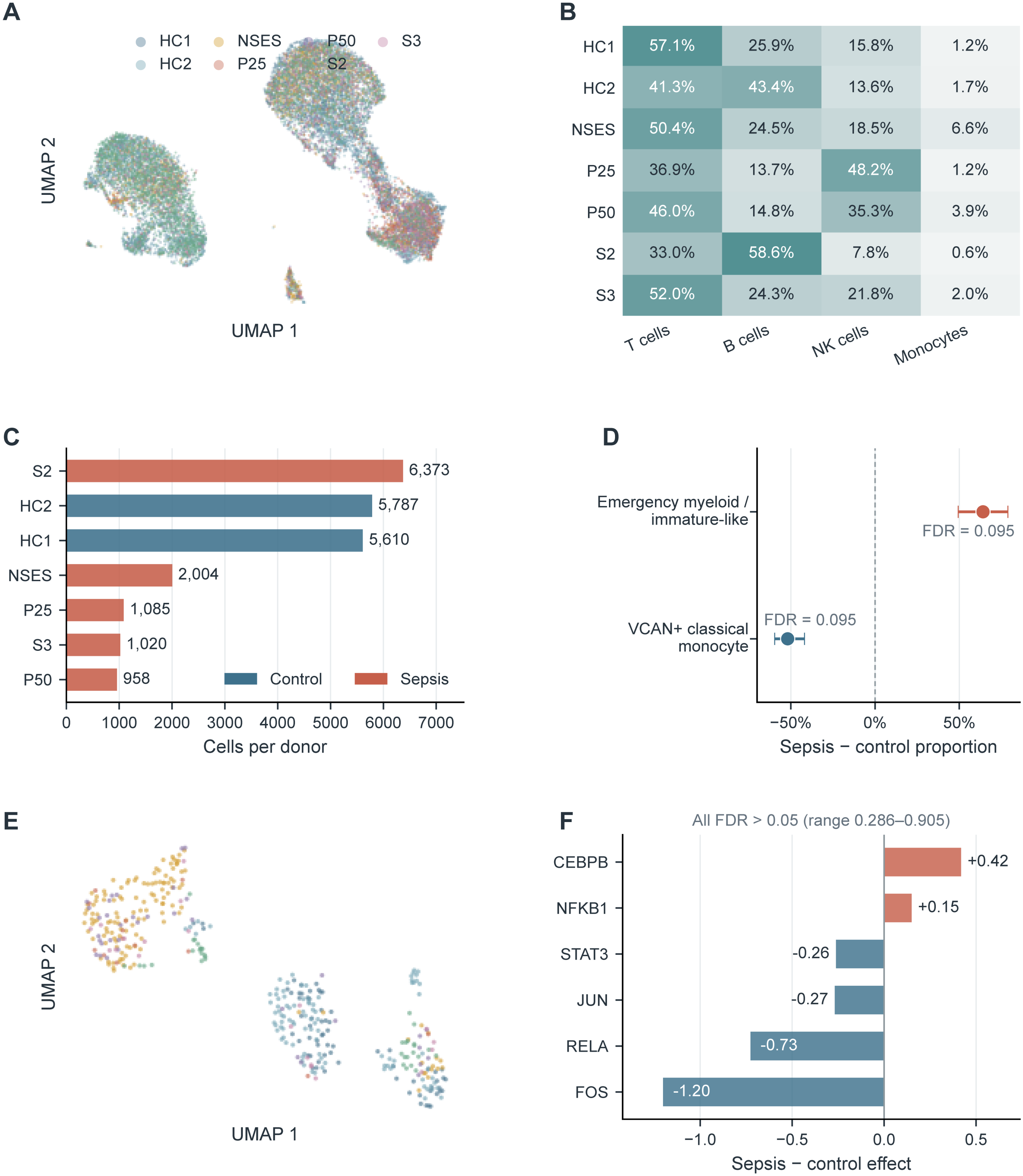
A
HC1
HC2
NSES
P25
P50
S2
S3
UMAP 2
UMAP 1
B
HC1 57.1% 25.9% 15.8% 1.2%
HC2 41.3% 43.4% 13.6% 1.7%
NSES 50.4% 24.5% 18.5% 6.6%
P25 36.9% 13.7% 48.2% 1.2%
P50 46.0% 14.8% 35.3% 3.9%
S2 33.0% 58.6% 7.8% 0.6%
S3 52.0% 24.3% 21.8% 2.0%
T cells
B cells
NK cells
Monocytes
C
S2 6,373
HC2 5,787
HC1 5,610
NSES 2,004
P25 1,085
S3 1,020
P50 958
Control
Sepsis
0
1000
2000
3000
4000
5000
6000
7000
Cells per donor
D
Emergency myeloid / immature-like
FDR = 0.095
VCAN+ classical monocyte
FDR = 0.095
−50%
0%
50%
Sepsis − control proportion
E
UMAP 2
UMAP 1
F
All FDR > 0.05 (range 0.286–0.905)
CEBPB +0.42
NFKB1 +0.15
STAT3 -0.26
JUN -0.27
RELA -0.73
FOS -1.20
−1.0
−0.5
0.0
0.5
Sepsis − control effect
