# Supplementary material for: Multi-cohort transcriptomics and donor-aware single-cell analyses prioritize S100A8 within an emergency-myeloid, HLA-DR-low state in sepsis
Source: PLoS One. 2026 Oct 21;21(10):e0359578. doi: 10.1371/journal.pone.0359578 (PMC13686462; doi:10.1371/journal.pone.0359578)
Supplement: Supplementary file 4 [file pone.0359578.s004.pdf]

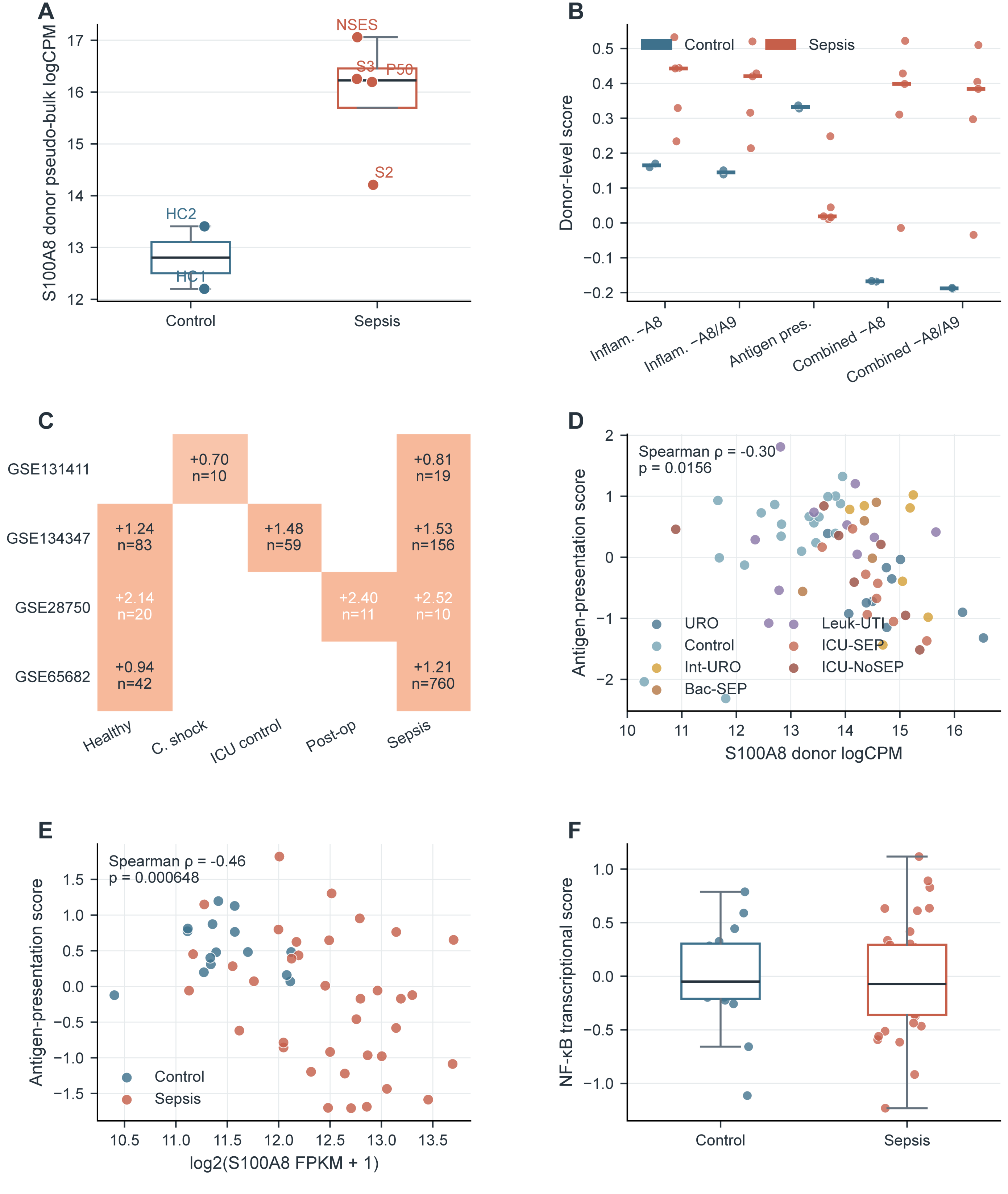

A
S100A8 donor pseudo-bulk logCPM
NSES
S3
P50
S2
HC2
HC1
Control
Sepsis
B
Control
Sepsis
Donor-level score
Inflam. −A8
Inflam. −A8/A9
Antigen pres.
Combined −A8
Combined −A8/A9
C
GSE131411
GSE134347
GSE28750
GSE65682
+0.70 n=10
+0.81 n=19
+1.24 n=83
+1.48 n=59
+1.53 n=156
+2.14 n=20
+2.40 n=11
+2.52 n=10
+0.94 n=42
+1.21 n=760
Healthy
C. shock
ICU control
Post-op
Sepsis
D
Spearman ρ = -0.30
p = 0.0156
Antigen-presentation score
URO
Control
Int-URO
Bac-SEP
Leuk-UTI
ICU-SEP
ICU-NoSEP
S100A8 donor logCPM
E
Spearman ρ = -0.46
p = 0.000648
Antigen-presentation score
Control
Sepsis
log2(S100A8 FPKM + 1)
F
NF-κB transcriptional score
Control
Sepsis
