# Supplementary material for: Multi-cohort transcriptomics and donor-aware single-cell analyses prioritize S100A8 within an emergency-myeloid, HLA-DR-low state in sepsis
Source: PLoS One. 2026 Oct 21;21(10):e0359578. doi: 10.1371/journal.pone.0359578 (PMC13686462; doi:10.1371/journal.pone.0359578)
Supplement: Supplementary file 5 [file pone.0359578.s005.pdf]

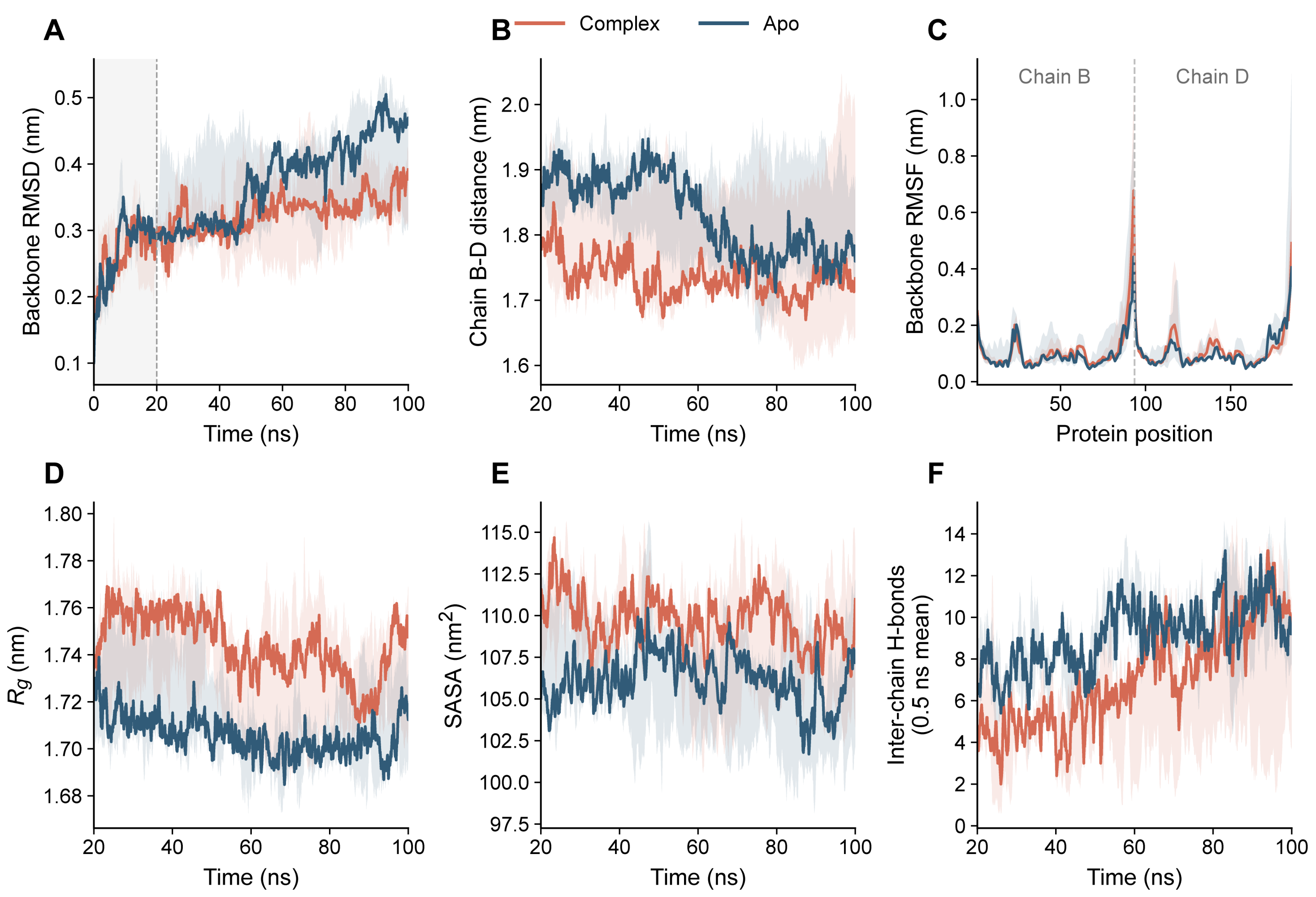

A
B
C
D
E
F
Complex
Apo
Backbone RMSD (nm)
Time (ns)
Chain B-D distance (nm)
Backbone RMSF (nm)
Chain B
Chain D
Protein position
R_g (nm)
SASA (nm^2)
Inter-chain H-bonds
(0.5 ns mean)
