# Supplementary material for: Multi-cohort transcriptomics and donor-aware single-cell analyses prioritize S100A8 within an emergency-myeloid, HLA-DR-low state in sepsis
Source: PLoS One. 2026 Oct 21;21(10):e0359578. doi: 10.1371/journal.pone.0359578 (PMC13686462; doi:10.1371/journal.pone.0359578)
Supplement: Supplementary file 6 [file pone.0359578.s006.pdf]

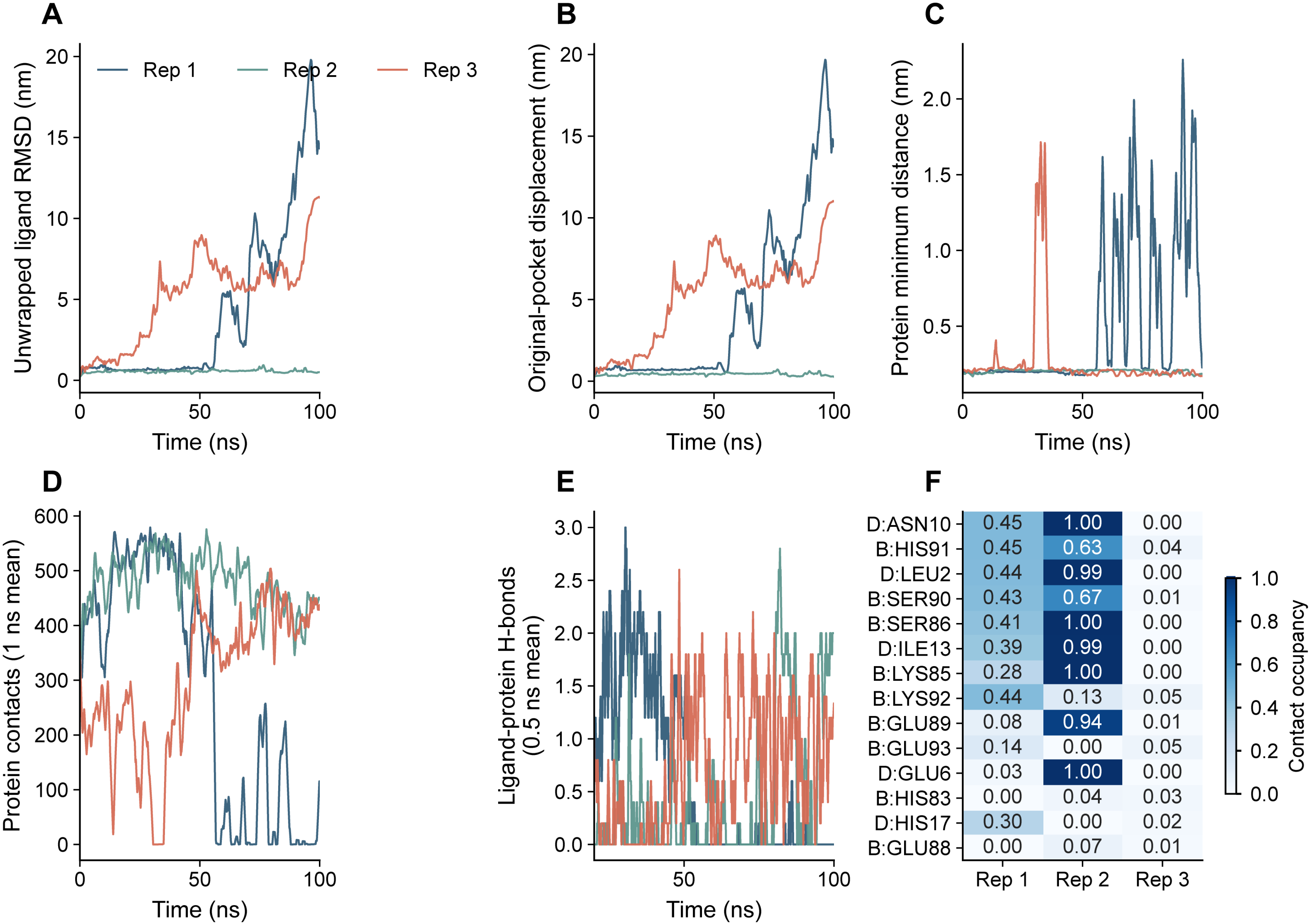
A
B
C
D
E
F
Rep 1
Rep 2
Rep 3
Unwrapped ligand RMSD (nm)
Original-pocket displacement (nm)
Protein minimum distance (nm)
Protein contacts (1 ns mean)
Ligand-protein H-bonds (0.5 ns mean)
Time (ns)
0
50
100
5
10
15
20
0.5
1.0
1.5
2.0
200
300
400
500
600
2.5
3.0
0.0
D:ASN10 0.45 1.00 0.00
B:HIS91 0.45 0.63 0.04
D:LEU2 0.44 0.99 0.00
B:SER90 0.43 0.67 0.01
B:SER86 0.41 1.00 0.00
D:ILE13 0.39 0.99 0.00
B:LYS85 0.28 1.00 0.00
B:LYS92 0.44 0.13 0.05
B:GLU89 0.08 0.94 0.01
B:GLU93 0.14 0.00 0.05
D:GLU6 0.03 1.00 0.00
B:HIS83 0.00 0.04 0.03
D:HIS17 0.30 0.00 0.02
B:GLU88 0.00 0.07 0.01
Contact occupancy
0.0
0.2
0.4
0.6
0.8
1.0
