# Supplementary material for: Multi-cohort transcriptomics and donor-aware single-cell analyses prioritize S100A8 within an emergency-myeloid, HLA-DR-low state in sepsis
Source: PLoS One. 2026 Oct 21;21(10):e0359578. doi: 10.1371/journal.pone.0359578 (PMC13686462; doi:10.1371/journal.pone.0359578)
Supplement: Supplementary file 7 [file pone.0359578.s007.pdf]

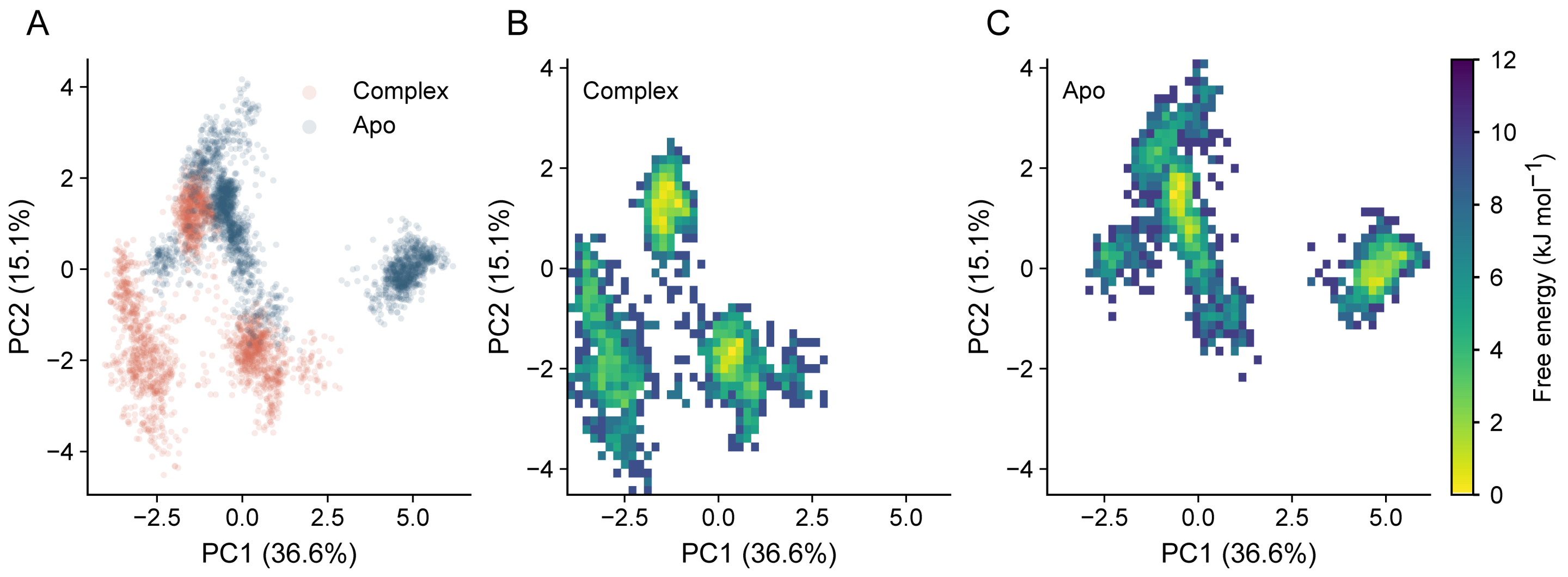

A
B
C
Complex
Apo
Complex
Apo
PC1 (36.6%)
PC2 (15.1%)
−2.5
0.0
2.5
5.0
4
2
0
−2
−4
0
2
4
6
8
10
12
Free energy (kJ mol^{-1})
