# Supplementary material for: Multi-cohort transcriptomics and donor-aware single-cell analyses prioritize S100A8 within an emergency-myeloid, HLA-DR-low state in sepsis
Source: PLoS One. 2026 Oct 21;21(10):e0359578. doi: 10.1371/journal.pone.0359578 (PMC13686462; doi:10.1371/journal.pone.0359578)
Supplement: Supplementary file 8 [file pone.0359578.s008.pdf]

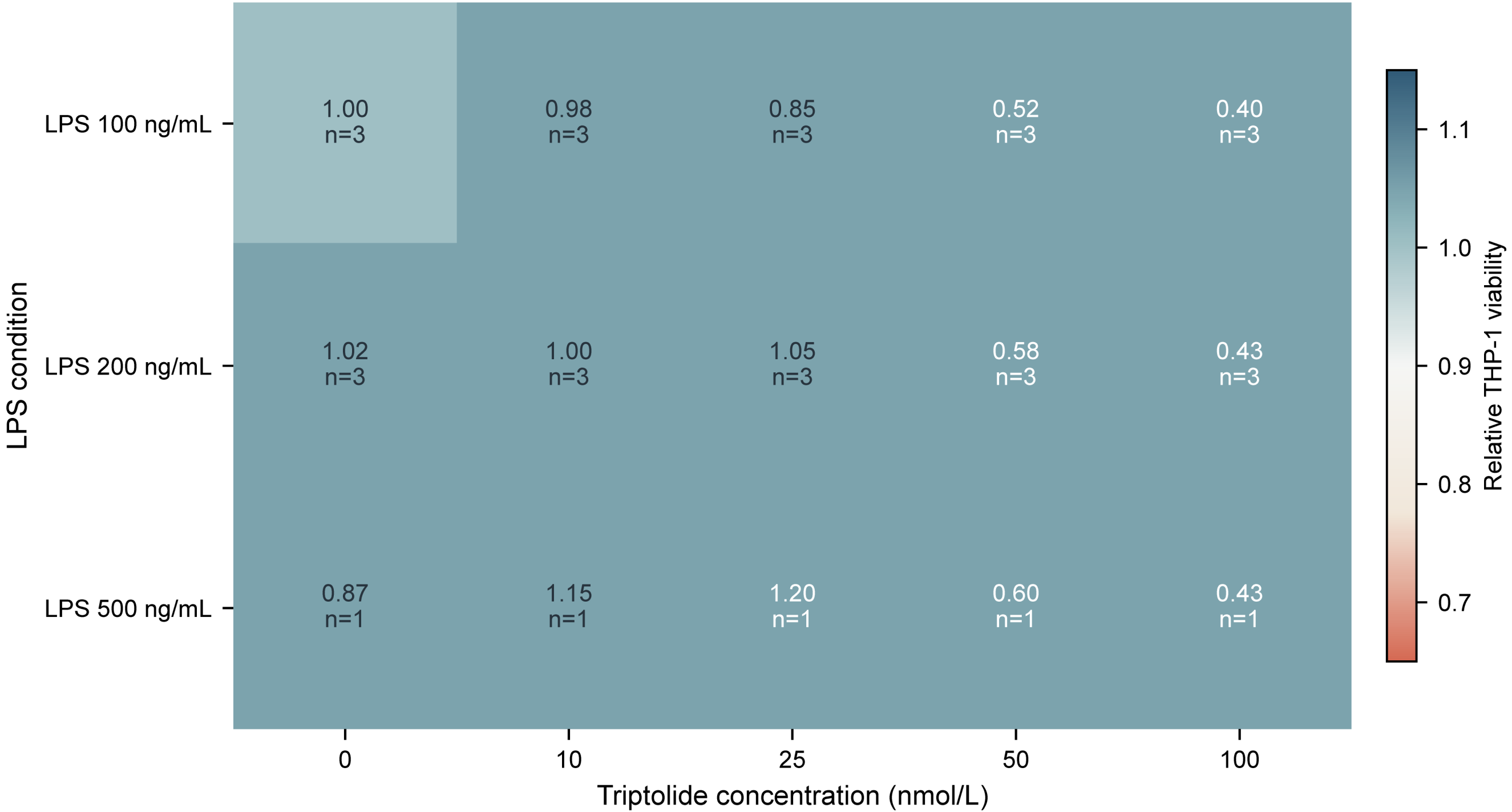
LPS condition
LPS 100 ng/mL
LPS 200 ng/mL
LPS 500 ng/mL
1.00 n=3
0.98 n=3
0.85 n=3
0.52 n=3
0.40 n=3
1.02 n=3
1.00 n=3
1.05 n=3
0.58 n=3
0.43 n=3
0.87 n=1
1.15 n=1
1.20 n=1
0.60 n=1
0.43 n=1
0
10
25
50
100
Triptolide concentration (nmol/L)
Relative THP-1 viability
1.1
1.0
0.9
0.8
0.7
