# Supplementary material for: Multi-cohort transcriptomics and donor-aware single-cell analyses prioritize S100A8 within an emergency-myeloid, HLA-DR-low state in sepsis
Source: PLoS One. 2026 Oct 21;21(10):e0359578. doi: 10.1371/journal.pone.0359578 (PMC13686462; doi:10.1371/journal.pone.0359578)
Supplement: Supplementary file 9 [file pone.0359578.s016.pdf]

S1 raw images

Full-field blot images and crop mapping for revised Figs 7 and 8

Final panel mapping

PDF pages	Final panels	Source content
Pages 2-7	Fig 7B-C	Three-repeat Western blot full fields and crop maps: Control, LPS, and LPS+TPL.
Pages 8-11	Fig 7D-E	Three-repeat CETSA full fields plus the temperature-series crop map.
Pages 12-17	Fig 8A-C	Eight-condition siNC/siS100A8 blots used for NF-kB and S100A8 analyses.
Pages 18-20	Fig 8D-G	Four-condition siNC/siS100A8 blots used for CD74, HLA-DRalpha, and S100A8 analyses.

Compilation and integrity notes

- Sample loading order, target identity, molecular-weight labels, and repeat identity are retained from the author-supplied source pages.
- The image/data regions are reproduced without brightness, contrast, gamma, or background adjustment. Only the cover and page headers were added for final-panel traceability.
- Separate target rows may originate from separate membrane exposures, as indicated by the source layouts. No unused lanes were identified in the supplied layouts; therefore no lanes are marked with an X.
- The audited project archive contained full-field image exports, exposure pages, and crop mappings. Proprietary instrument-native acquisition files were not present; the authors should retain and provide those files if available and requested by the journal.
- Fig 8H is a statistical dependency summary and therefore has no corresponding blot image in this file.

Prepared for the PLOS ONE revision of manuscript PONE-D-26-24904. Raw-image pages follow unchanged apart from corrected page headers identifying their final figure-panel destinations.

Western blot - full imaging field - repeat 1

Control | LPS | LPS+TPL; S100A8, total NF-kB, p-NF-kB, and beta-actin. Final mapping: Fig 7B-C.

WB repeat 1

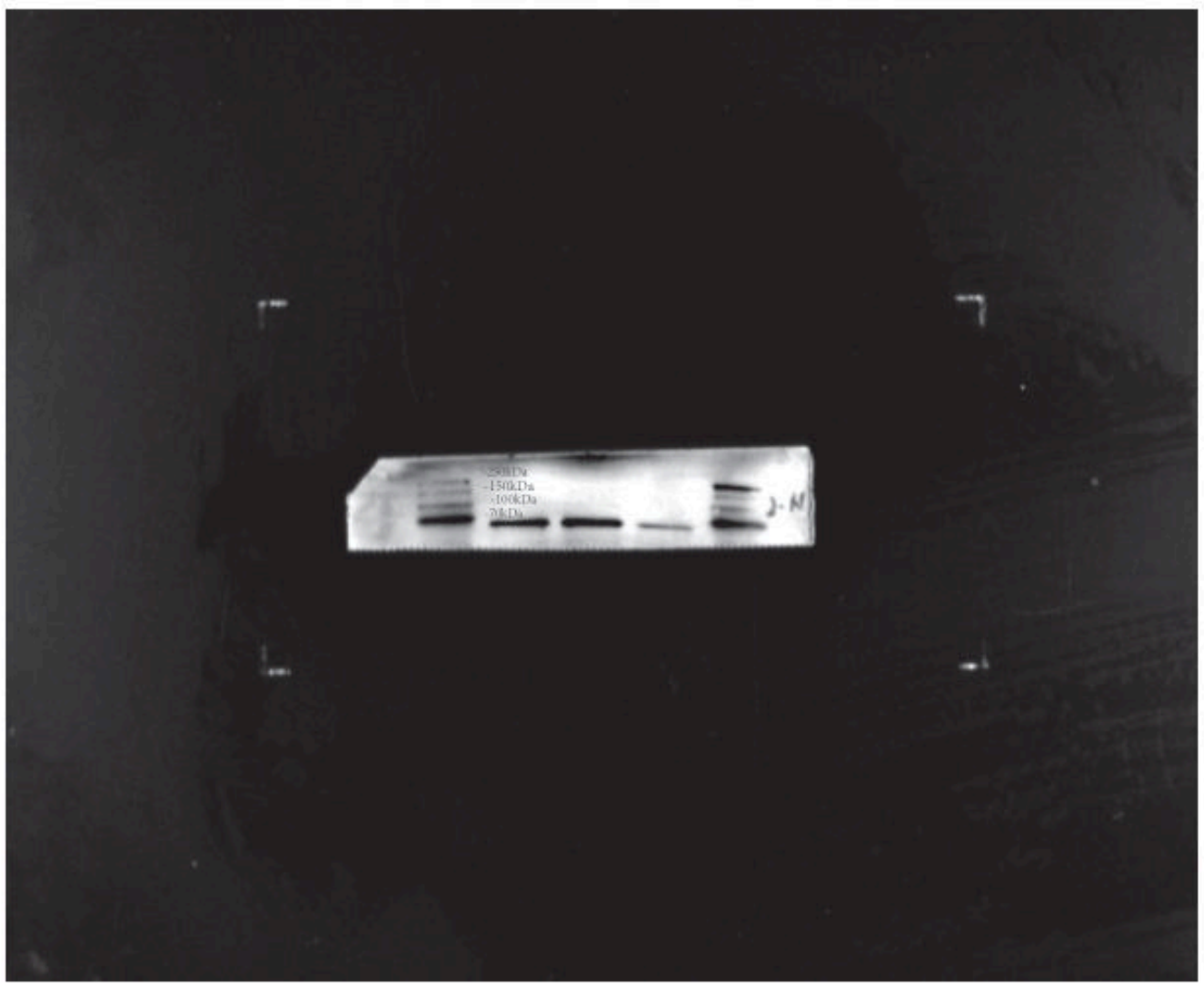

NF-κB 70kDa

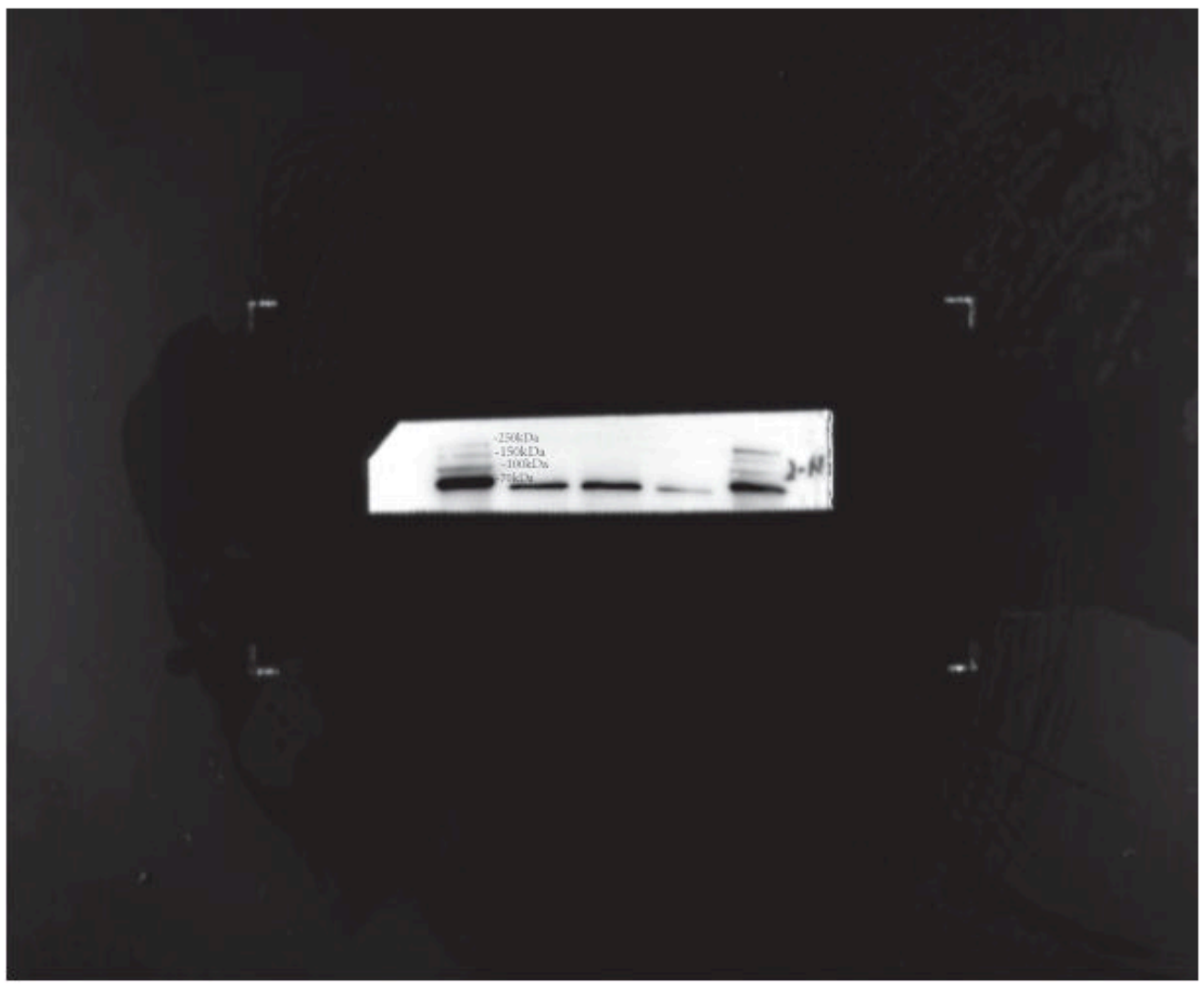

P-NF-κB 70kDa

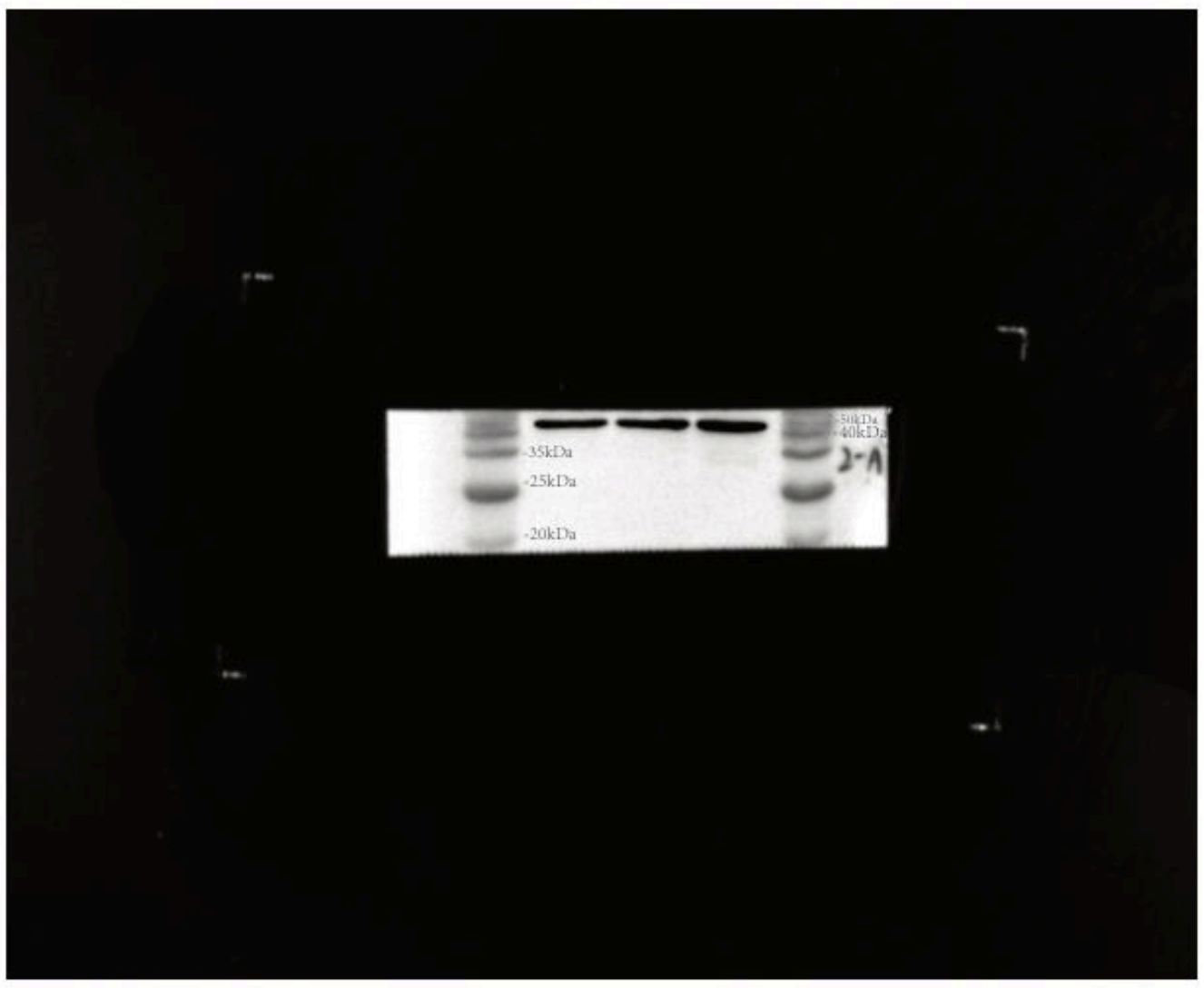

β-actin 42kDa

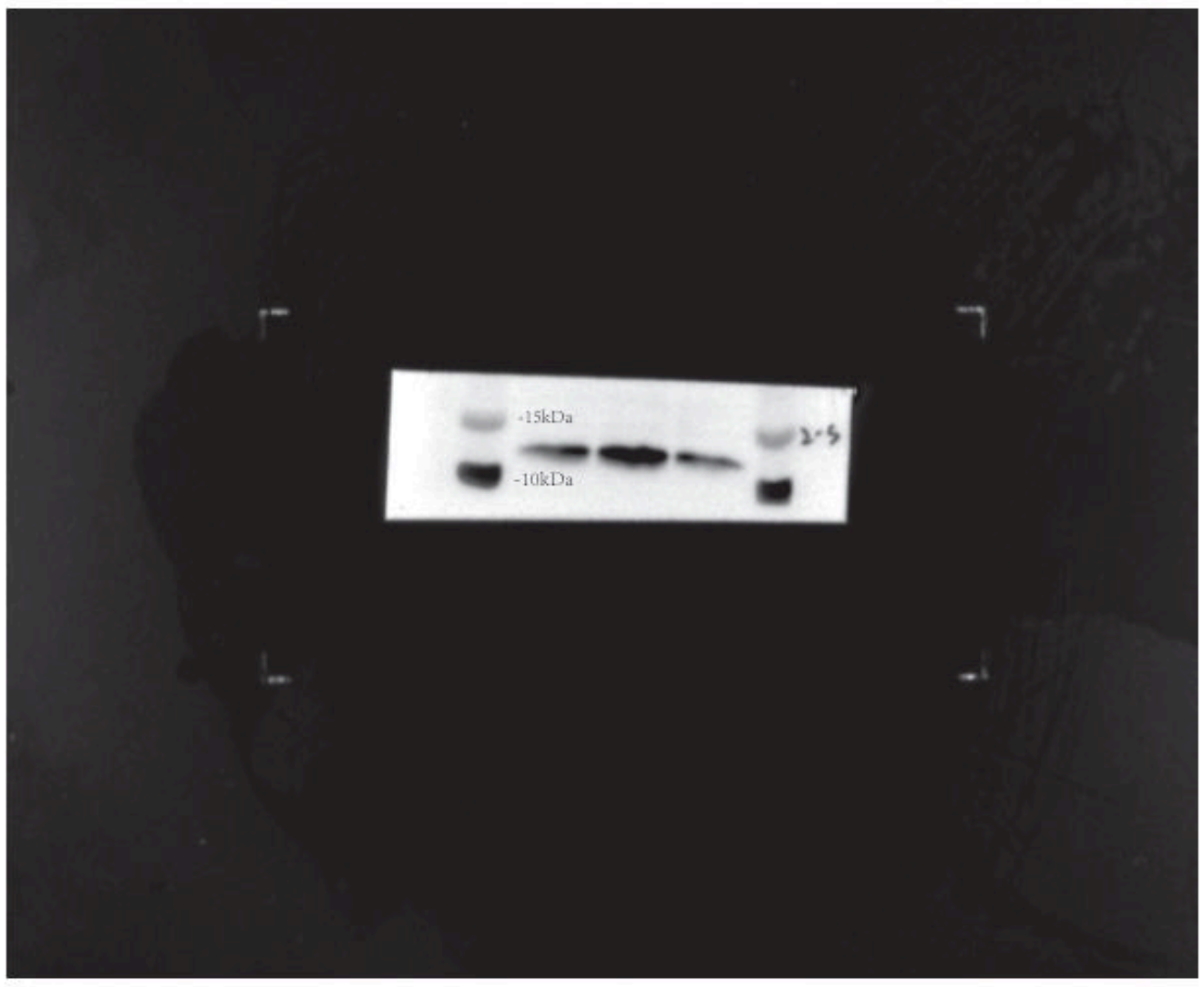

S100A8 11kDa

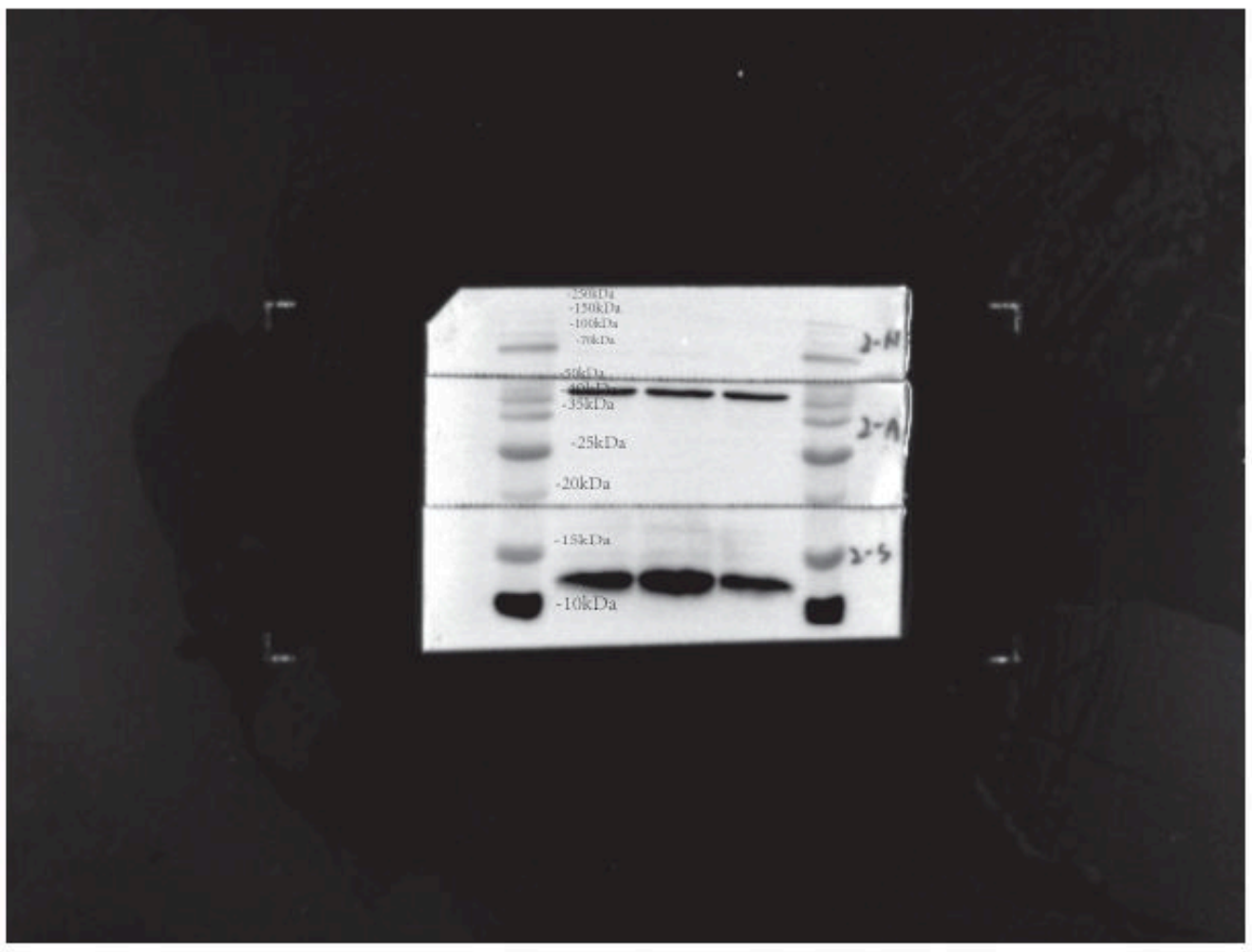

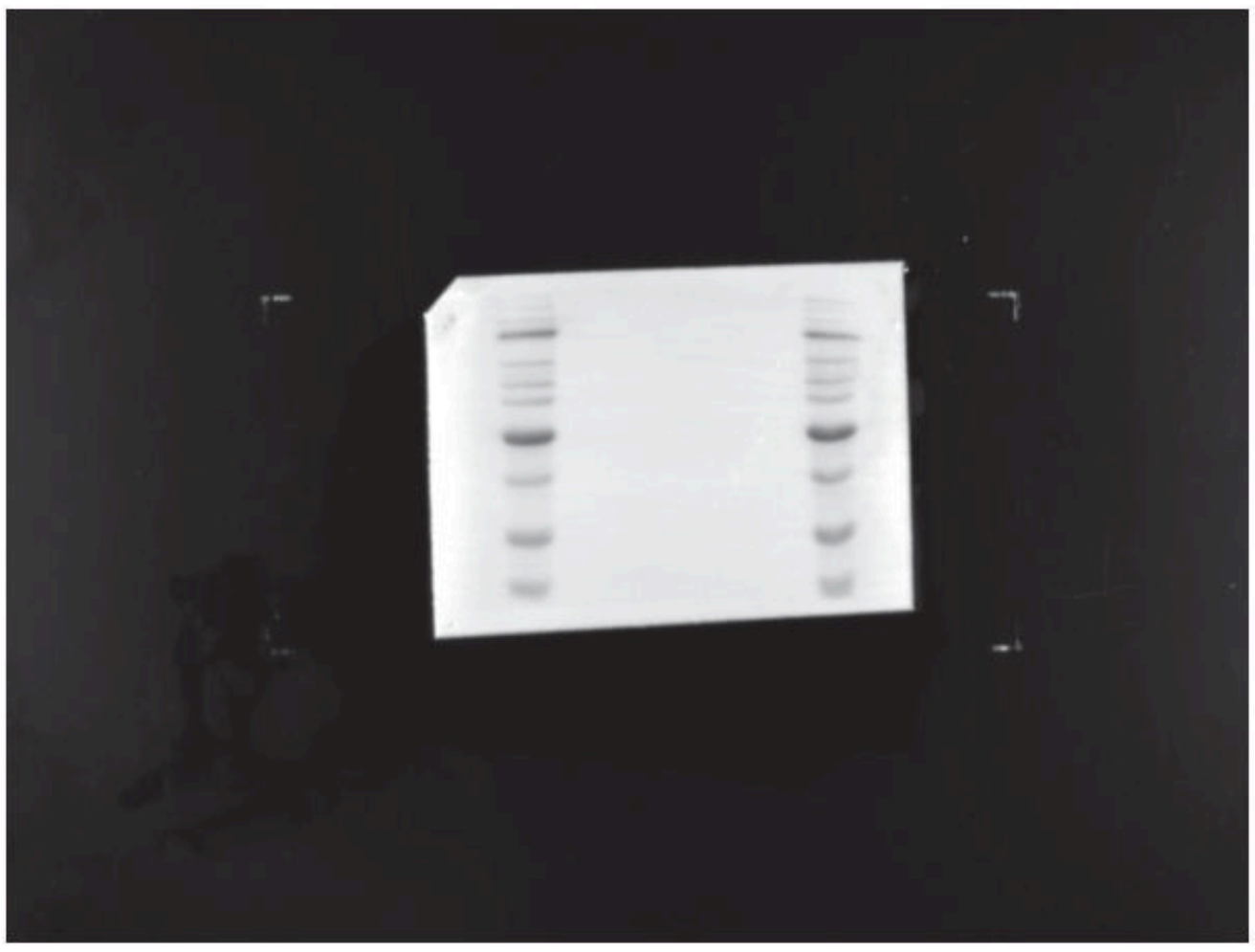

Source: Figure S4.pdf, original page 1. No brightness/contrast/gamma adjustment applied during compilation.

Western blot - full imaging field - repeat 2

Control | LPS | LPS+TPL; S100A8, total NF-kB, p-NF-kB, and beta-actin. Final mapping: Fig 7B-C.

repeat 2

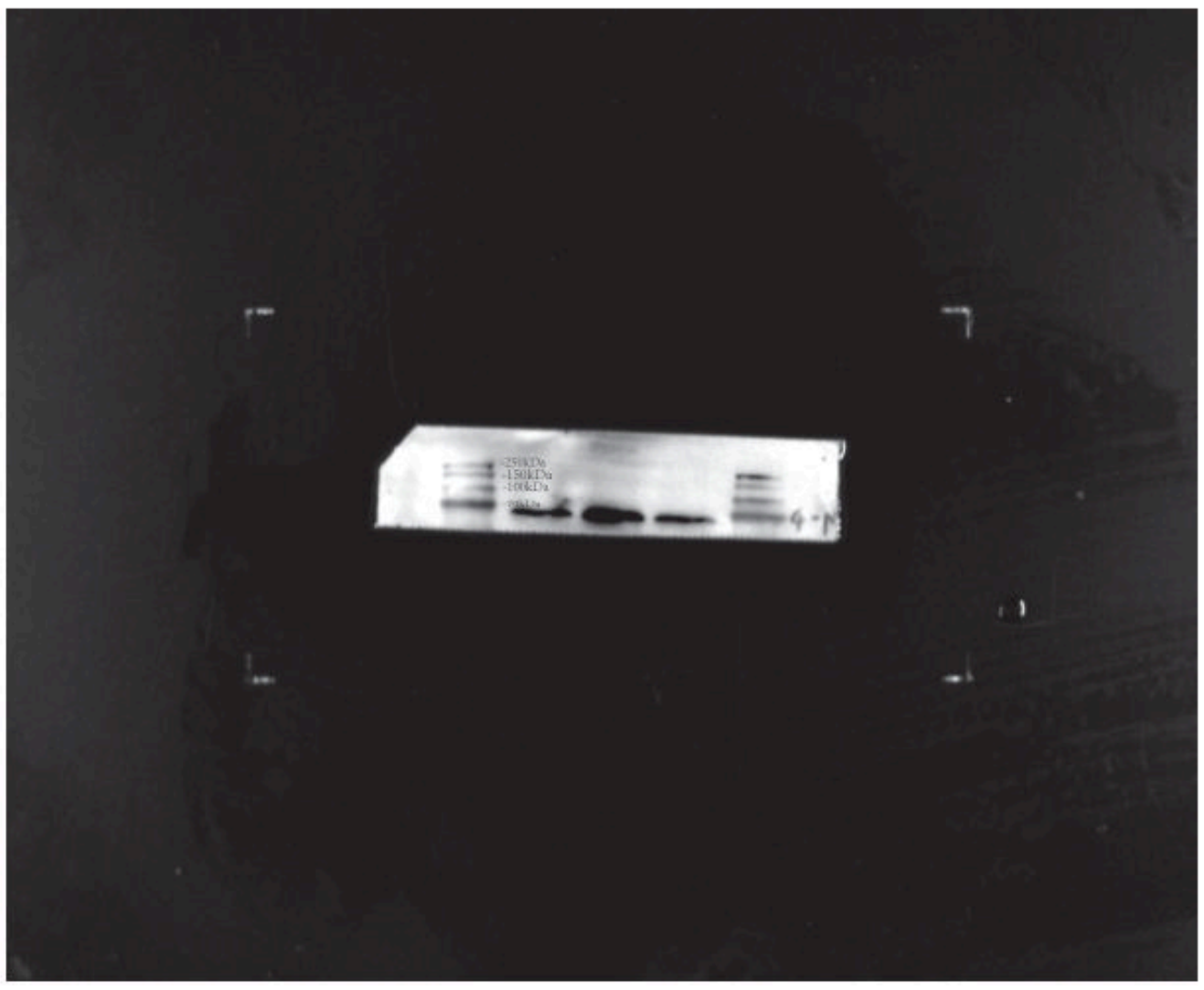

NF-κB 70kDa

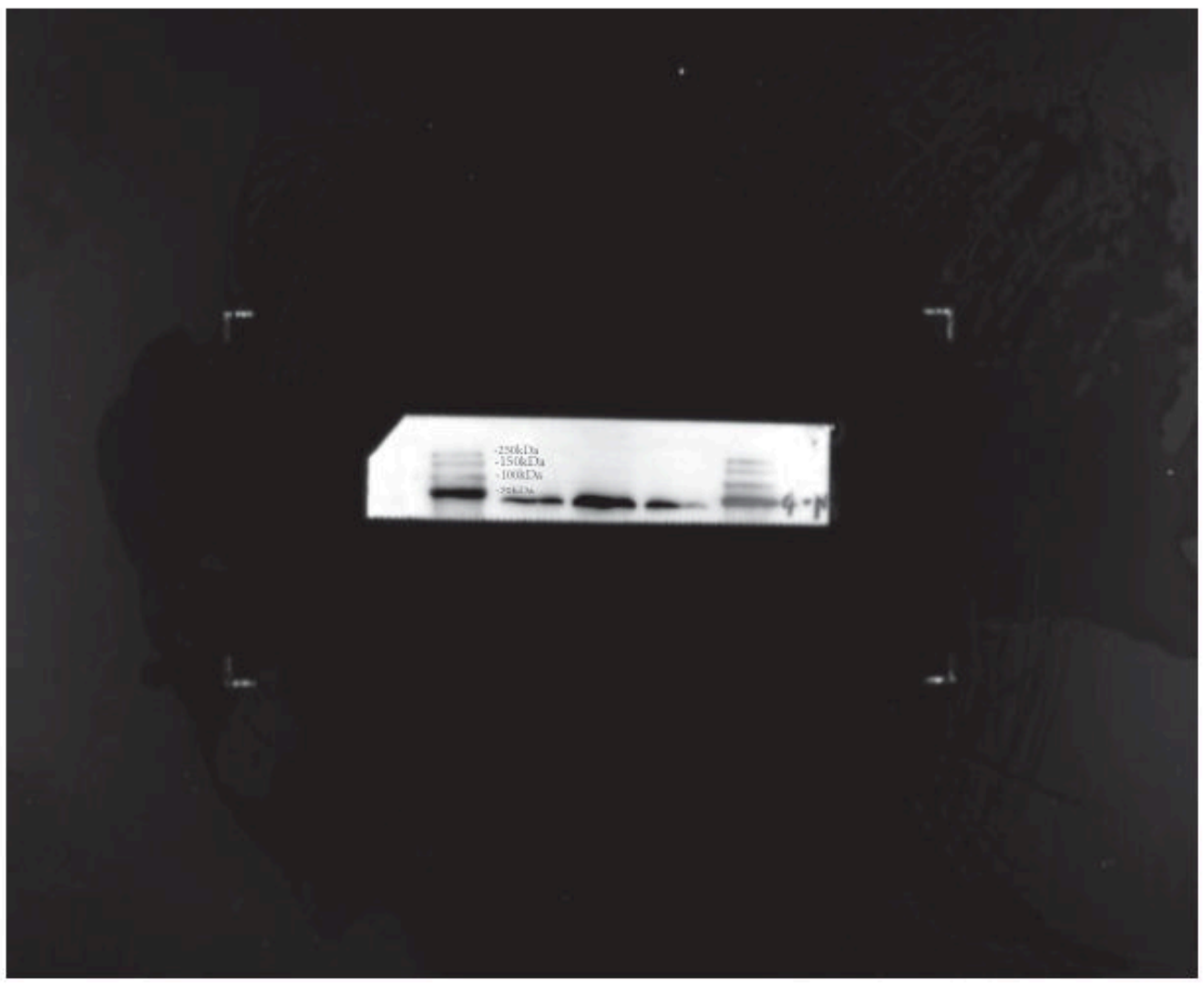

P-NF-κB 70kDa

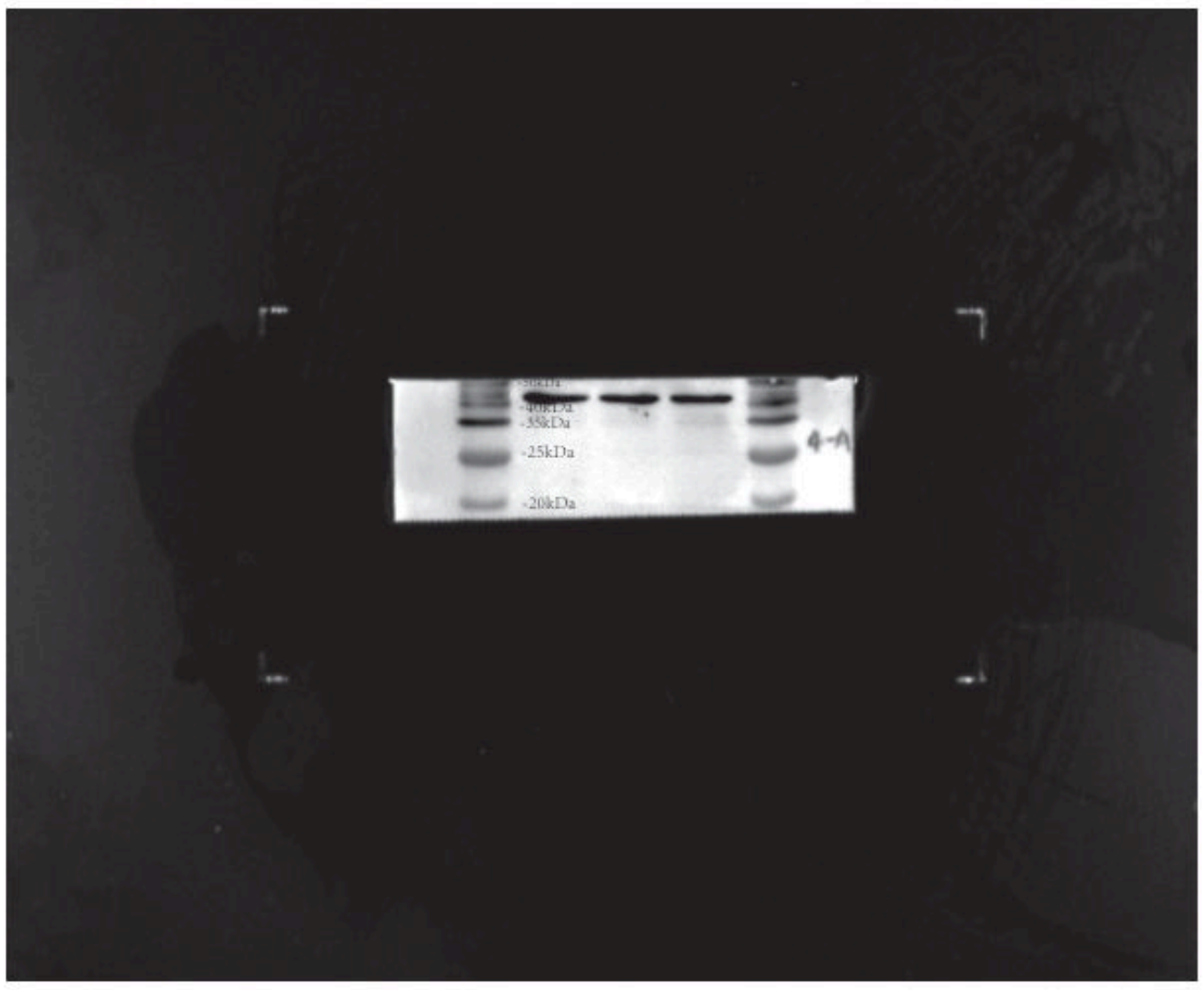

β-actin 42kDa

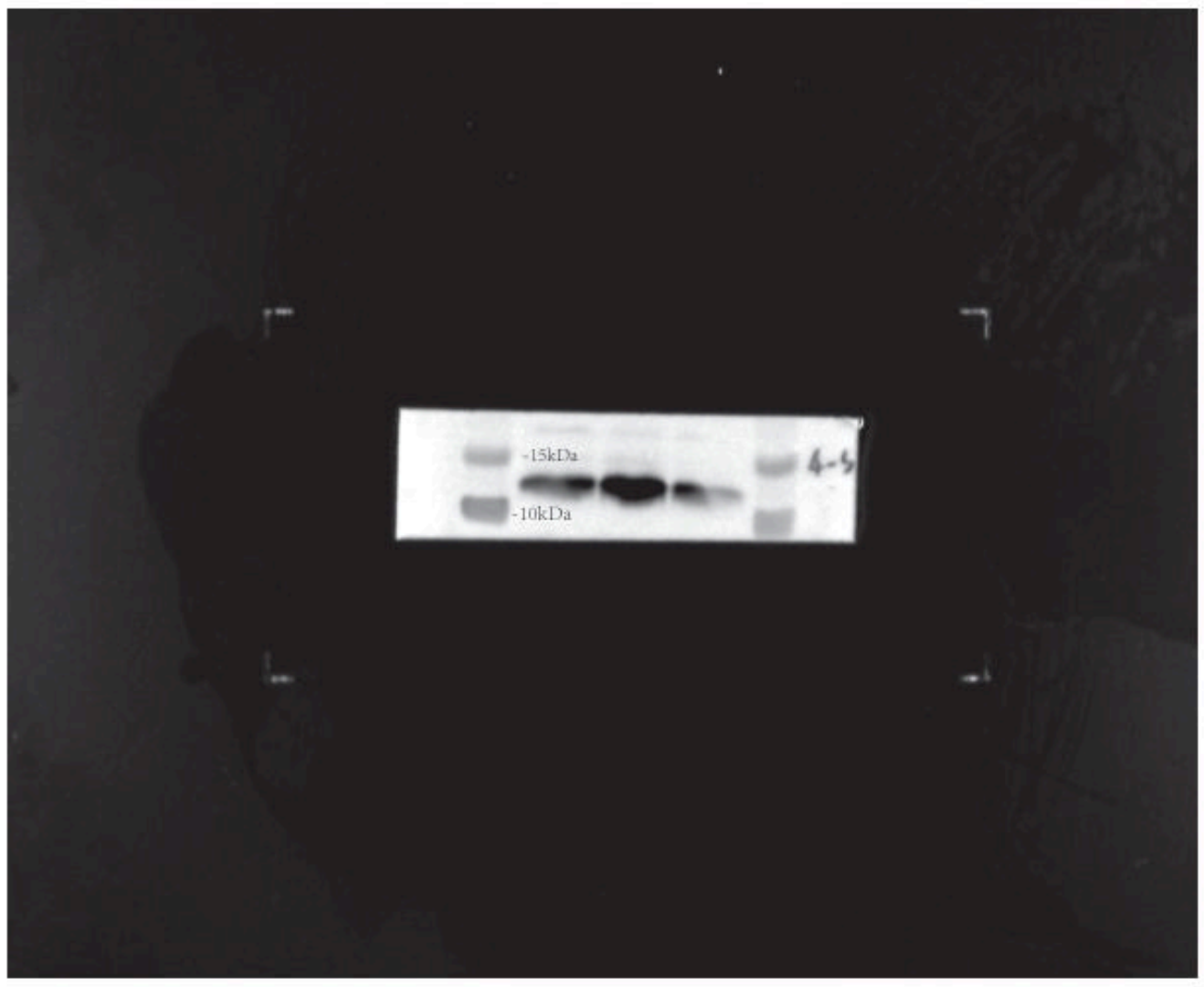

S100A8 11kDa

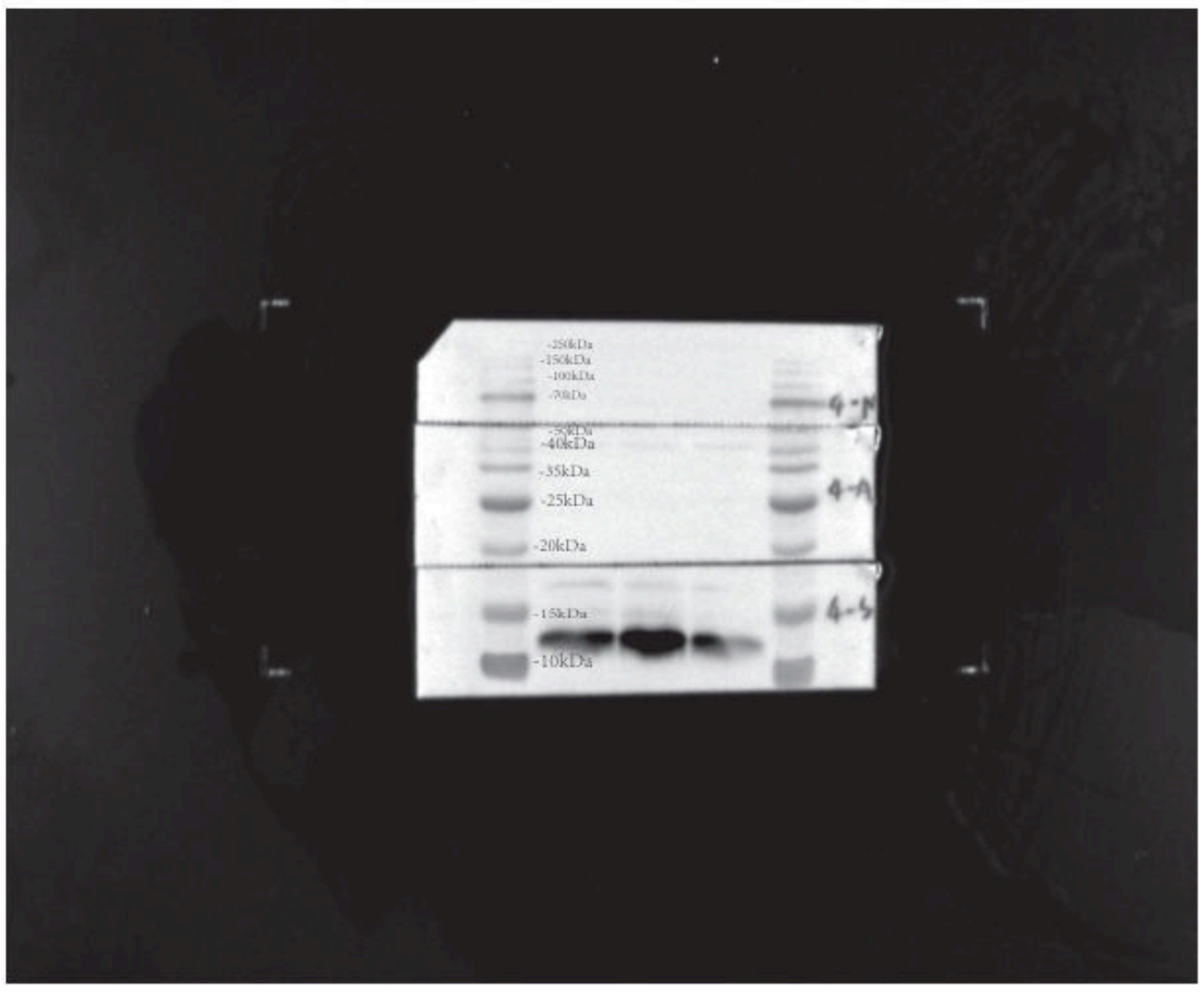

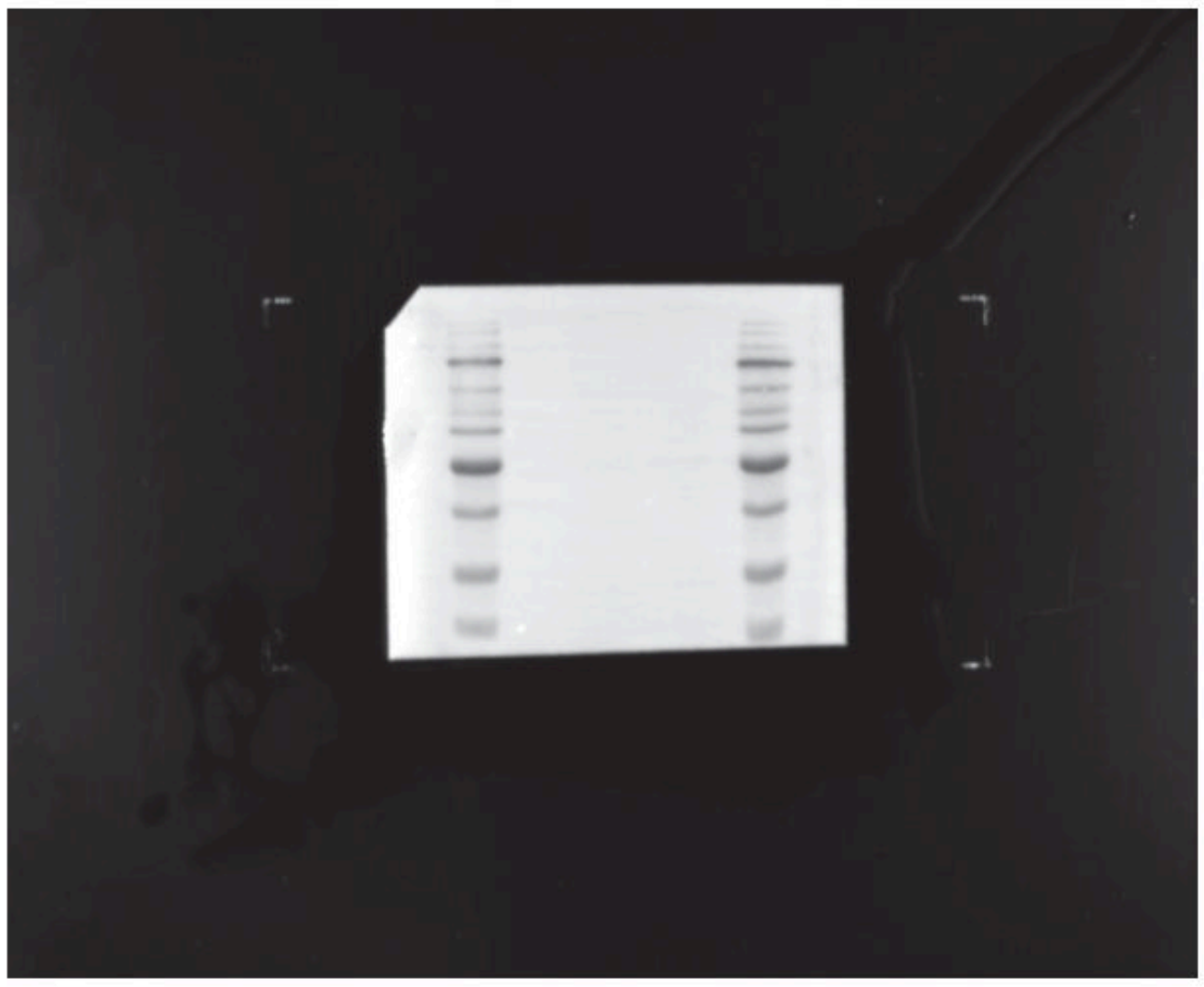

Source: Figure S4.pdf, original page 2. No brightness/contrast/gamma adjustment applied during compilation.

Western blot - full imaging field - repeat 3

Control | LPS | LPS+TPL; S100A8, total NF-kB, p-NF-kB, and beta-actin. Final mapping: Fig 7B-C.

repeat 3

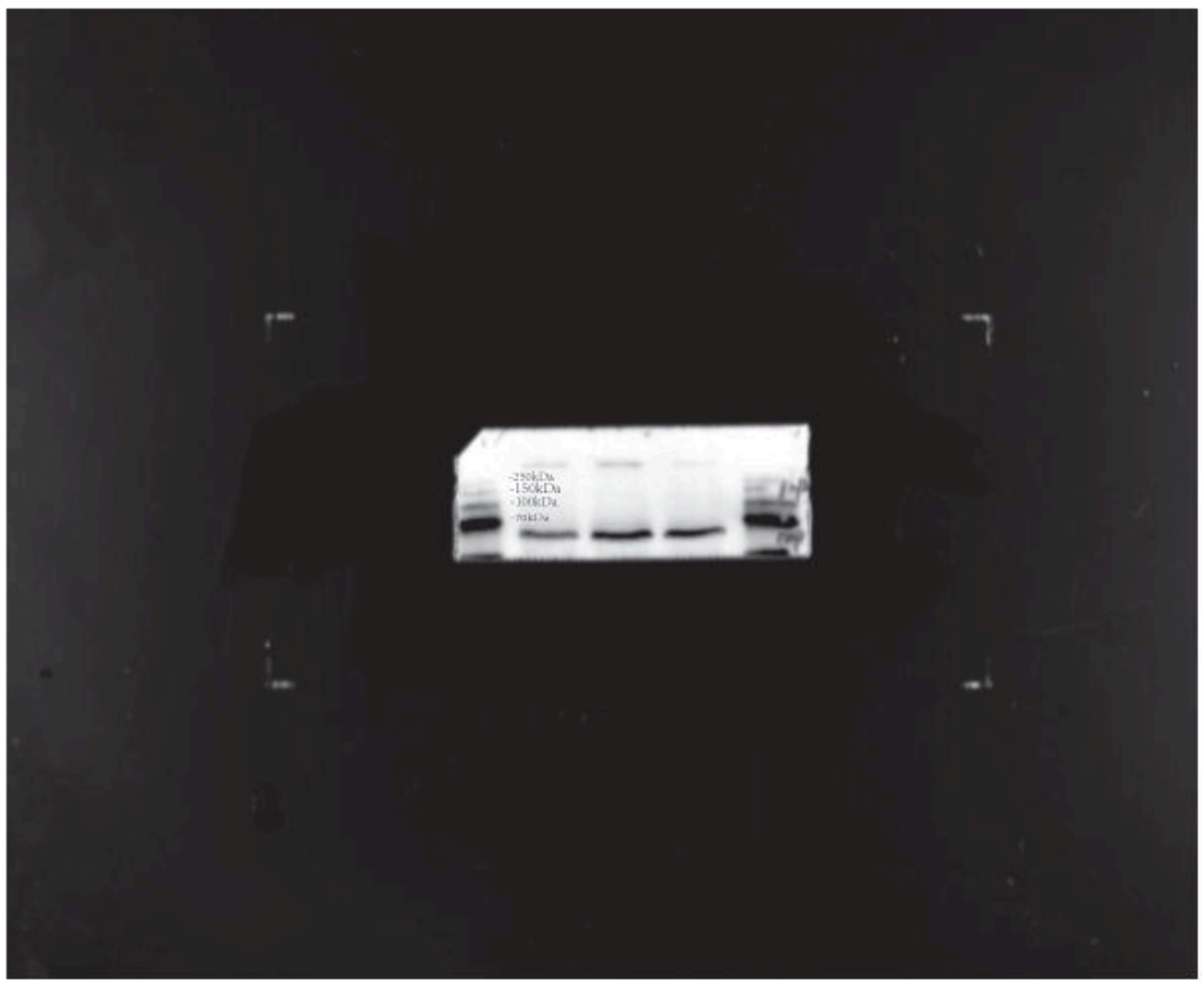
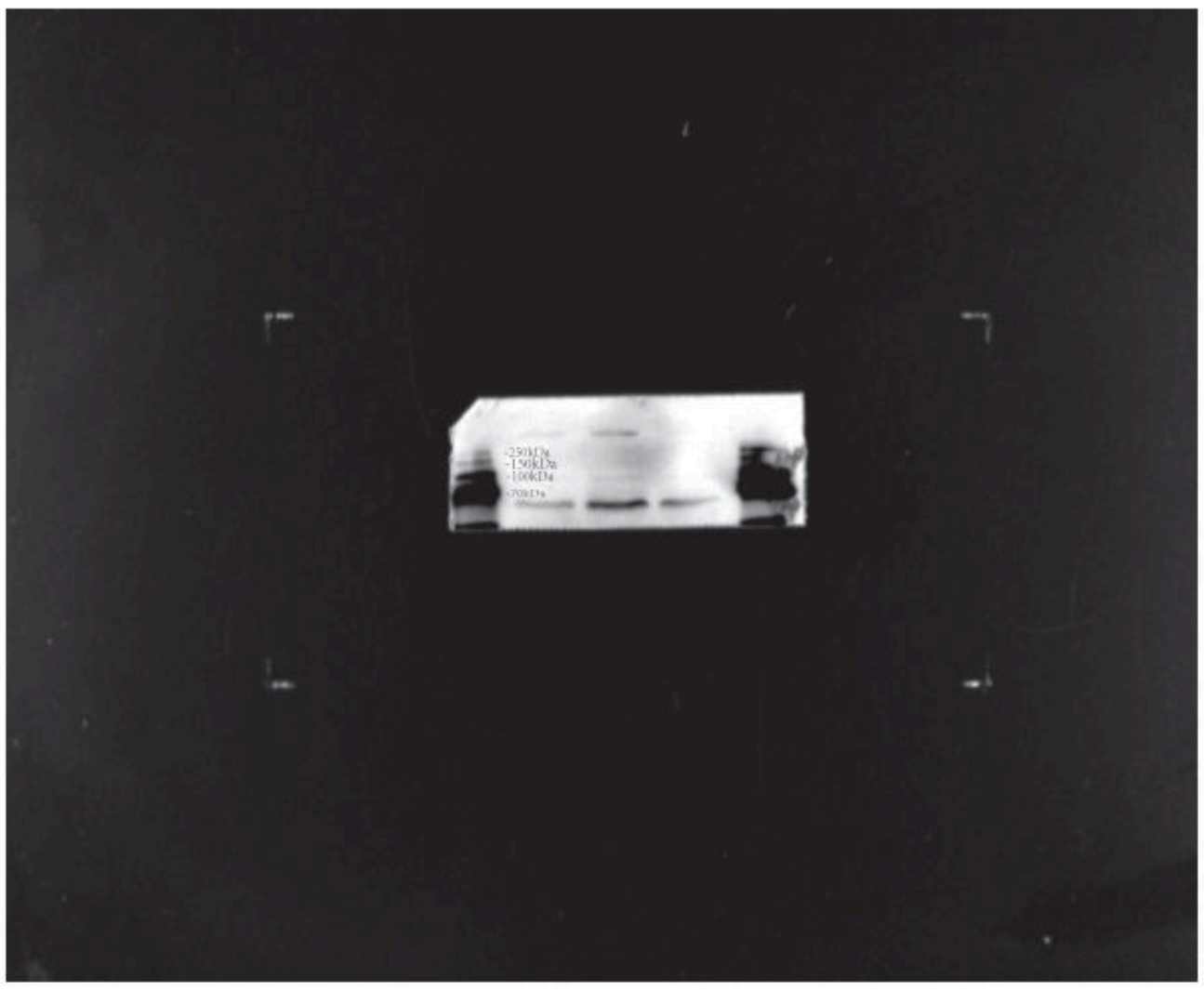

NF-κB 70kDa

P-NF-κB 70kDa

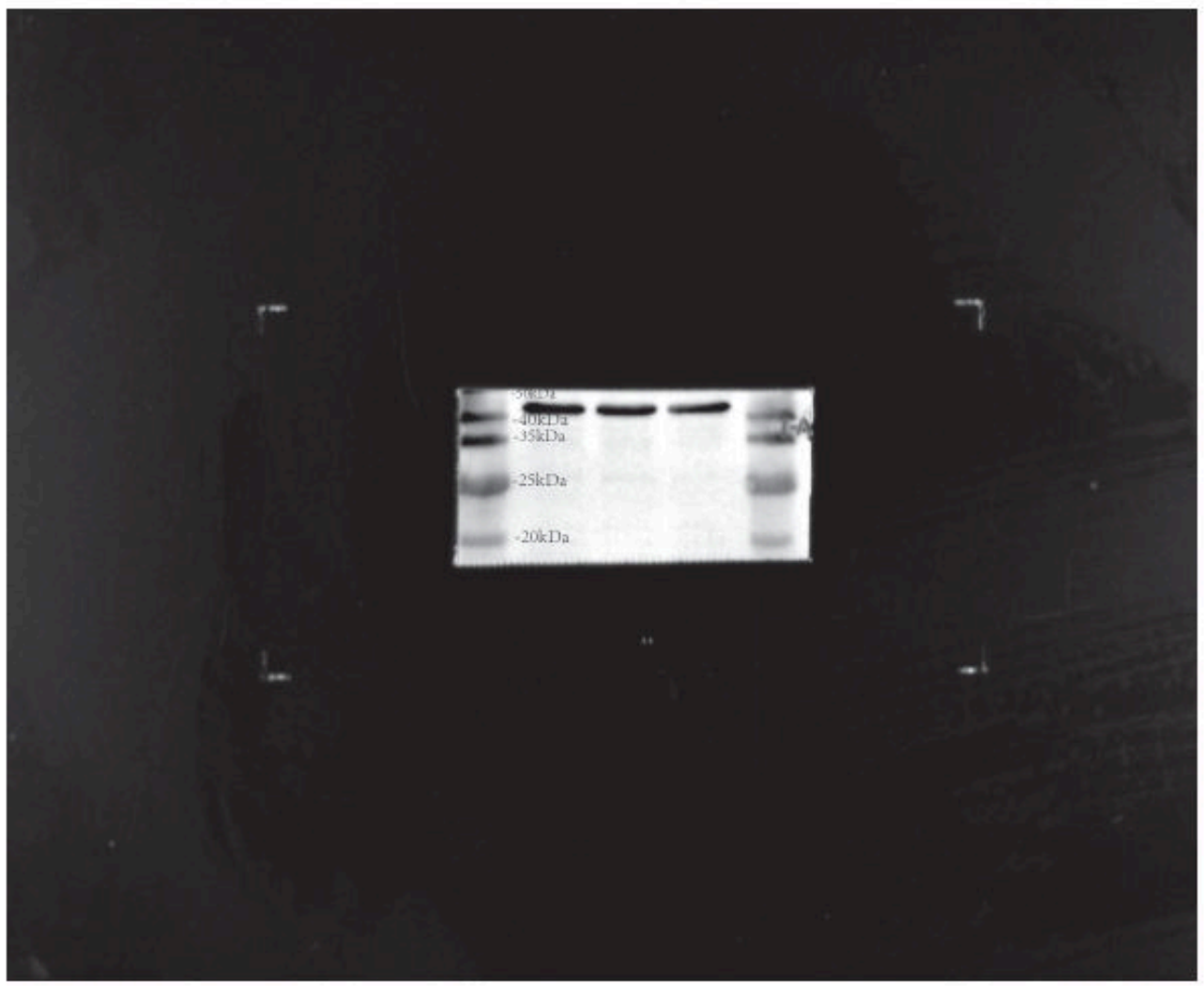
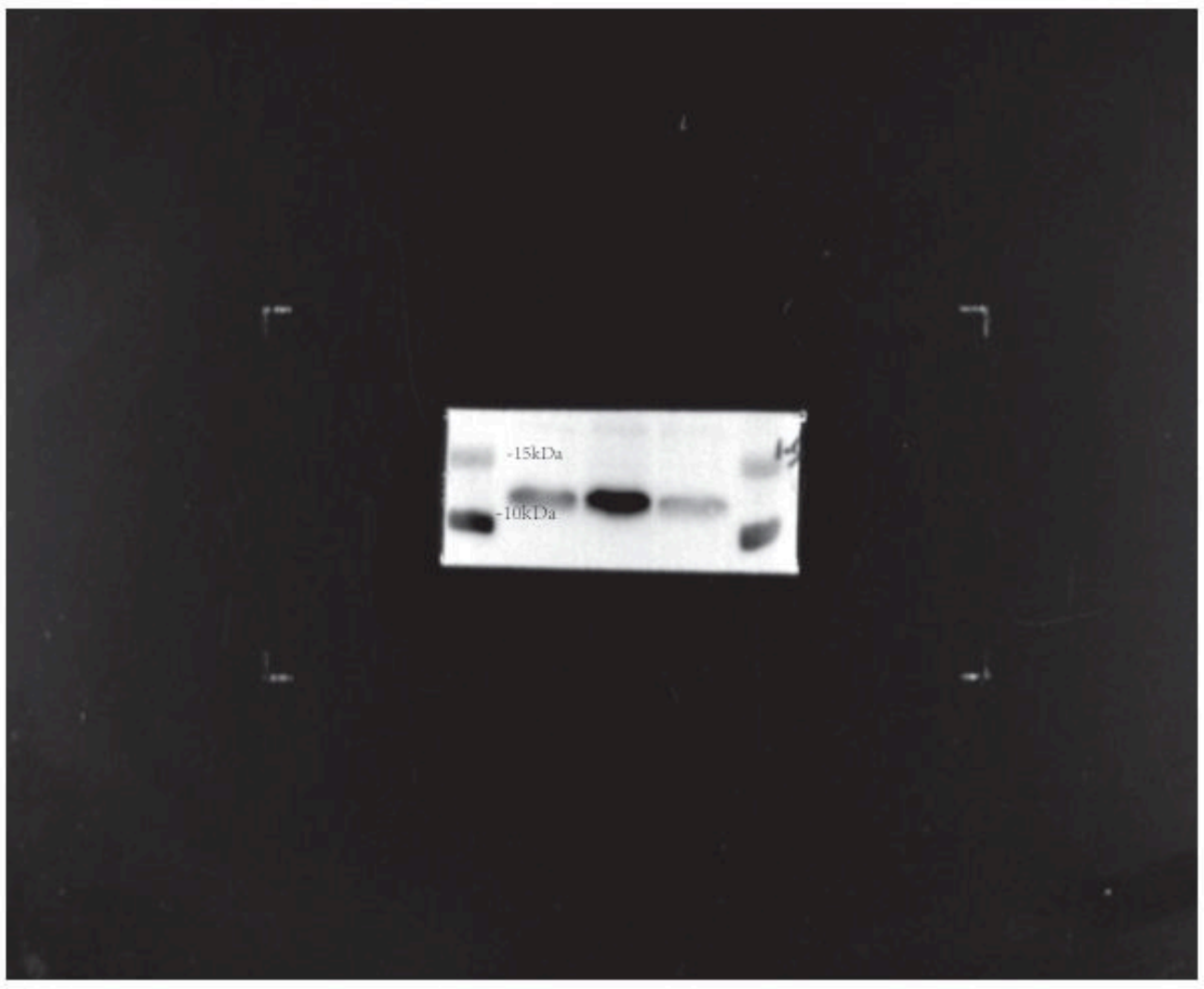

β-actin 42kDa

S100A8 11kDa

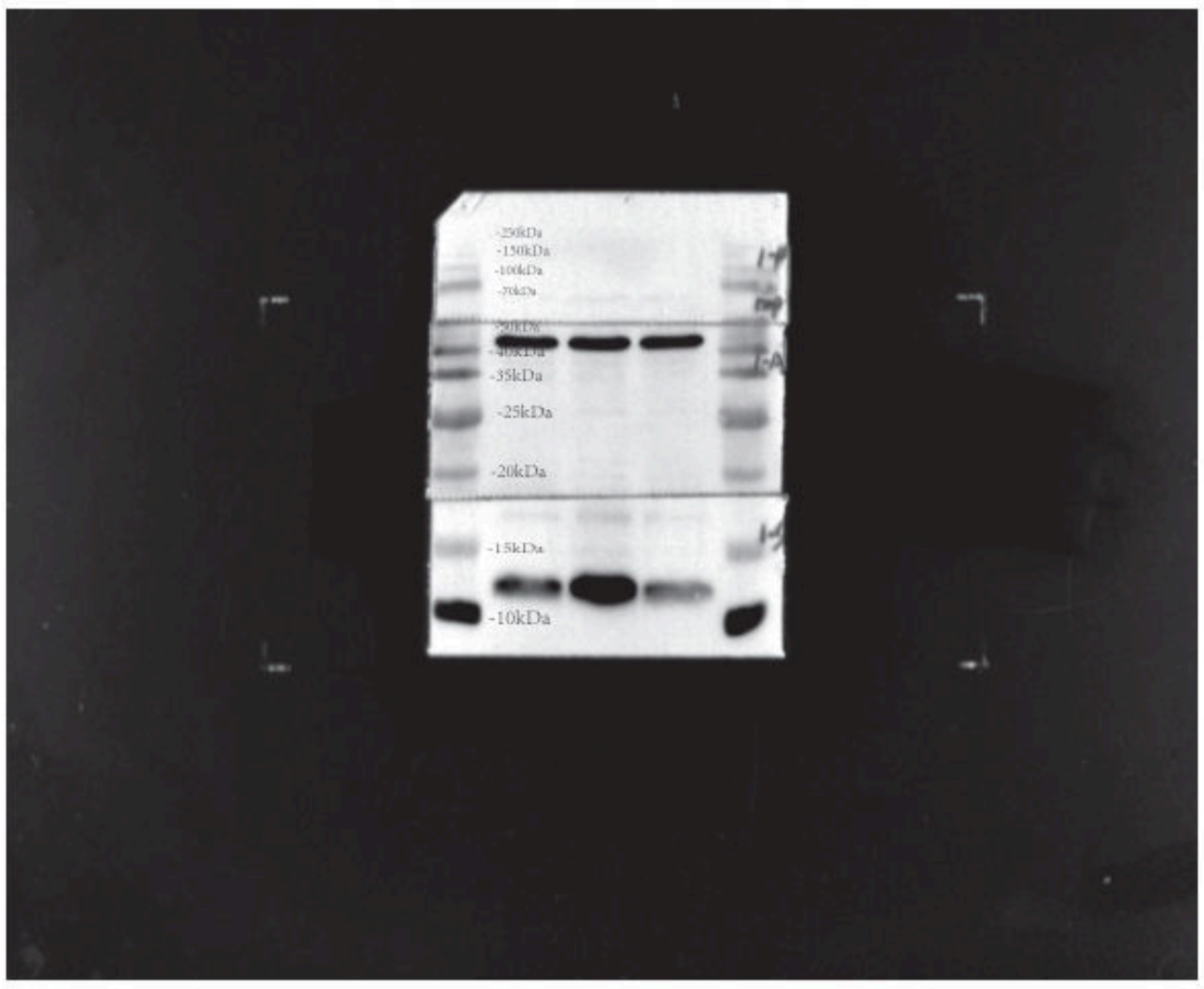
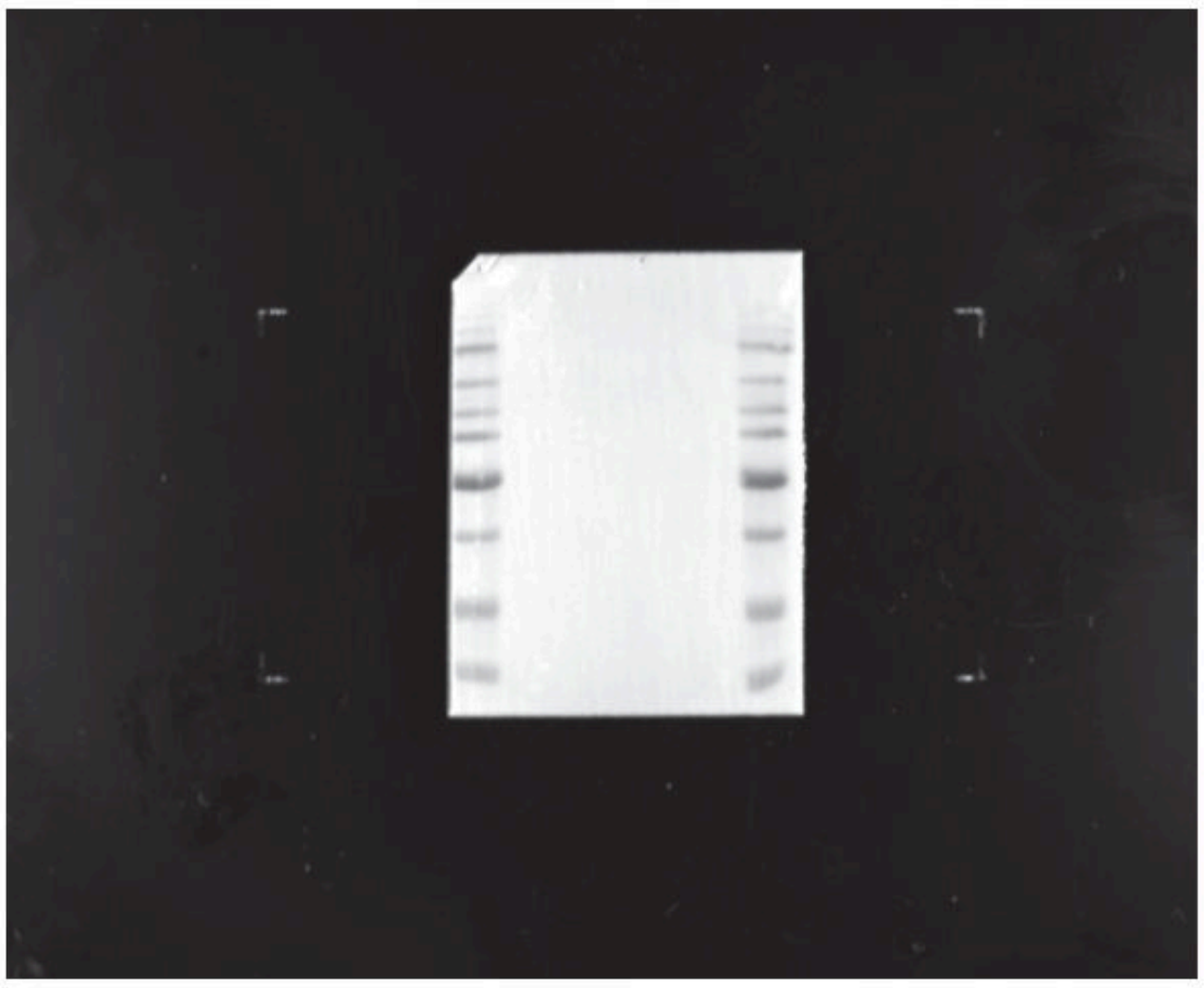

Source: Figure S4.pdf, original page 3. No brightness/contrast/gamma adjustment applied during compilation.

Western blot - crop mapping - repeat 1

Author-supplied crop layout corresponding to the three-condition blots. Final mapping: Fig 7B-C.

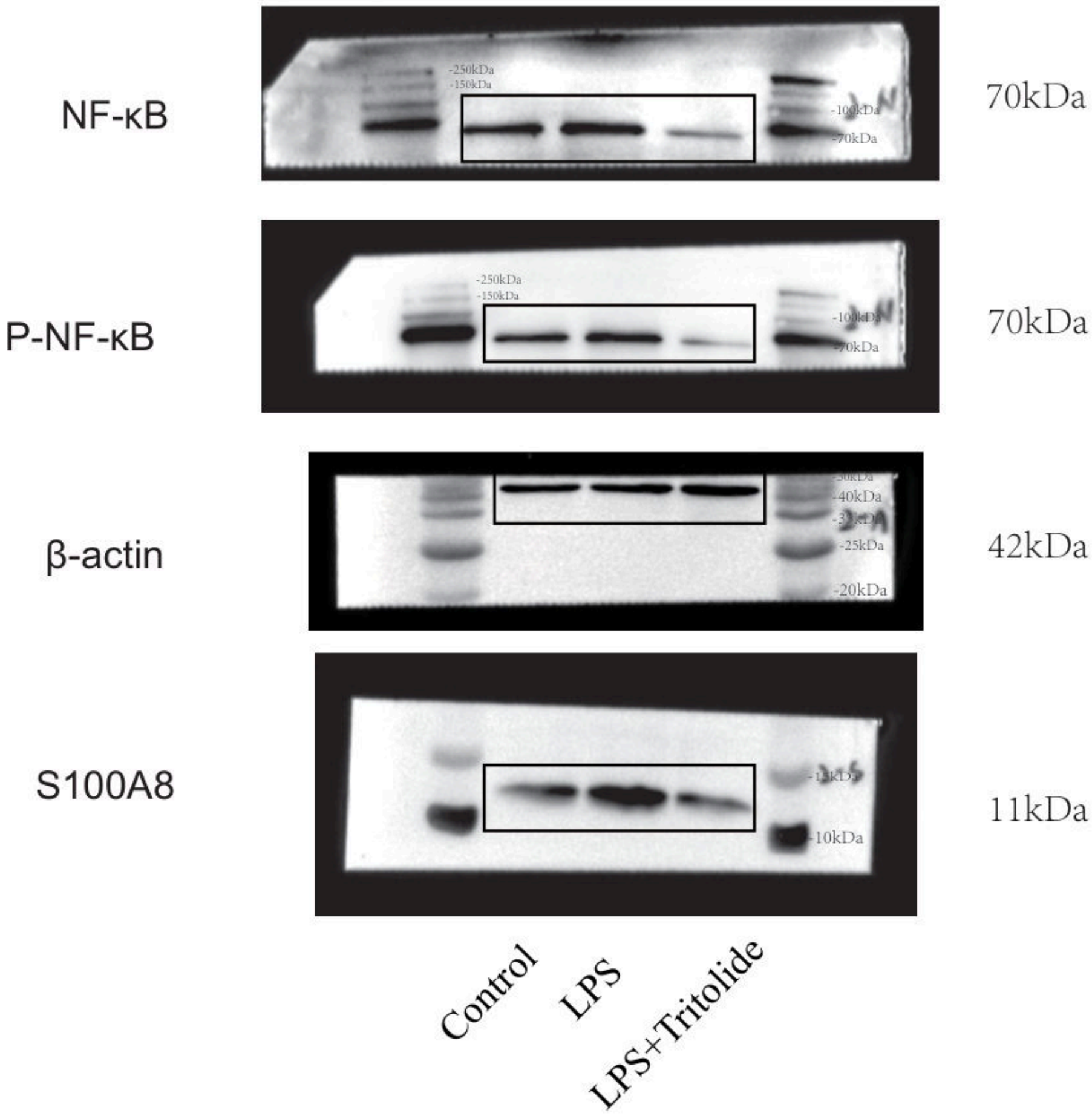

Source: Figure S4.pdf, original page 5. No brightness/contrast/gamma adjustment applied during compilation.

Western blot - crop mapping - repeat 2

Author-supplied crop layout corresponding to the three-condition blots. Final mapping: Fig 7B-C.

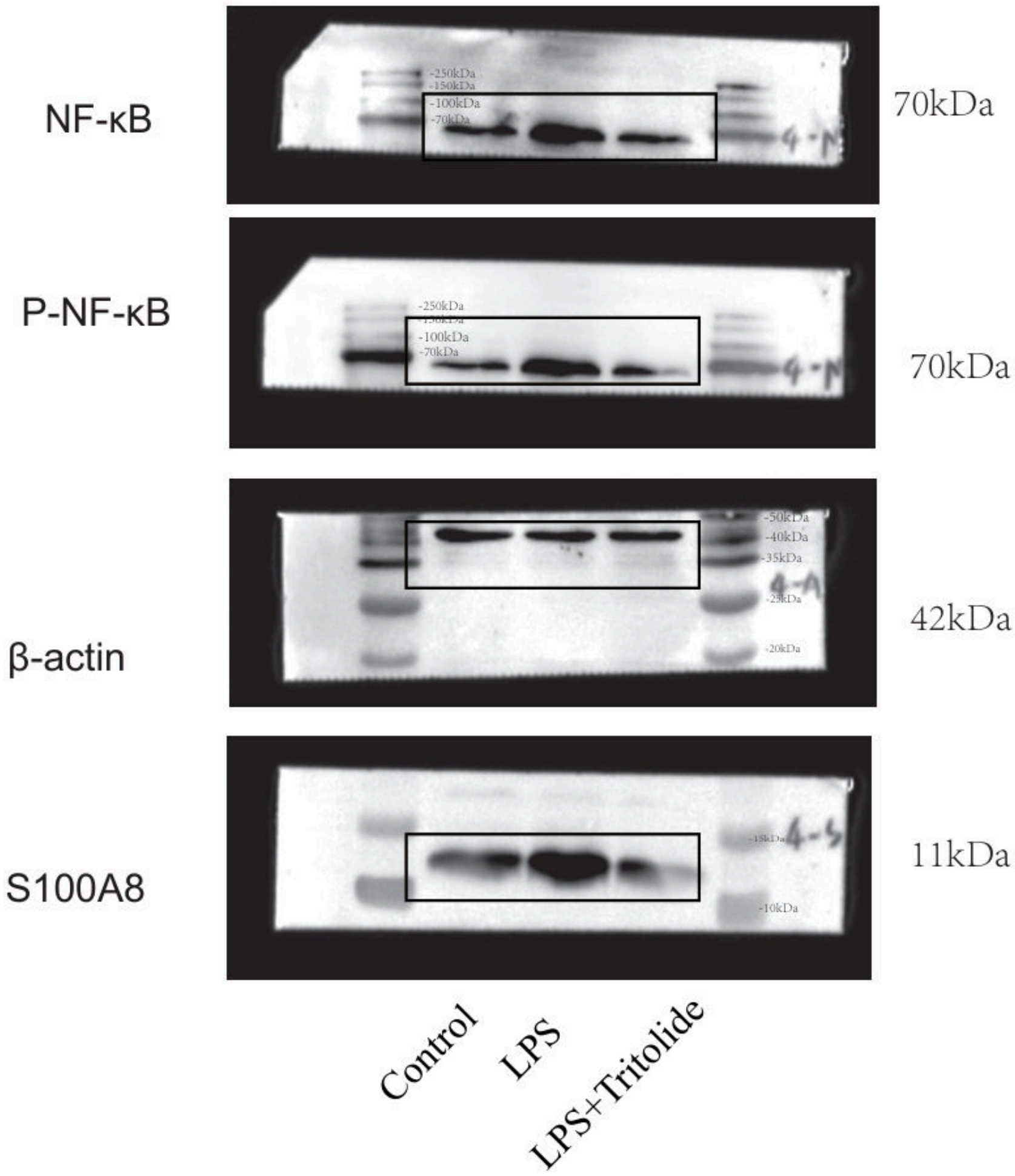

Source: Figure S4.pdf, original page 6. No brightness/contrast/gamma adjustment applied during compilation.

Western blot - crop mapping - repeat 3

Author-supplied crop layout corresponding to the three-condition blots. Final mapping: Fig 7B-C.

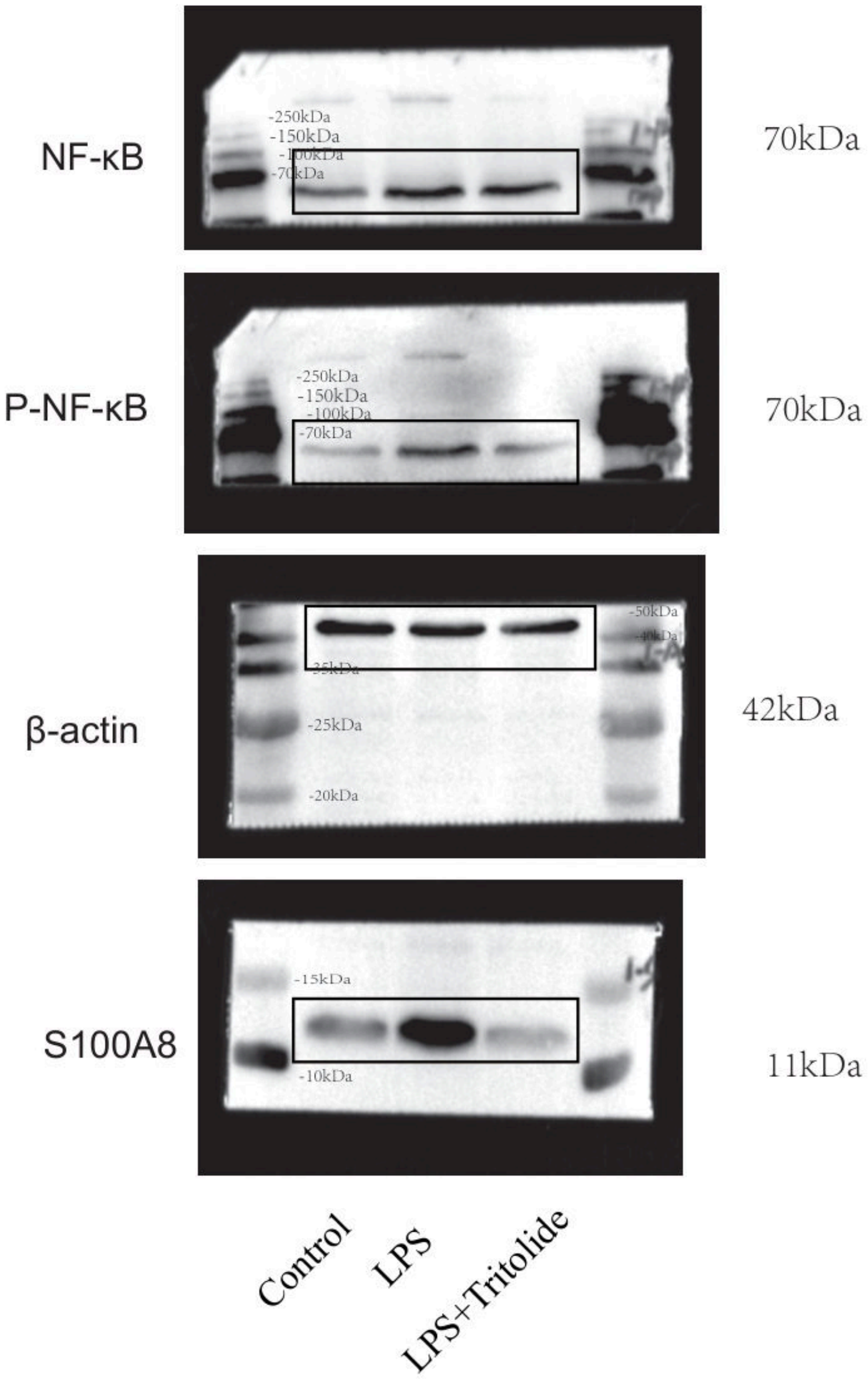

Source: Figure S4.pdf, original page 7. No brightness/contrast/gamma adjustment applied during compilation.

CETSA - full imaging field - repeat 1

Increasing temperature series for DMSO and TPL; target S100A8. Final mapping: Fig 7D-E.

CESTA

repeat 1

DMSO

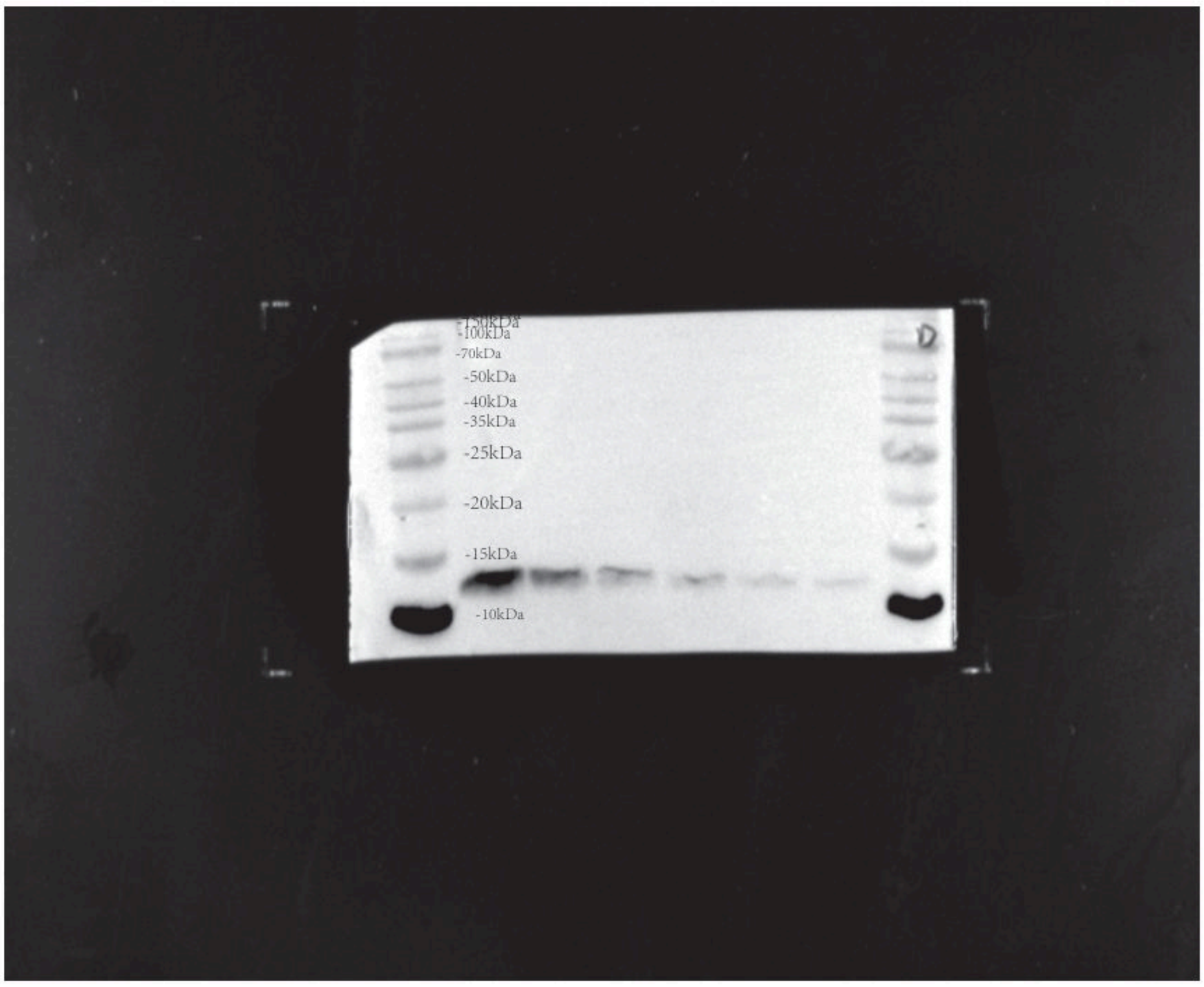

TPL

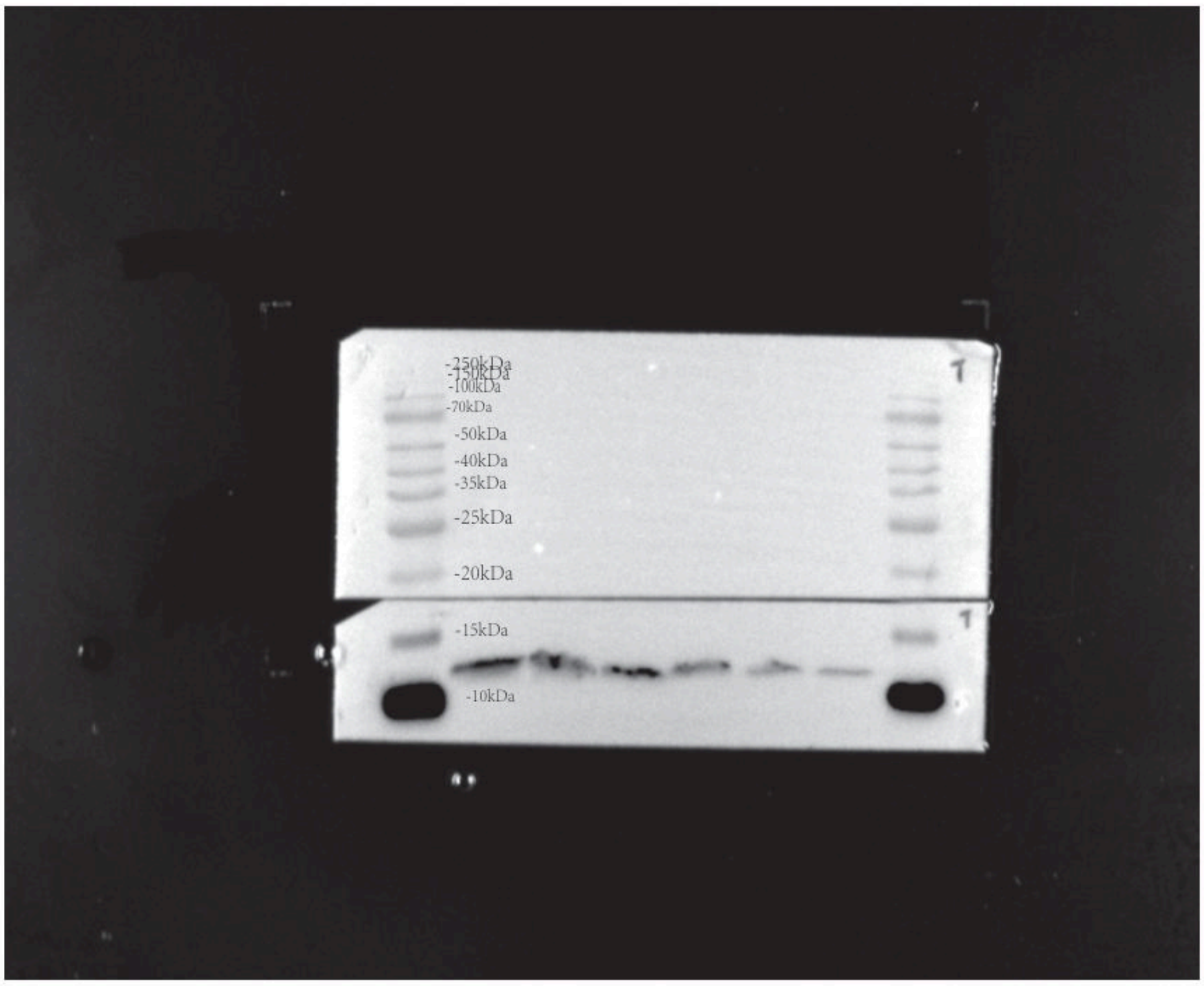

CETSA - full imaging field - repeat 2

Increasing temperature series for DMSO and TPL; target S100A8. Final mapping: Fig 7D-E.

repeat 2

DMSO

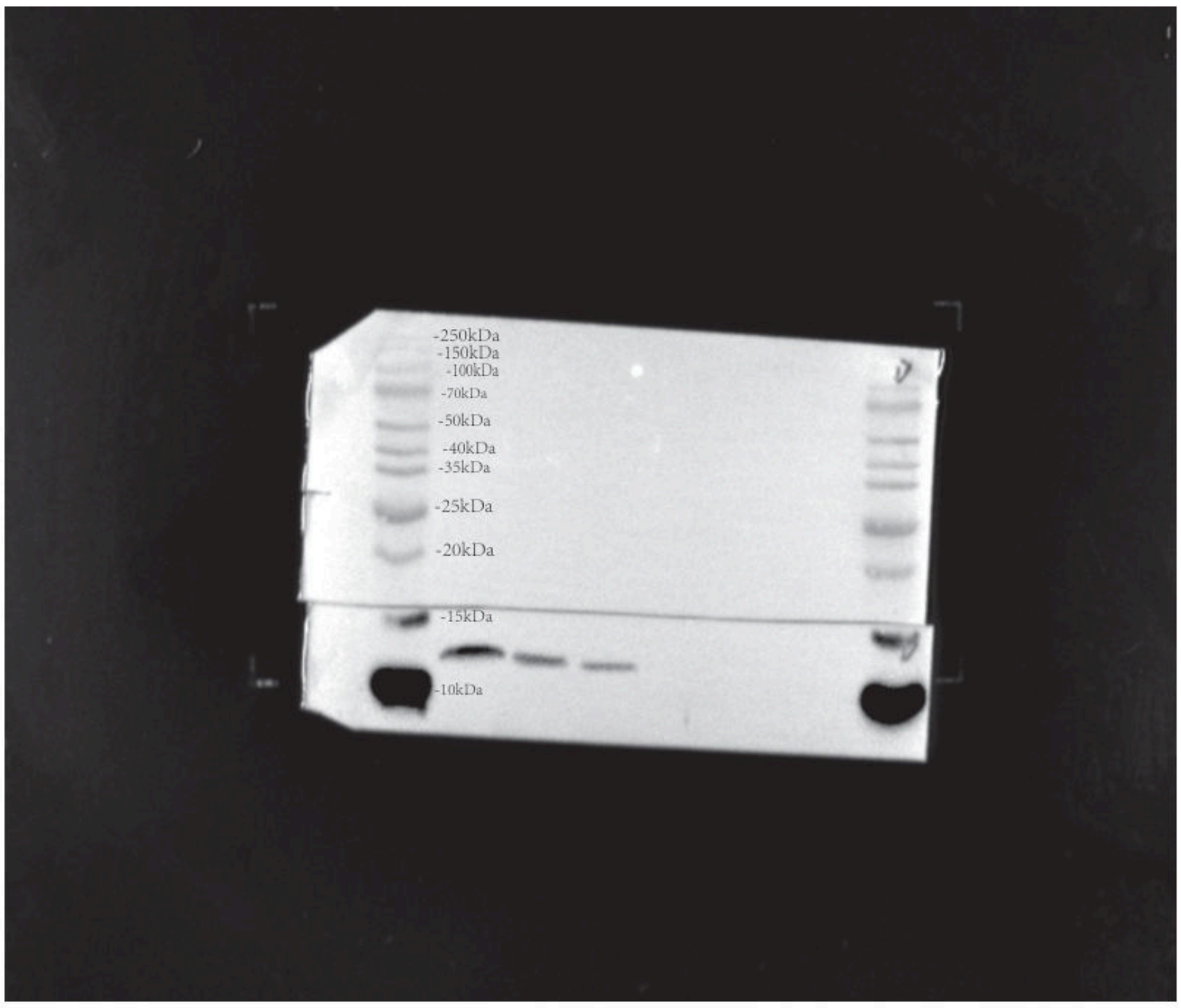

TPL

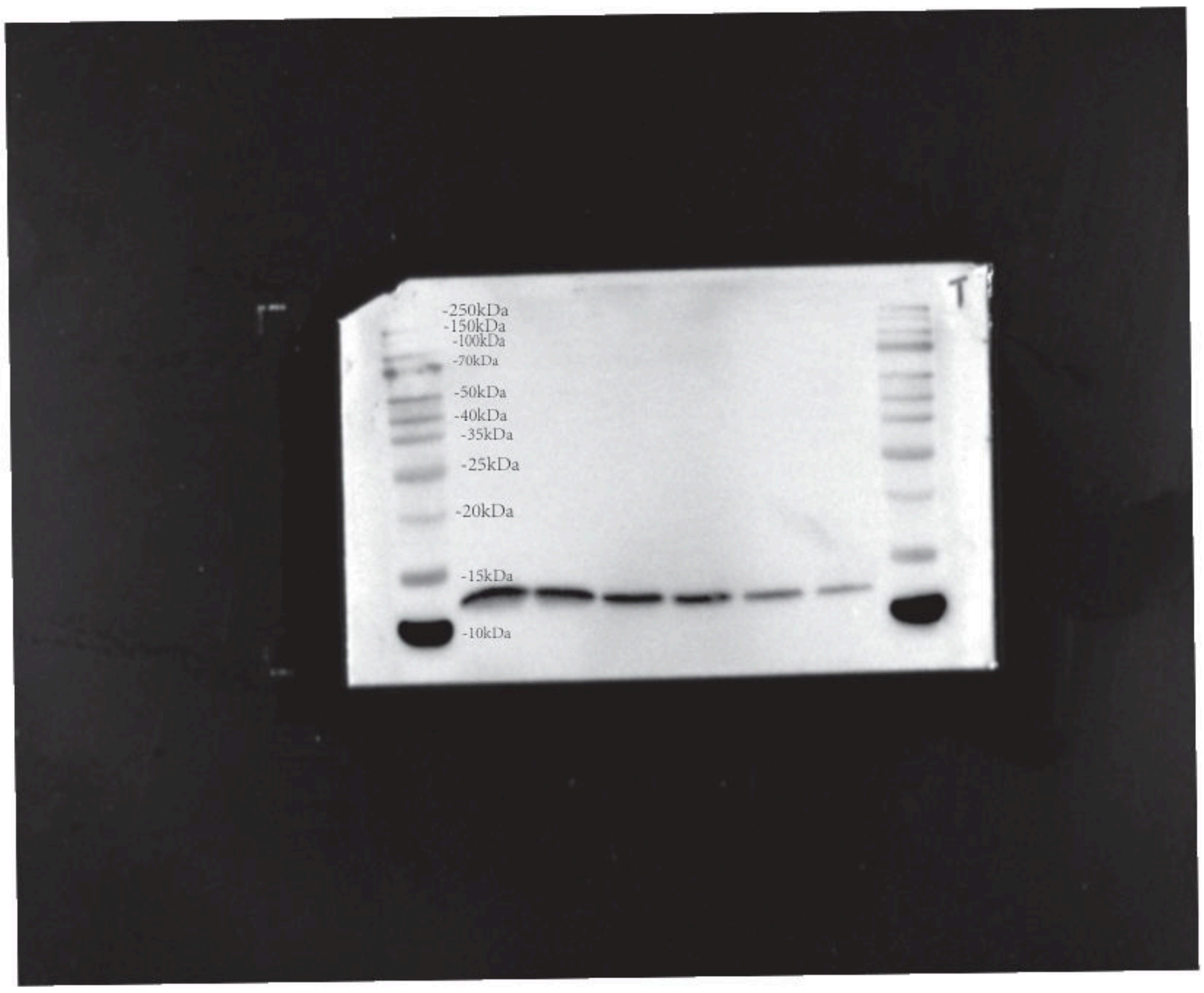

CETSA - full imaging field - repeat 3

Increasing temperature series for DMSO and TPL; target S100A8. Final mapping: Fig 7D-E.

repeat 3

DMSO

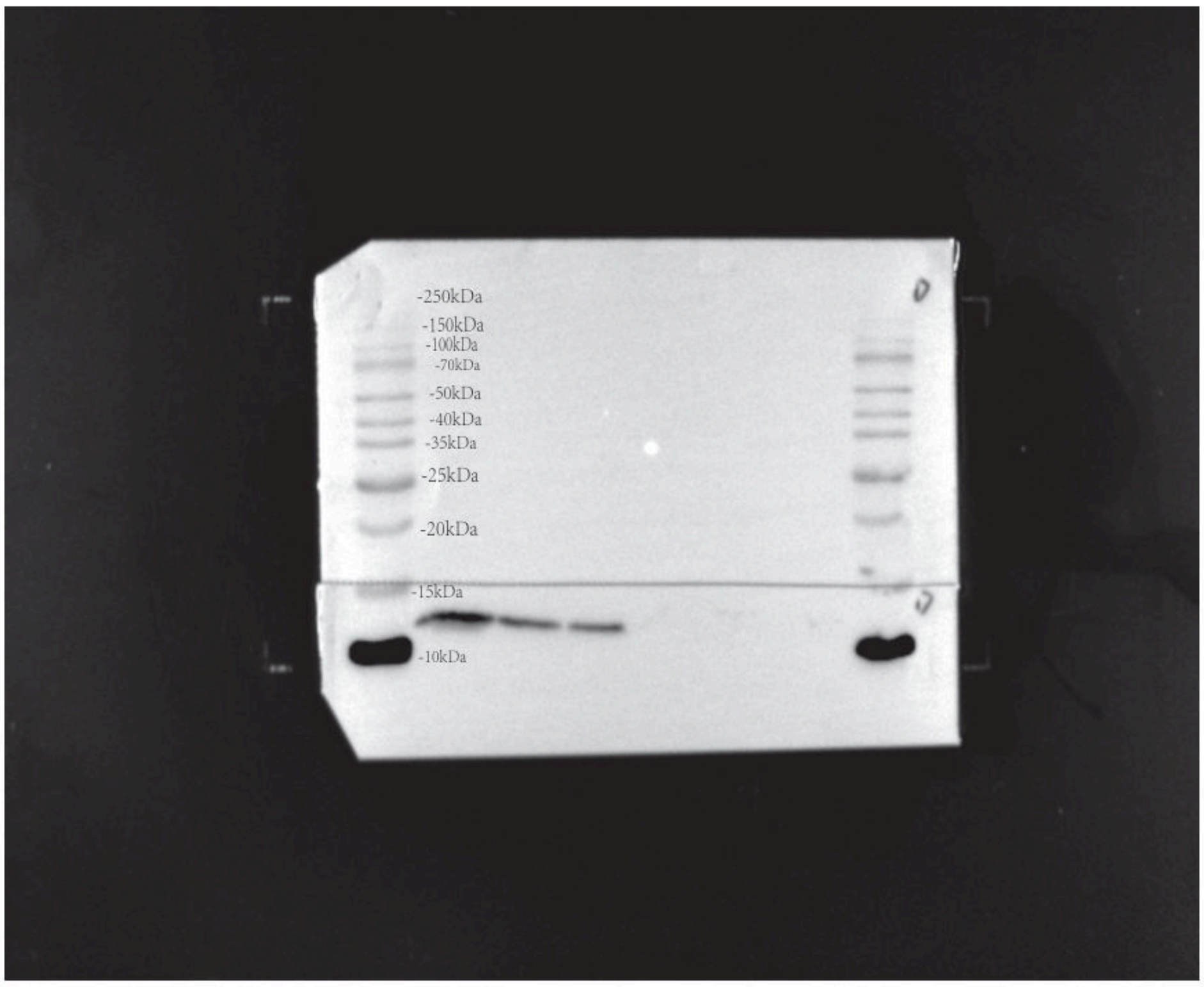

TPL

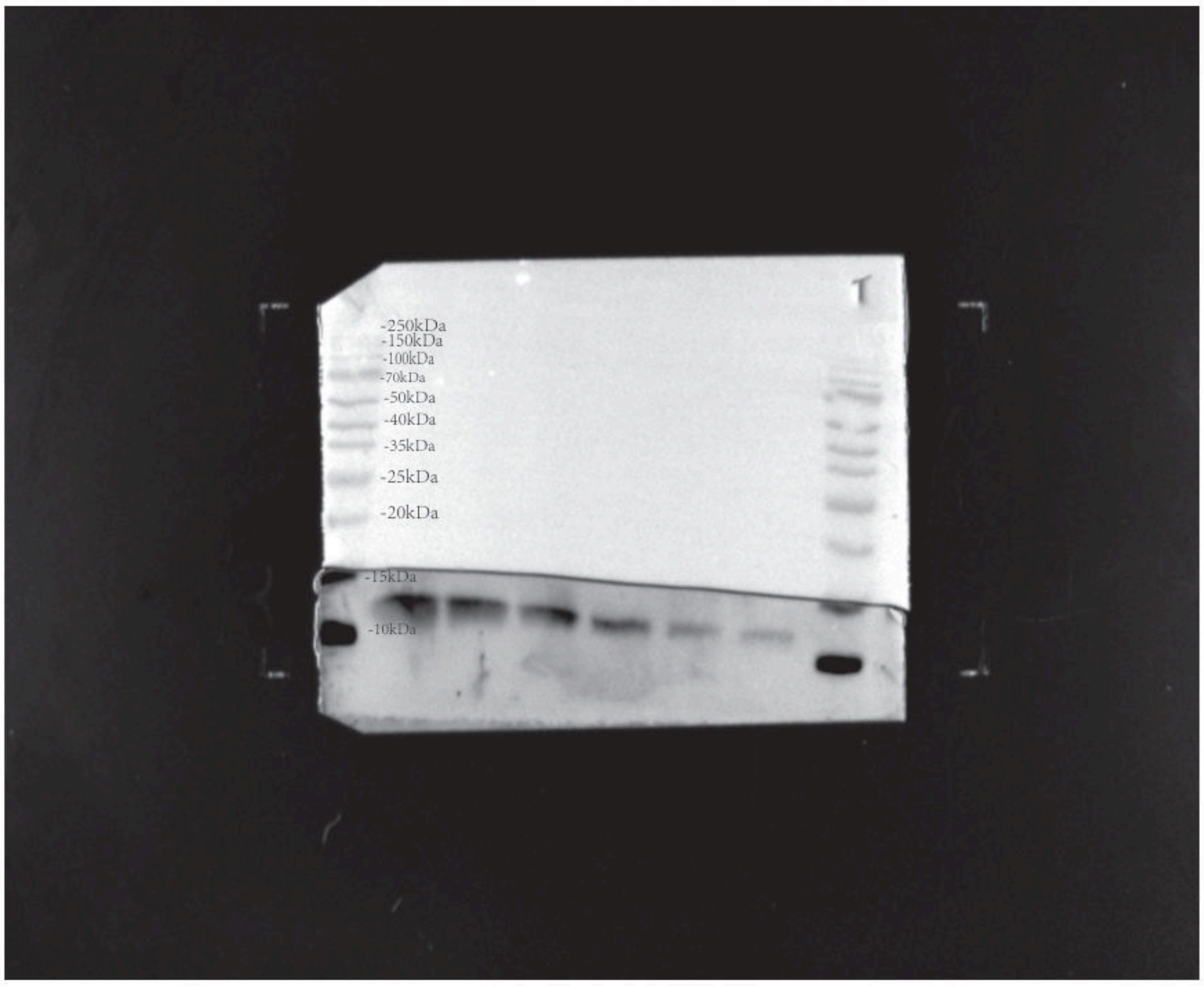

Source: Figure S5.pdf, original page 3. No brightness/contrast/gamma adjustment applied during compilation.

CETSA - crop mapping

Author-supplied S100A8 temperature-series crop layout. Final mapping: Fig 7D-E.

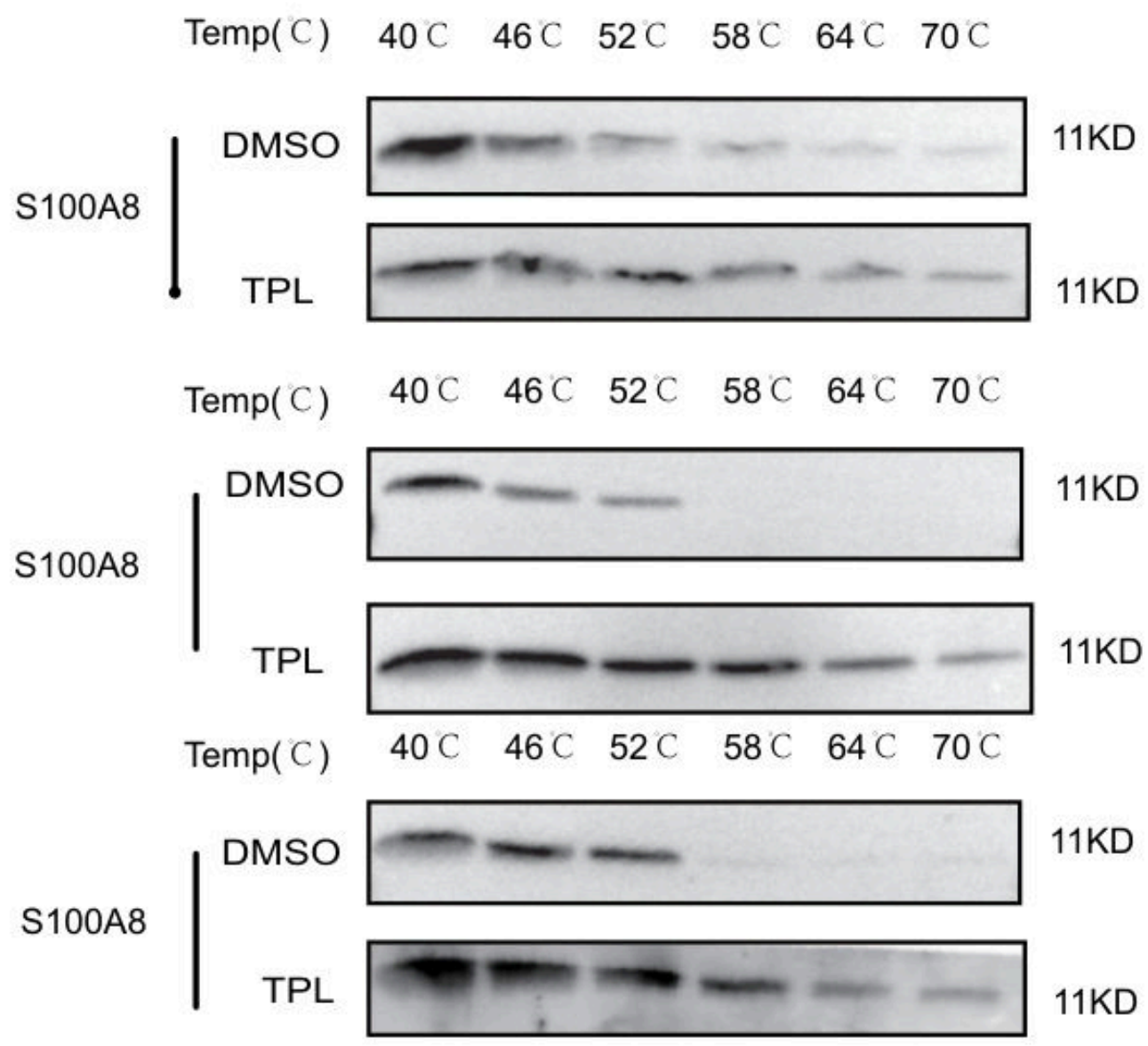

source data for CESTA

Target	Sample	Temperature(℃)	Relative Grey Density 1	Relative Grey Density 2	Relative Grey Density 3	Relative Expression 1	Relative Expression 2	Relative Expression 3
S100A8	DMSO	40℃	27113.25	26441.2	29858.44	1	1	1
		46℃	18126.1	18644.64	16434.35	0.66853316	0.70513603	0.550408781
		52℃	11960.59	17359.08	14480.59	0.44113453	0.65651626	0.48497475
		58℃	9257.711	12887.22	4804.104	0.34144606	0.48739161	0.16089602
		64℃	5670.761	5961.64	3935.64	0.20915094	0.22546786	0.131809972
		70℃	2199.933	2587.104	1817.296	0.08113868	0.09784368	0.060863731
	Tritolide	40℃	24366.903	30353.489	26750.418	1	1	1
		46℃	22566.761	27950.761	20367.711	0.926123	0.920842	0.761398
		52℃	21907.61	28340.811	18891.539	0.899072	0.933692	0.706215
		58℃	15821.075	27101.811	14609.518	0.649285	0.892873	0.546142
		64℃	9338.296	16394.418	7571.104	0.383237	0.540116	0.283028
		70℃	3897.225	7064.468	3274.548	0.159939	0.23274	0.122411

Eight-condition NF-kB/p-NF-kB/beta-actin blot - repeat 1

siNC and siS100A8 x Control, TPL, LPS, and LPS+TPL. Final mapping: Fig 8A-B.

WB

repeat 1

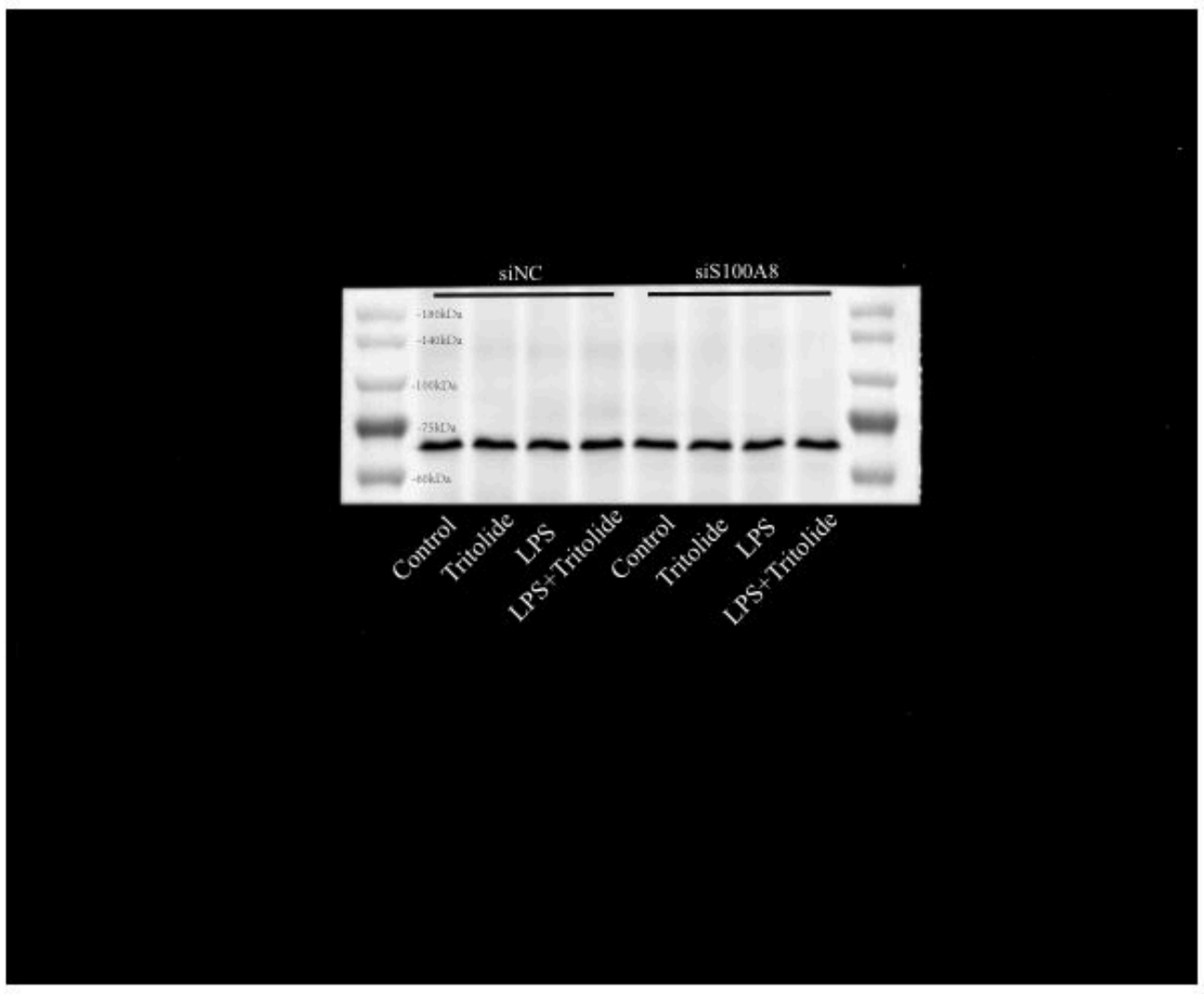

NF-κB 70kDa

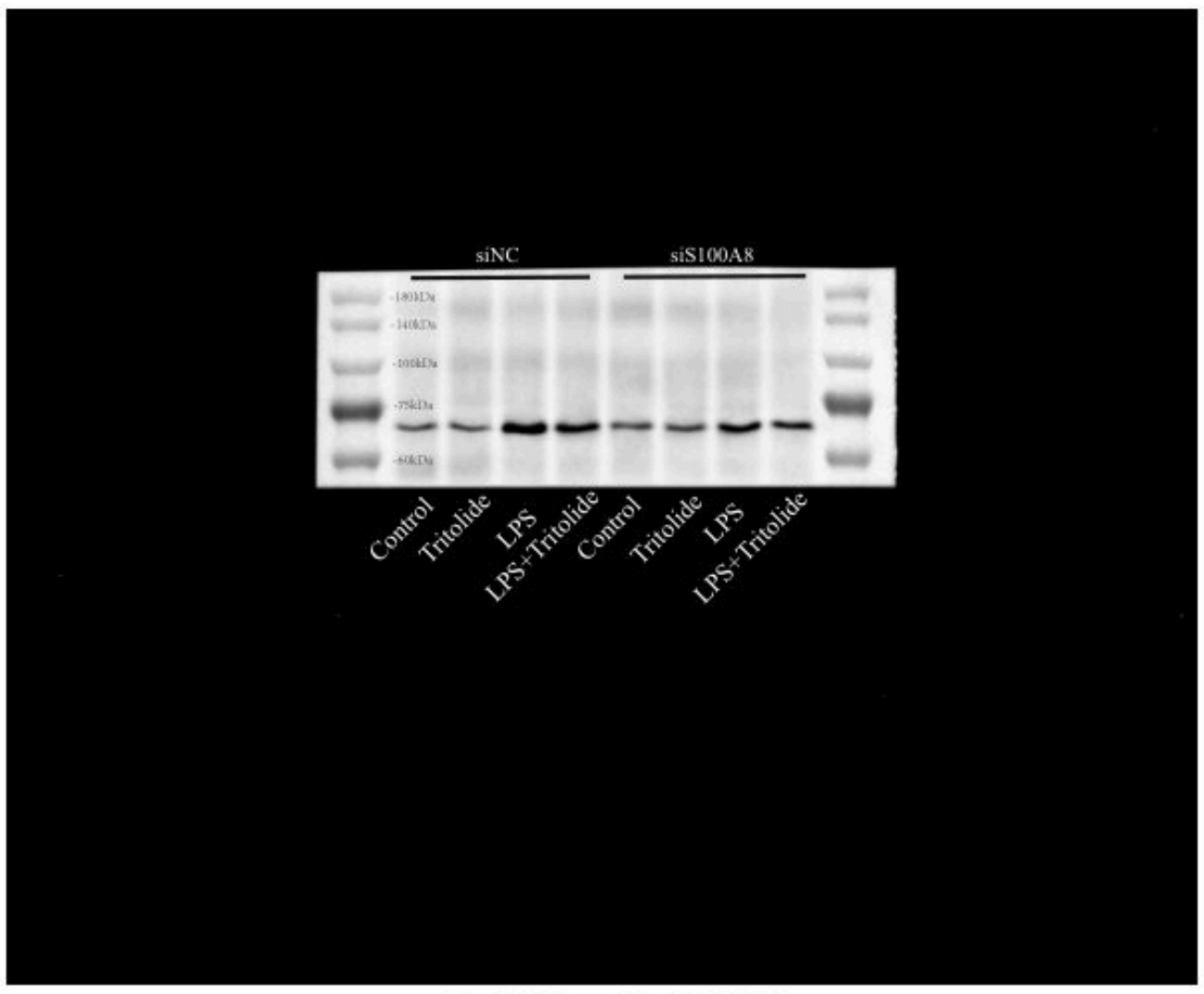

P-NF-κB 70kDa

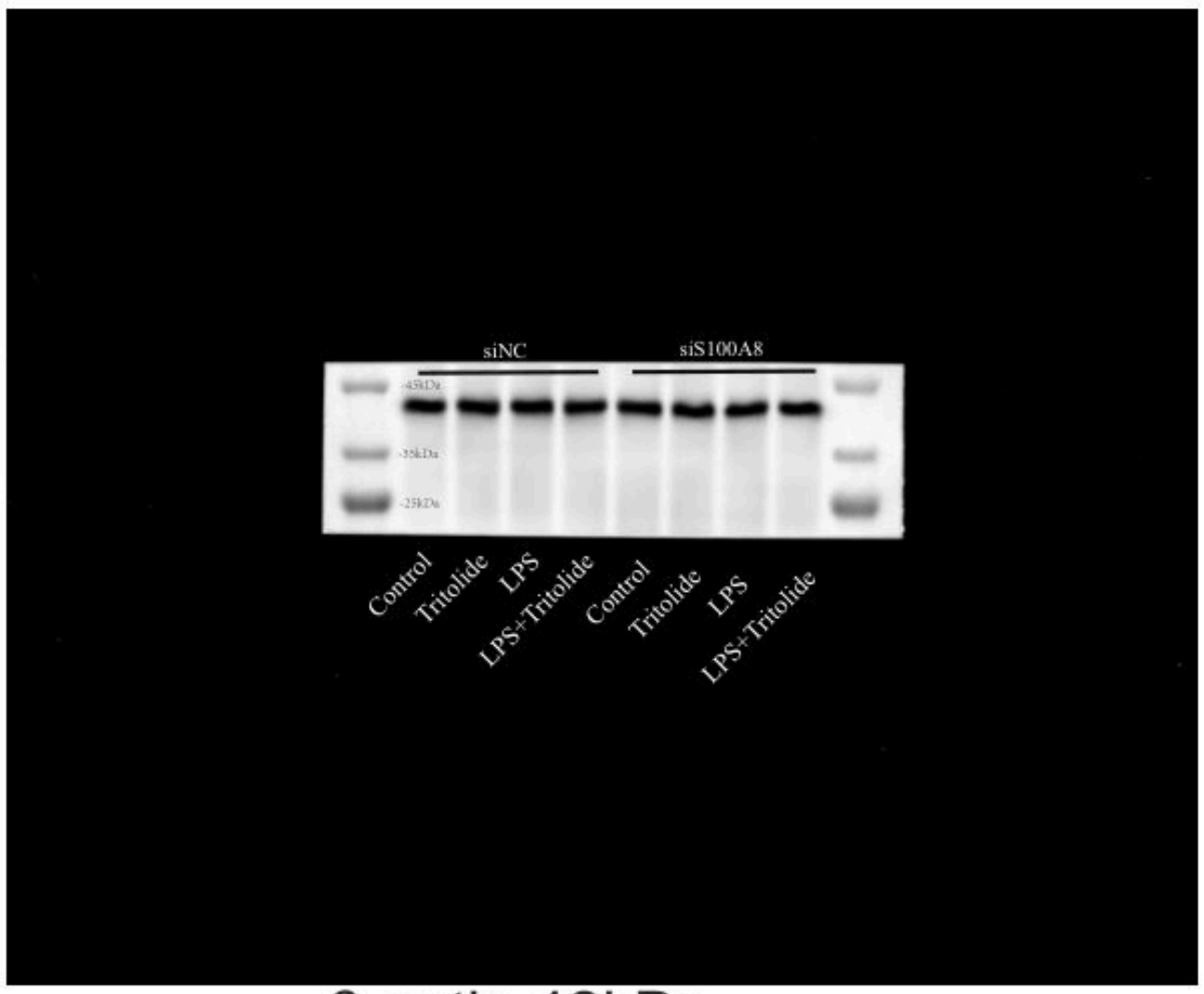

β-actin 42kDa

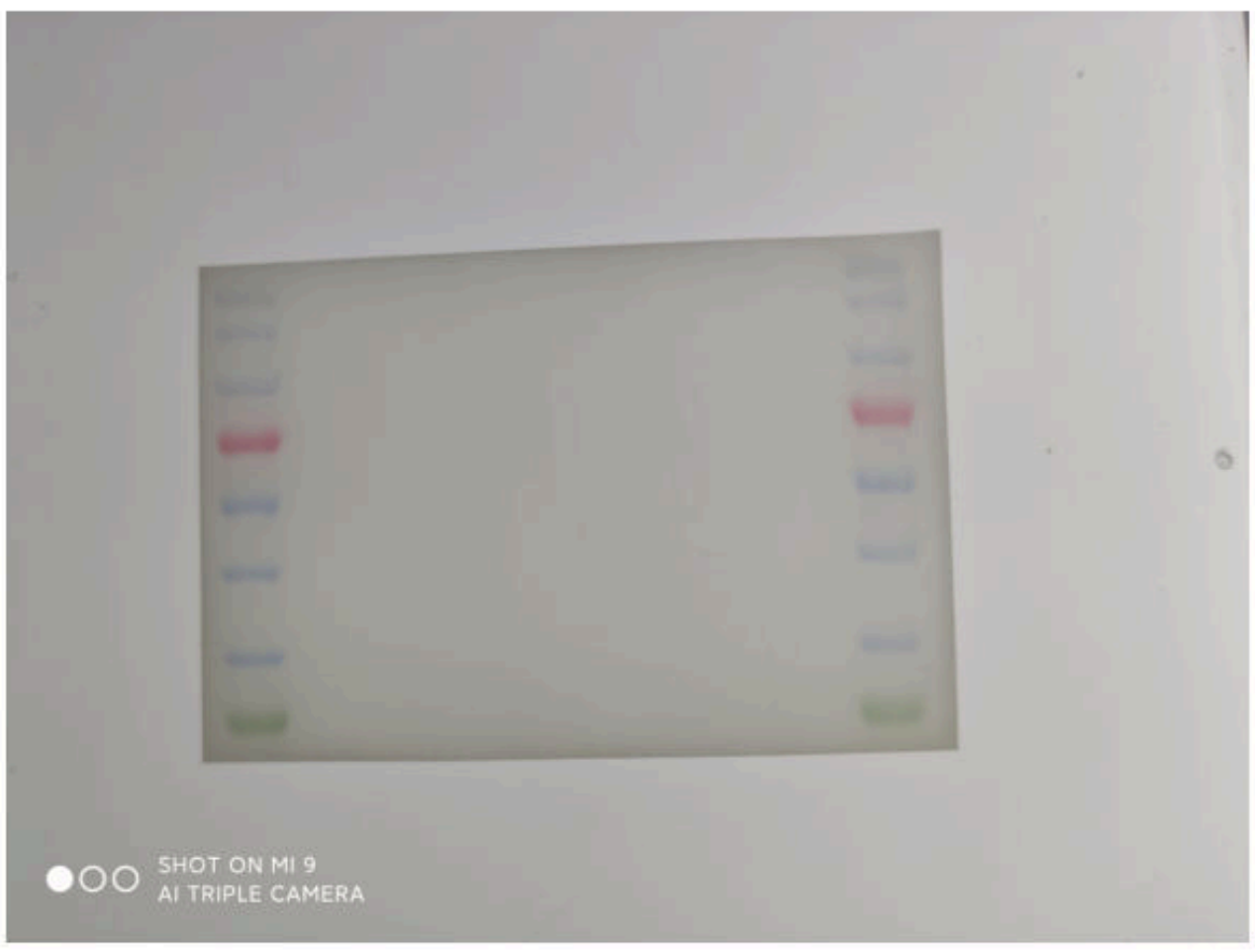

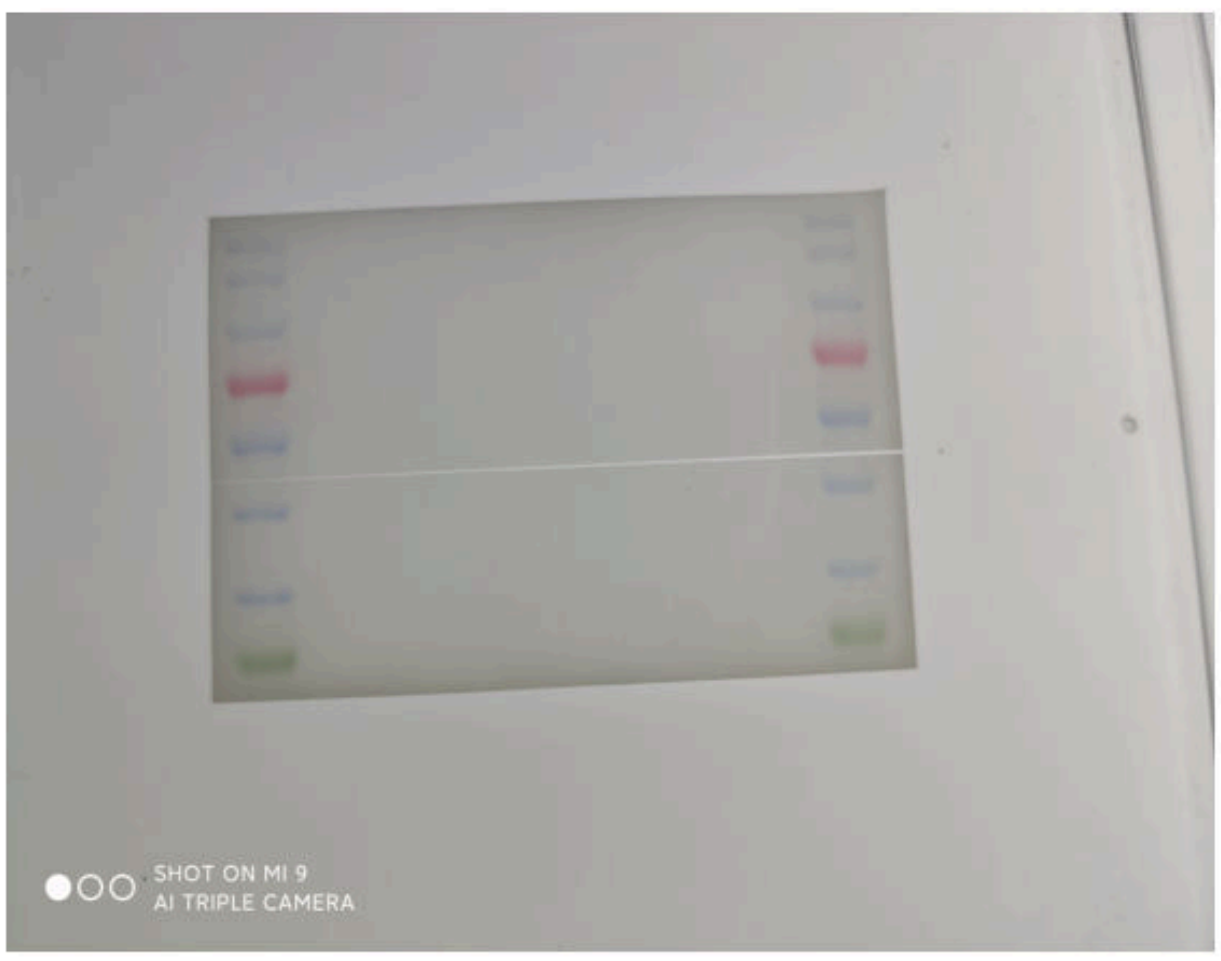

Source: New_validation_WB_source.pdf, original page 1. No brightness/contrast/gamma adjustment applied during compilation.

Eight-condition NF-kB/p-NF-kB/beta-actin blot - repeat 2

siNC and siS100A8 x Control, TPL, LPS, and LPS+TPL. Final mapping: Fig 8A-B.

WB repeat 2

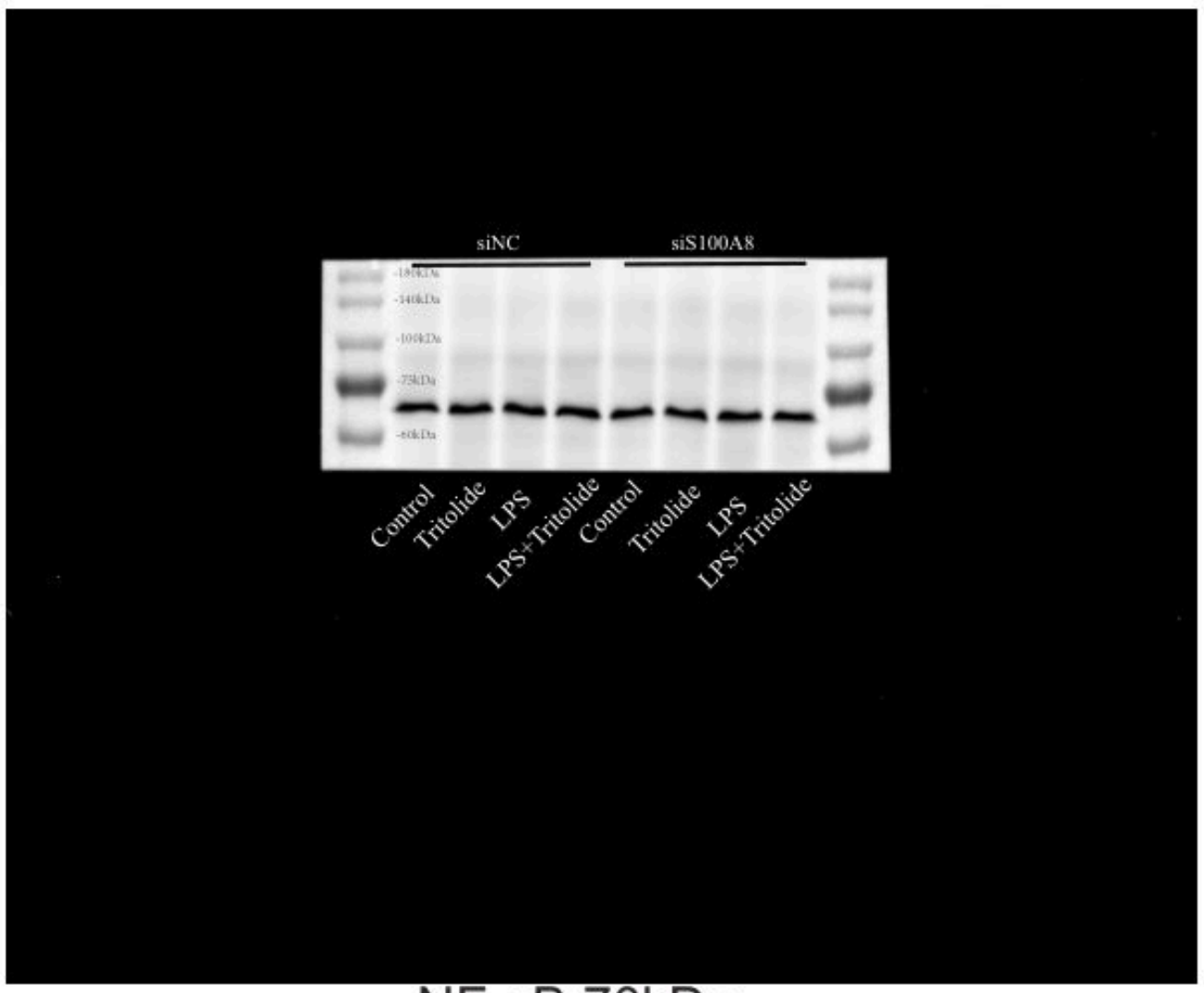

NF-κB 70kDa

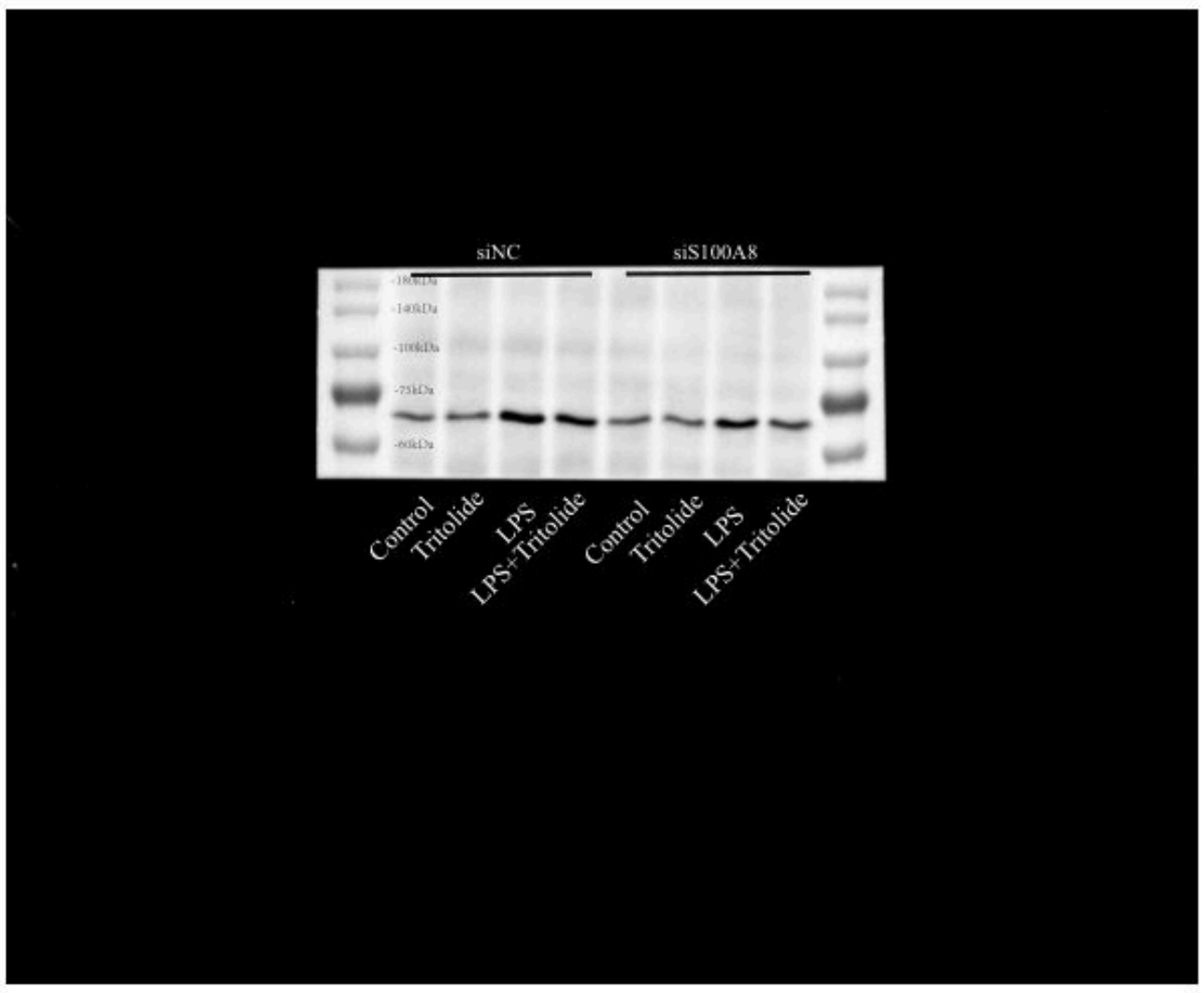

P-NF-κB 70kDa

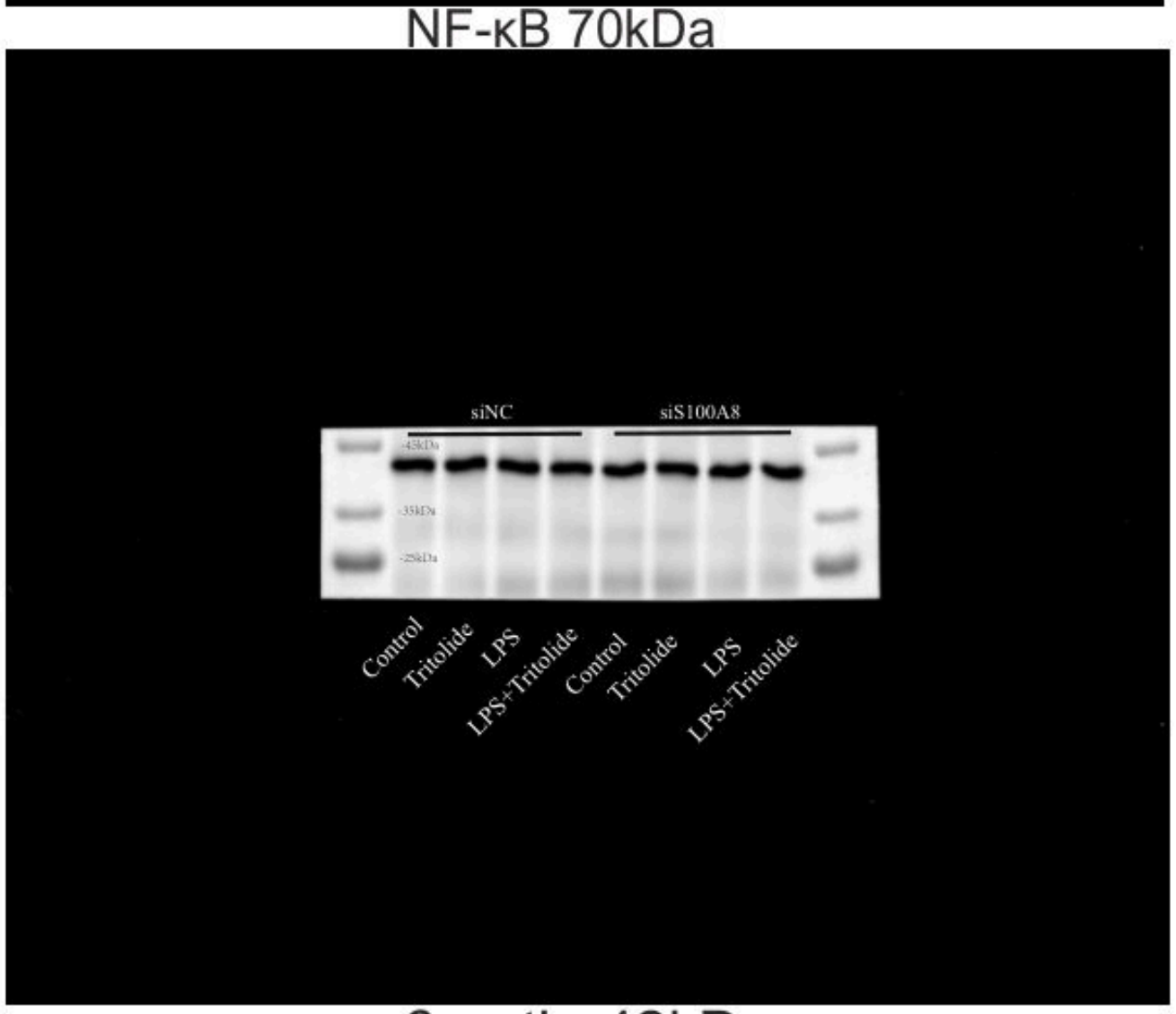

β-actin 42kDa

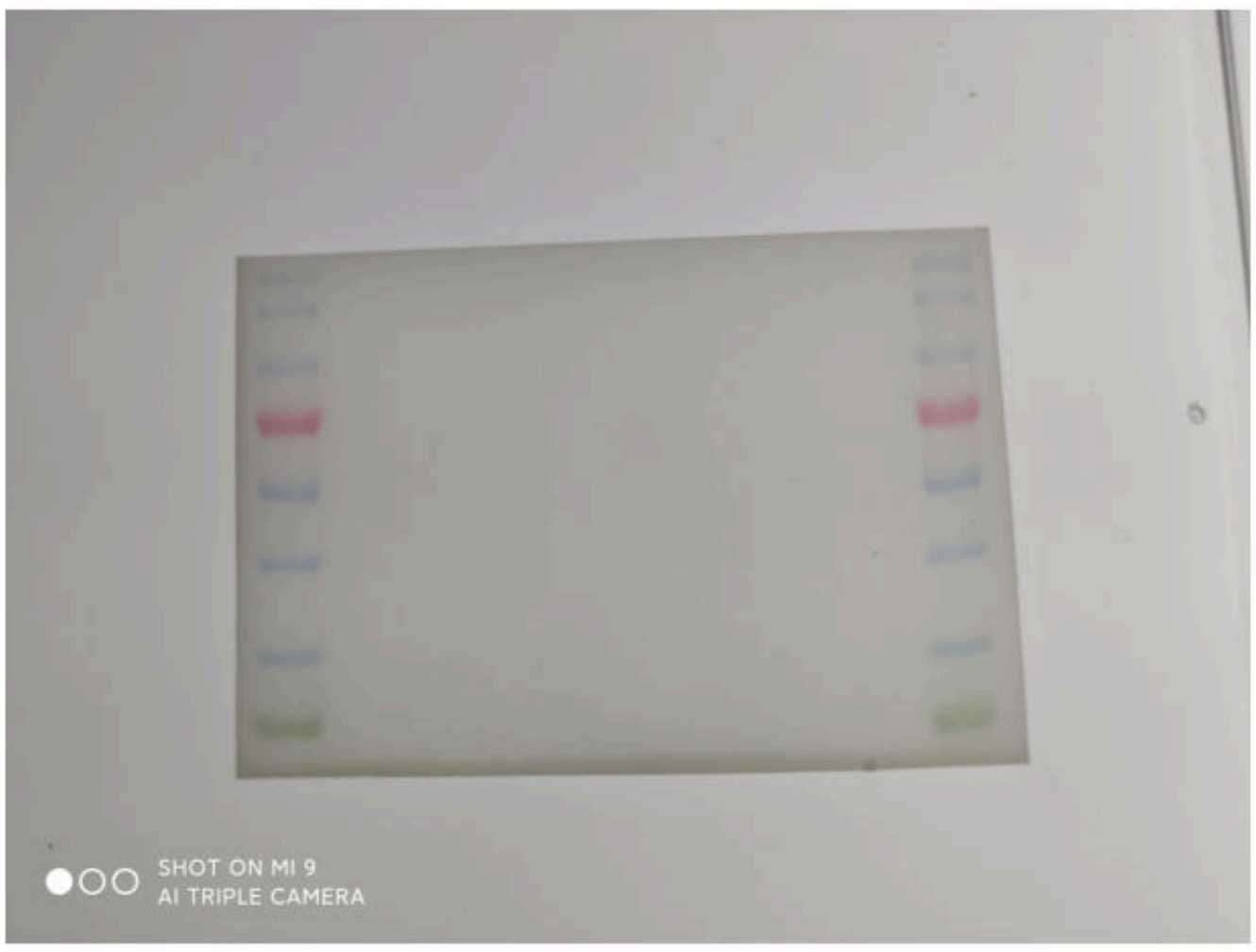

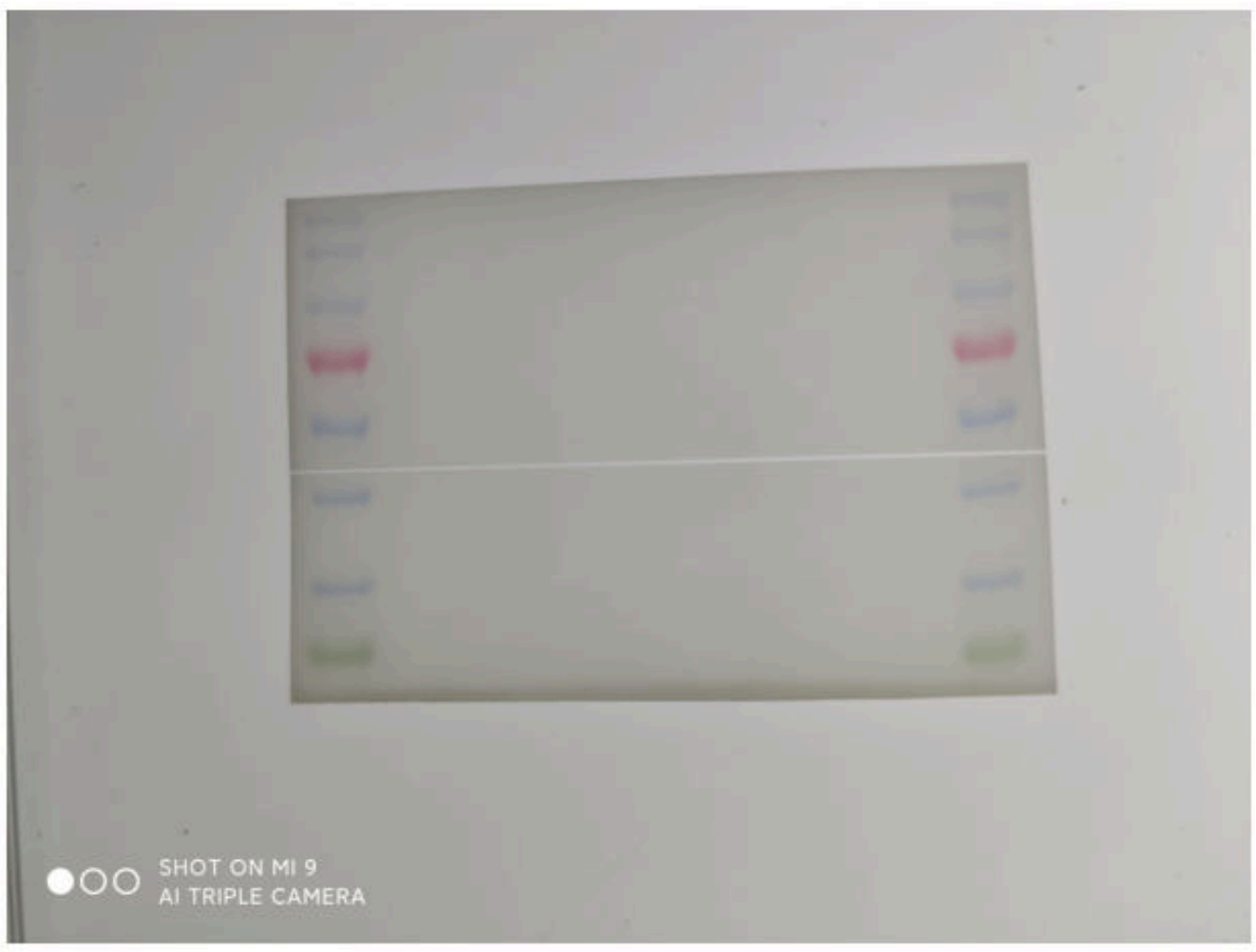

Source: New_validation_WB_source.pdf, original page 2. No brightness/contrast/gamma adjustment applied during compilation.

Eight-condition NF-kB/p-NF-kB/beta-actin blot - repeat 3

siNC and siS100A8 x Control, TPL, LPS, and LPS+TPL. Final mapping: Fig 8A-B.

WB repeat 3

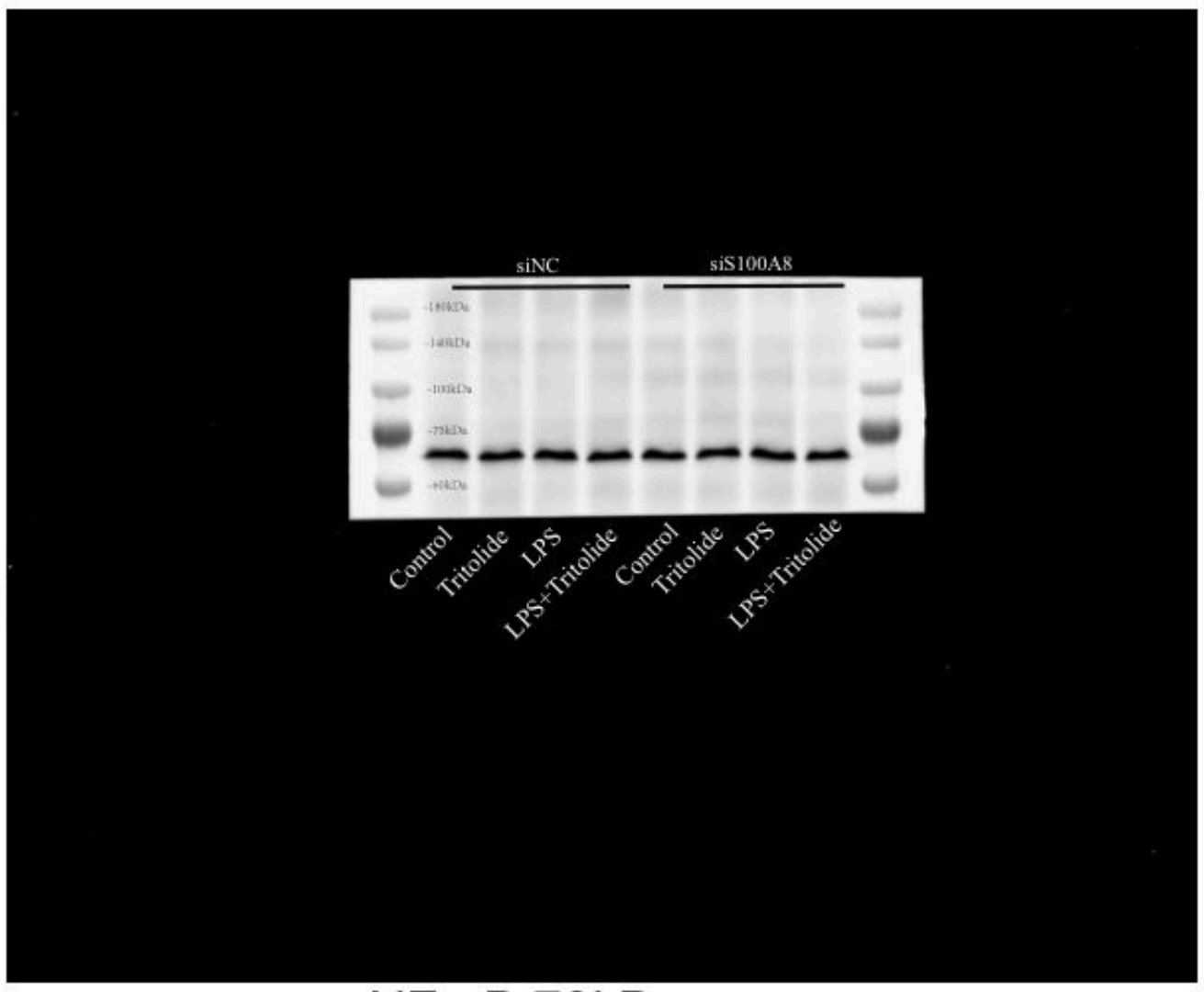

NF-κB 70kDa

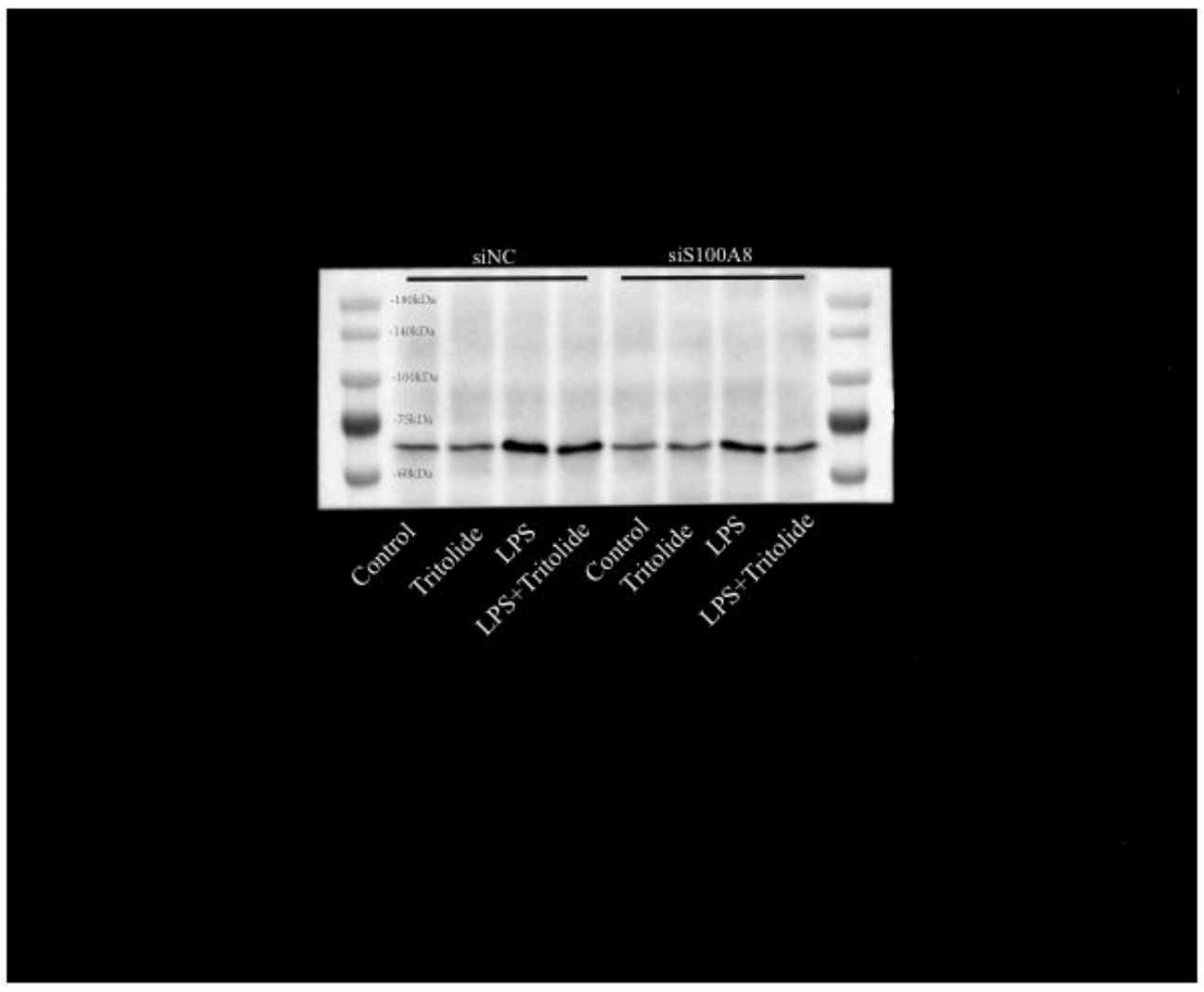

P-NF-κB 70kDa

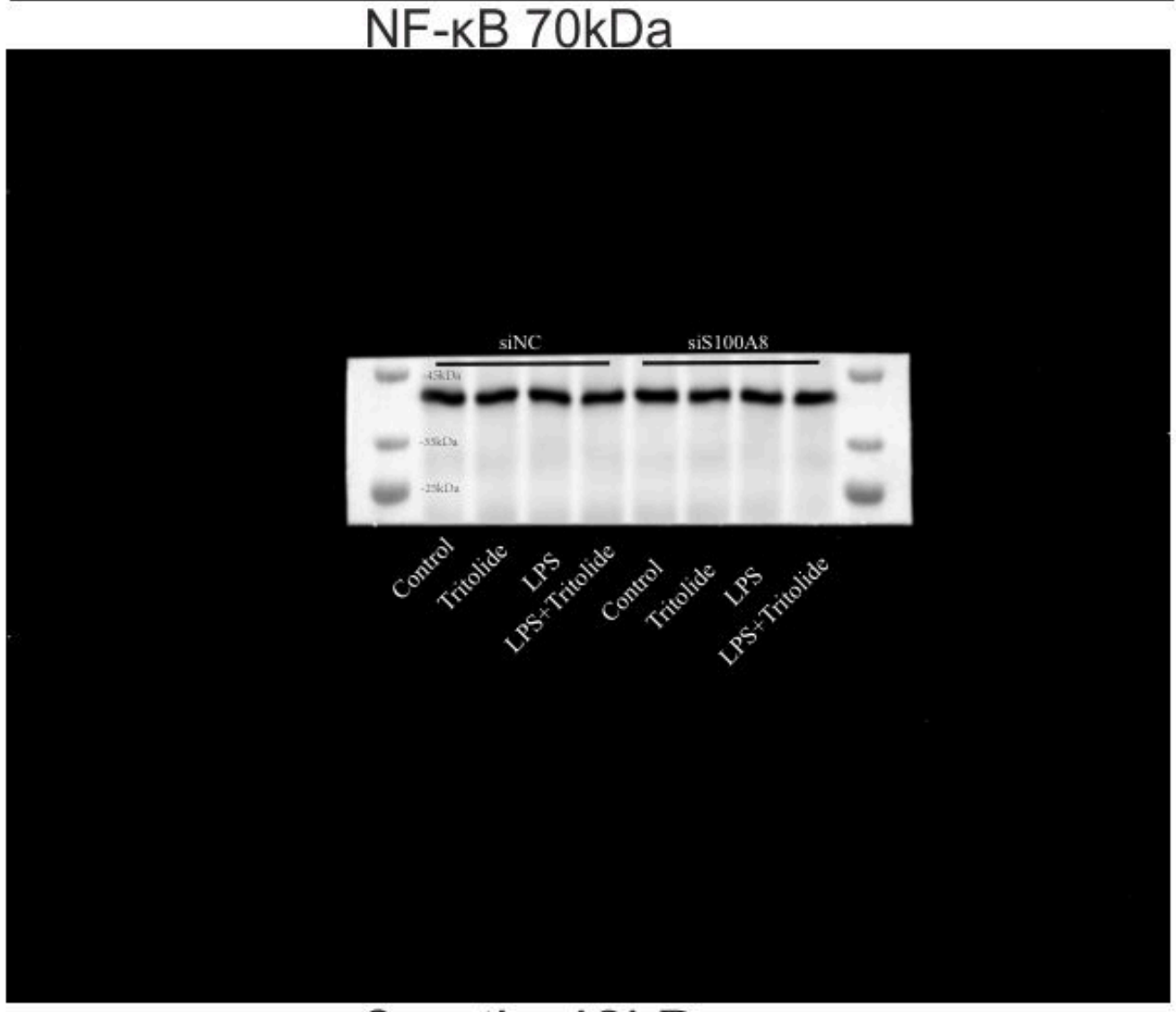

β-actin 42kDa

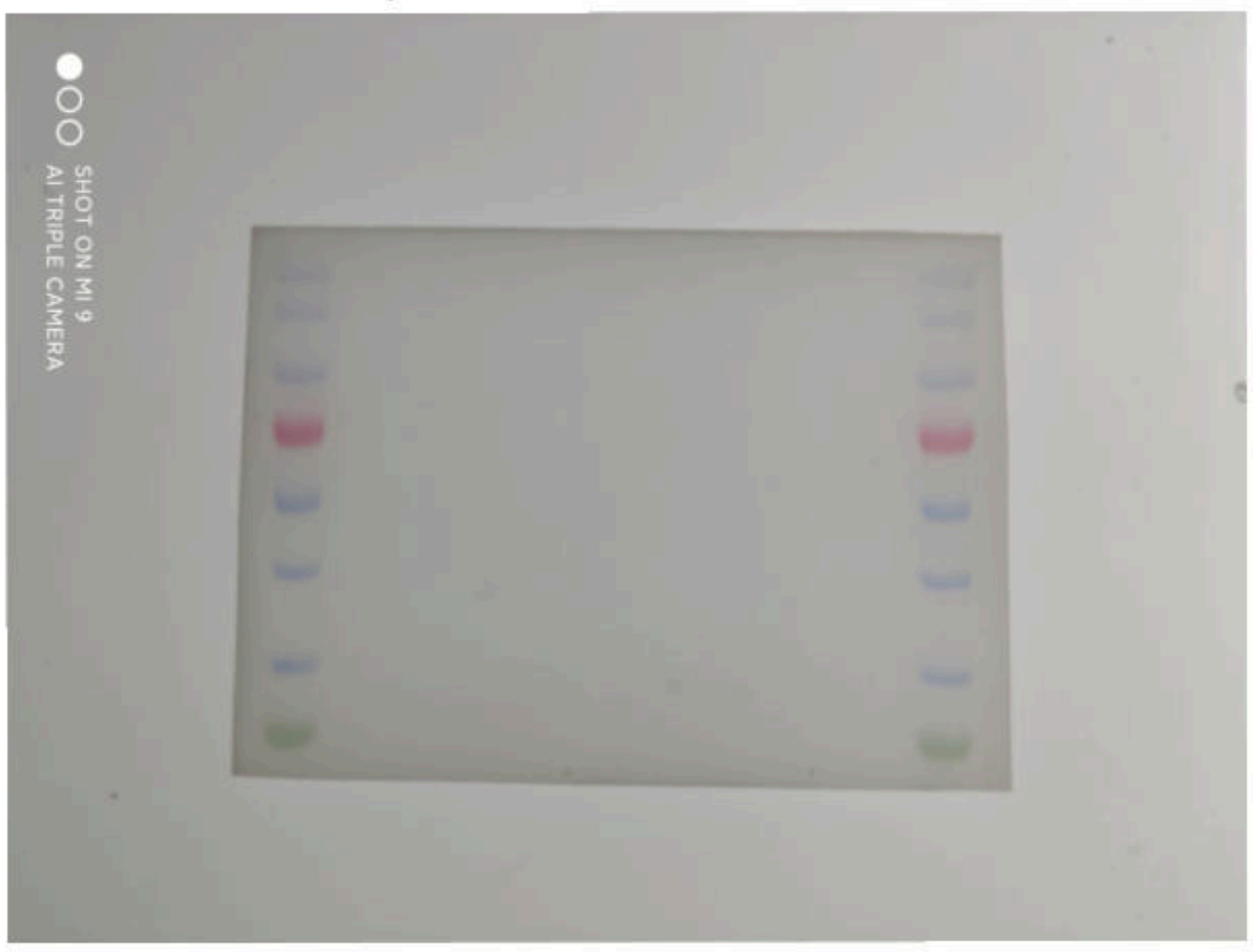

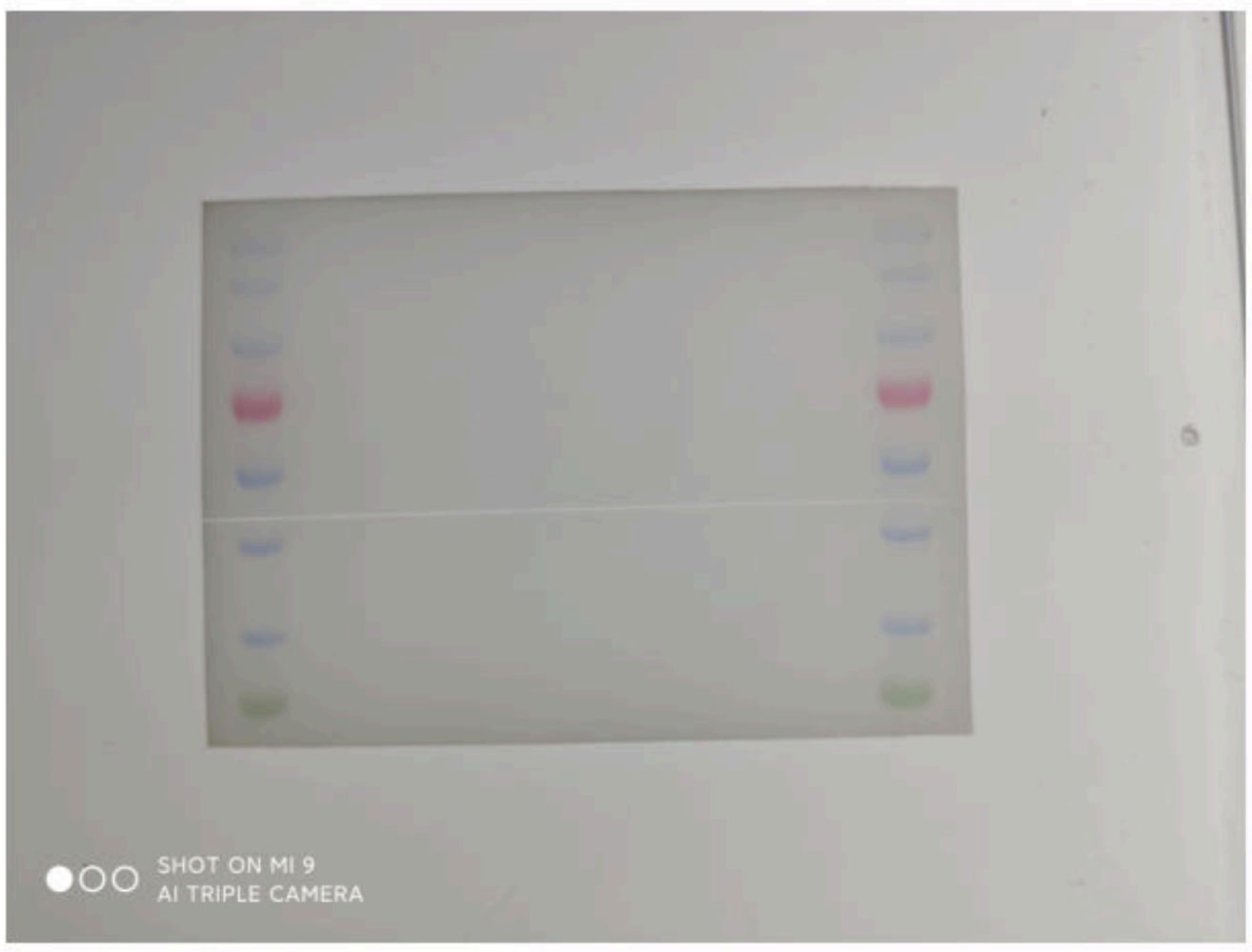

Source: New_validation_WB_source.pdf, original page 3. No brightness/contrast/gamma adjustment applied during compilation.

Eight-condition S100A8/beta-actin blot - repeat 1

siNC and siS100A8 x Control, TPL, LPS, and LPS+TPL. Final mapping: Fig 8A and Fig 8C.

WB

repeat 1

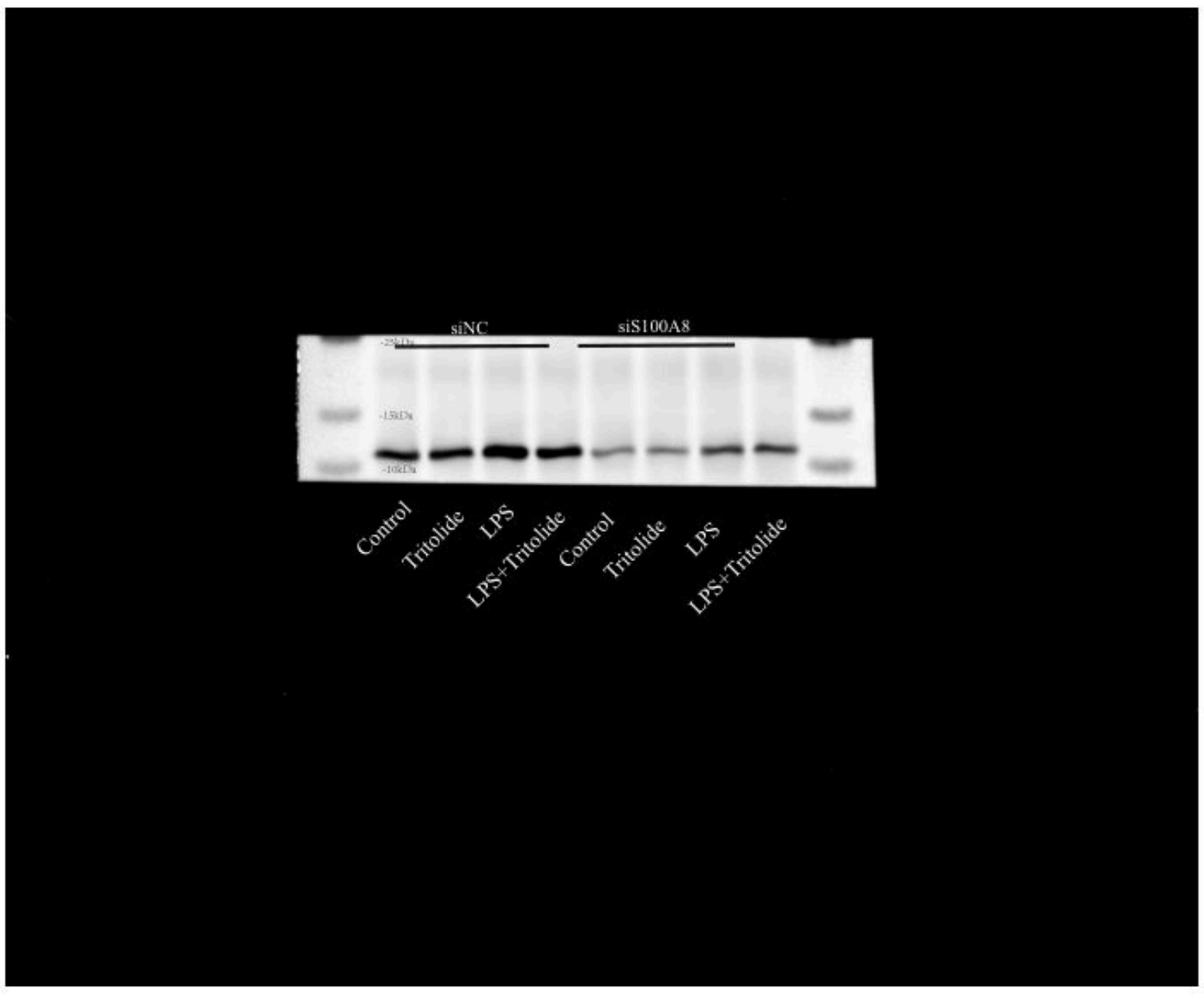

S100A8 11kDa

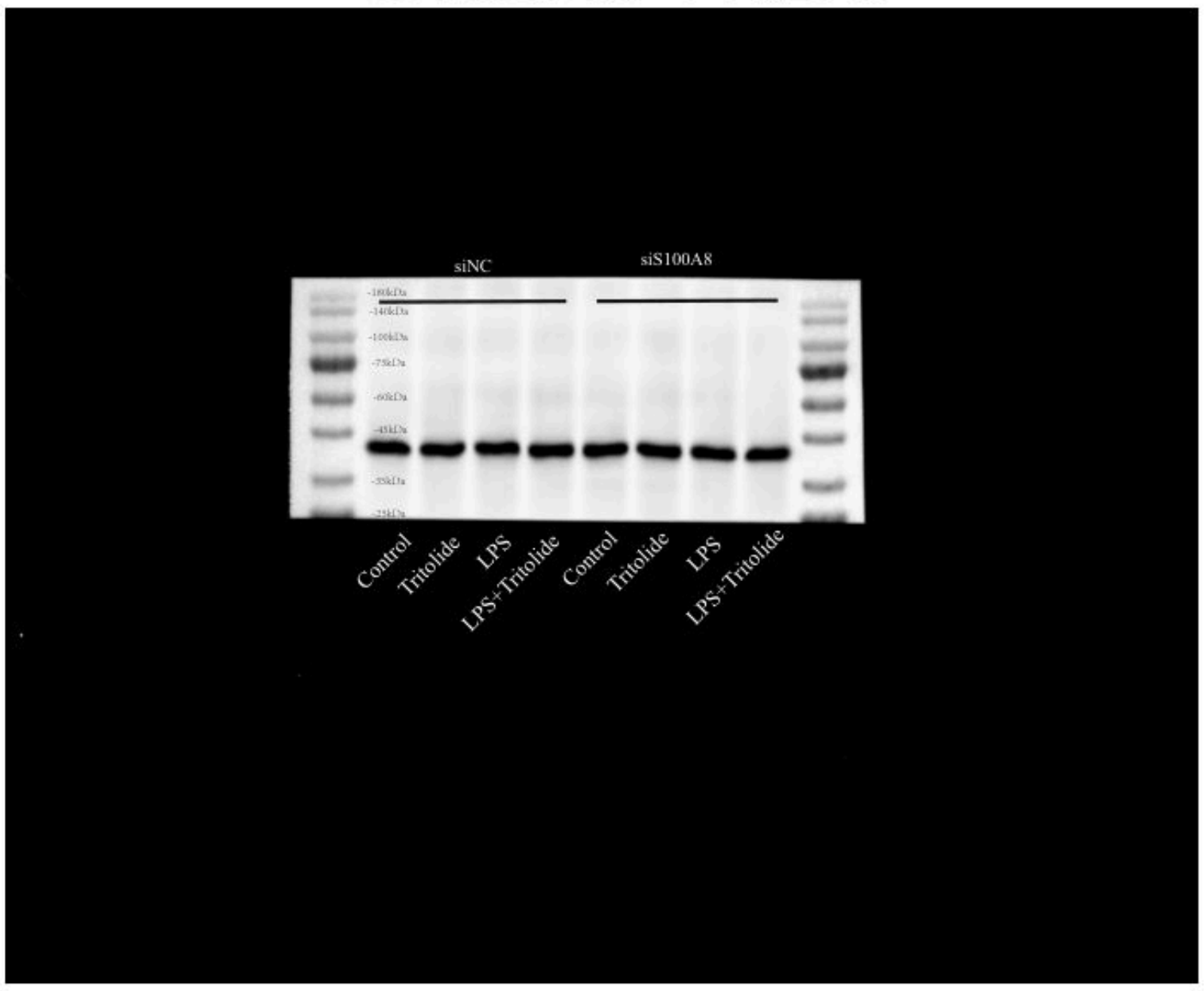

β-actin 42kDa

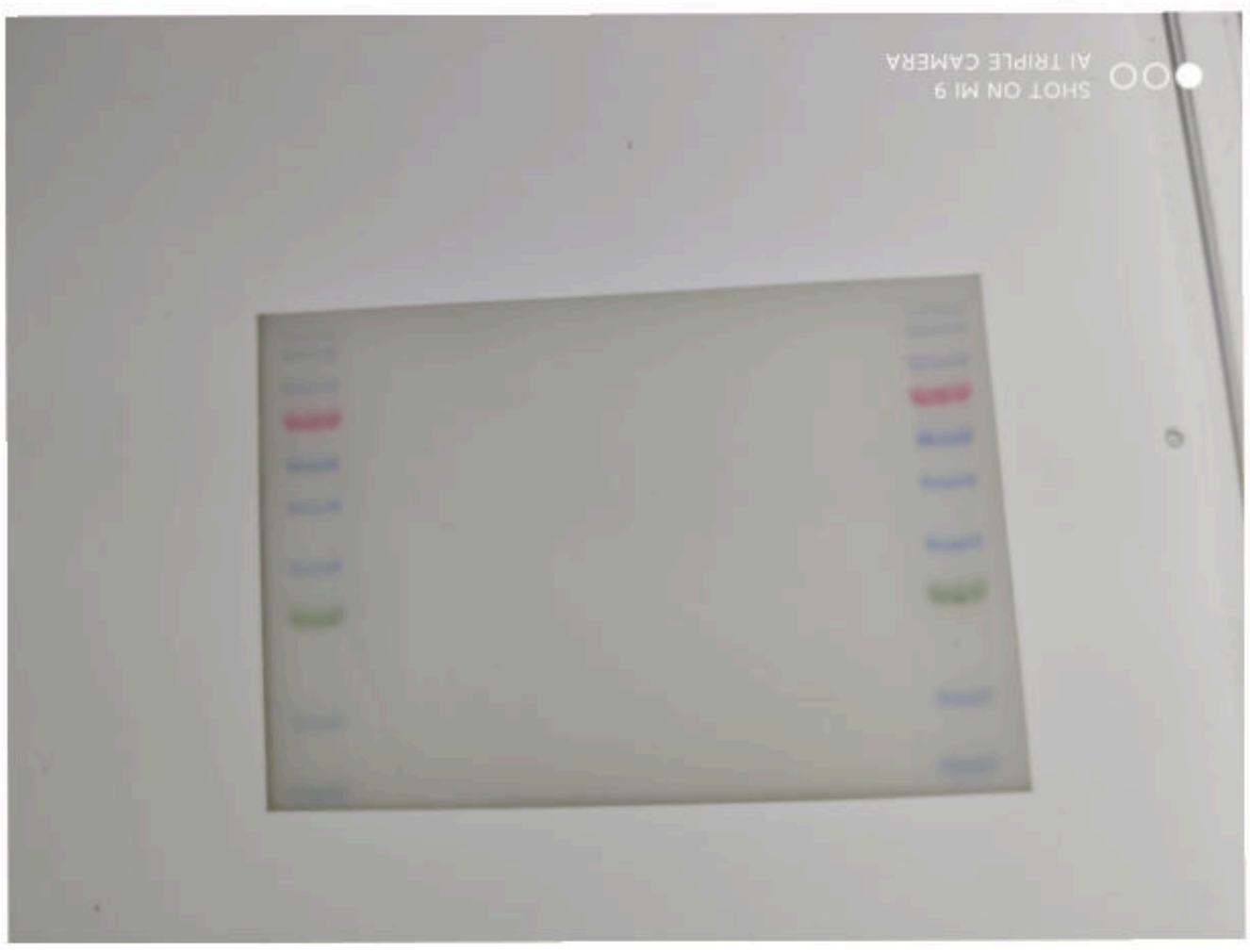

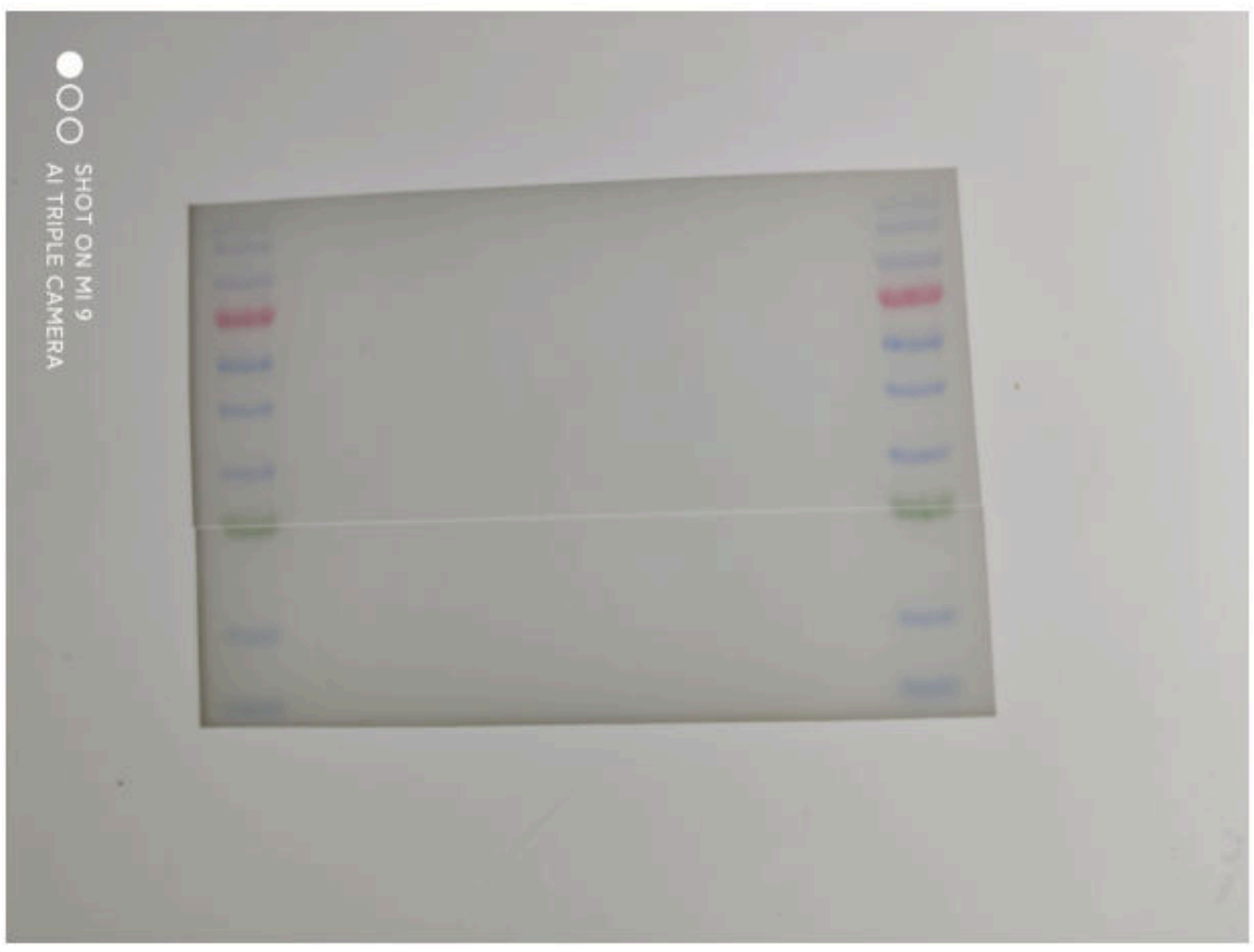

Source: New_validation_WB_source.pdf, original page 4. No brightness/contrast/gamma adjustment applied during compilation.

Eight-condition S100A8/beta-actin blot - repeat 2

siNC and siS100A8 x Control, TPL, LPS, and LPS+TPL. Final mapping: Fig 8A and Fig 8C.

WB

repeat 2

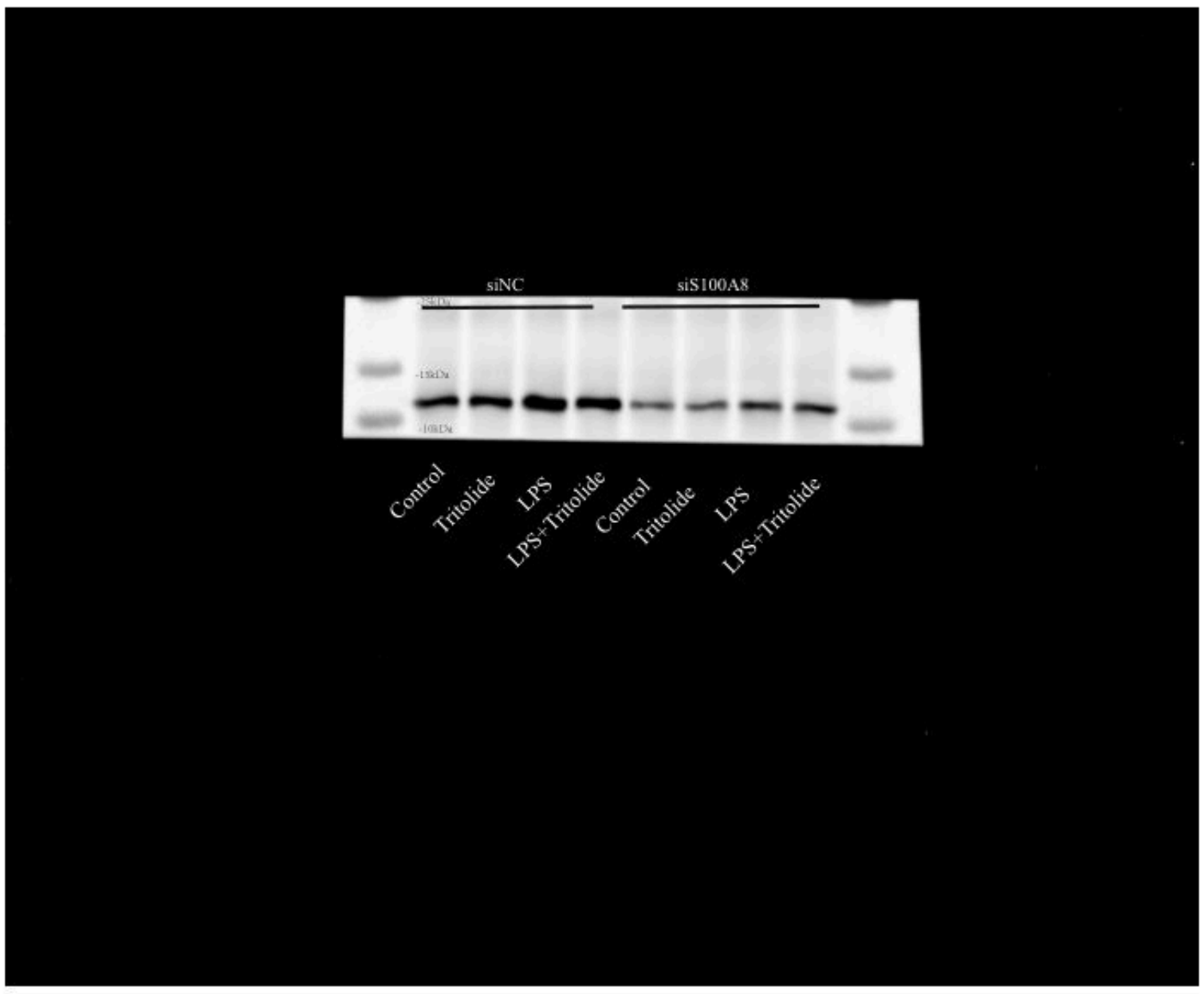

S100A8 11kDa

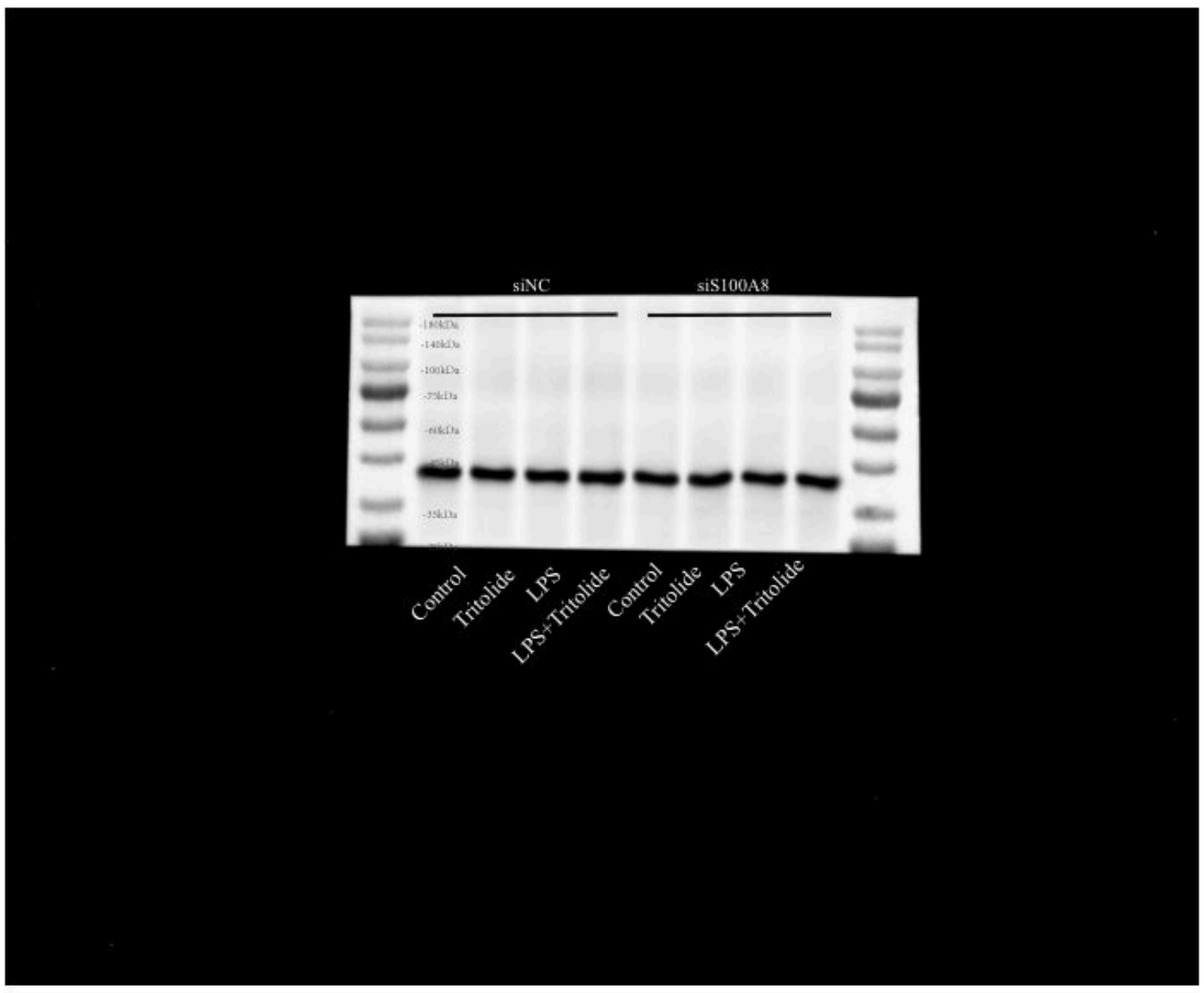

β-actin 42kDa

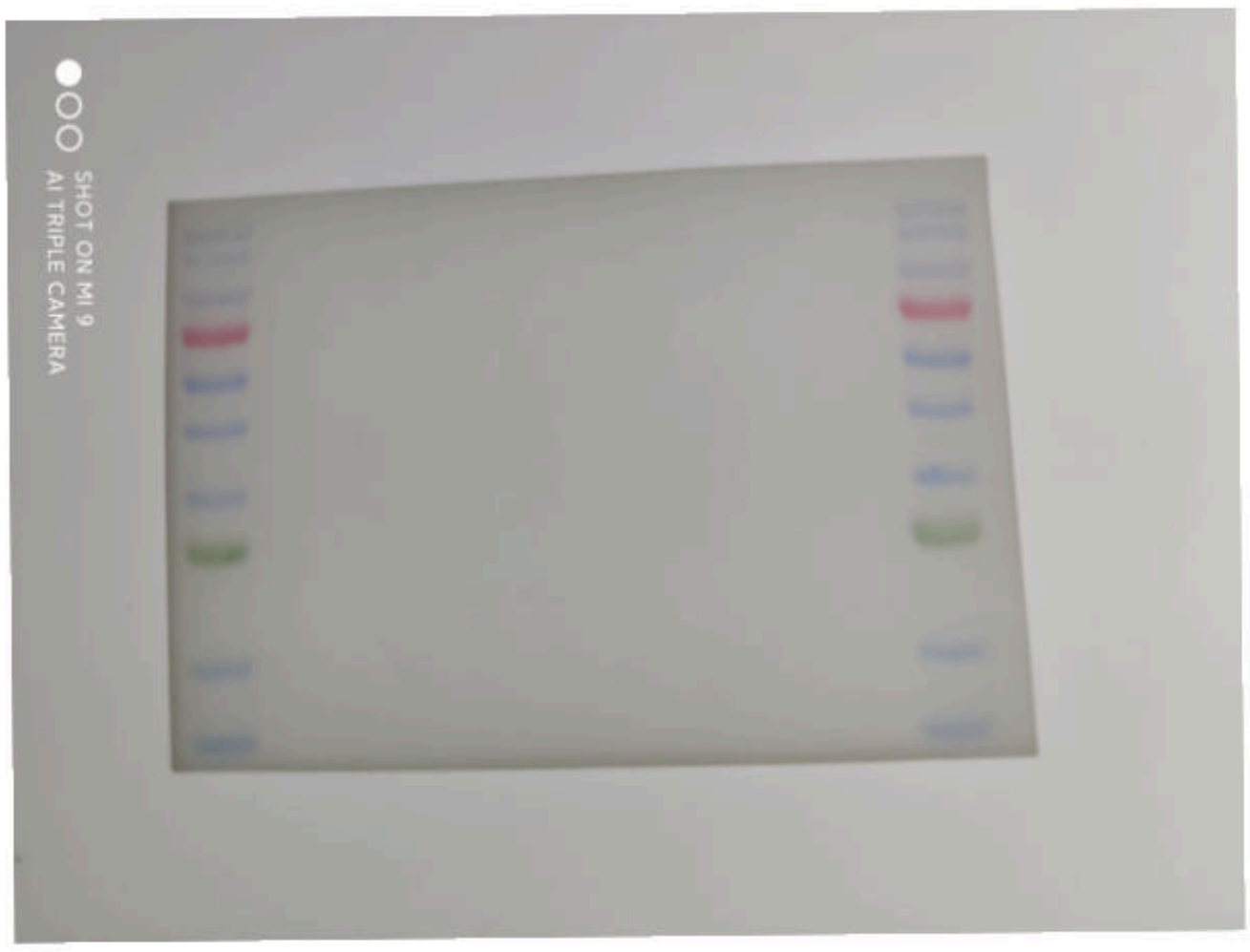

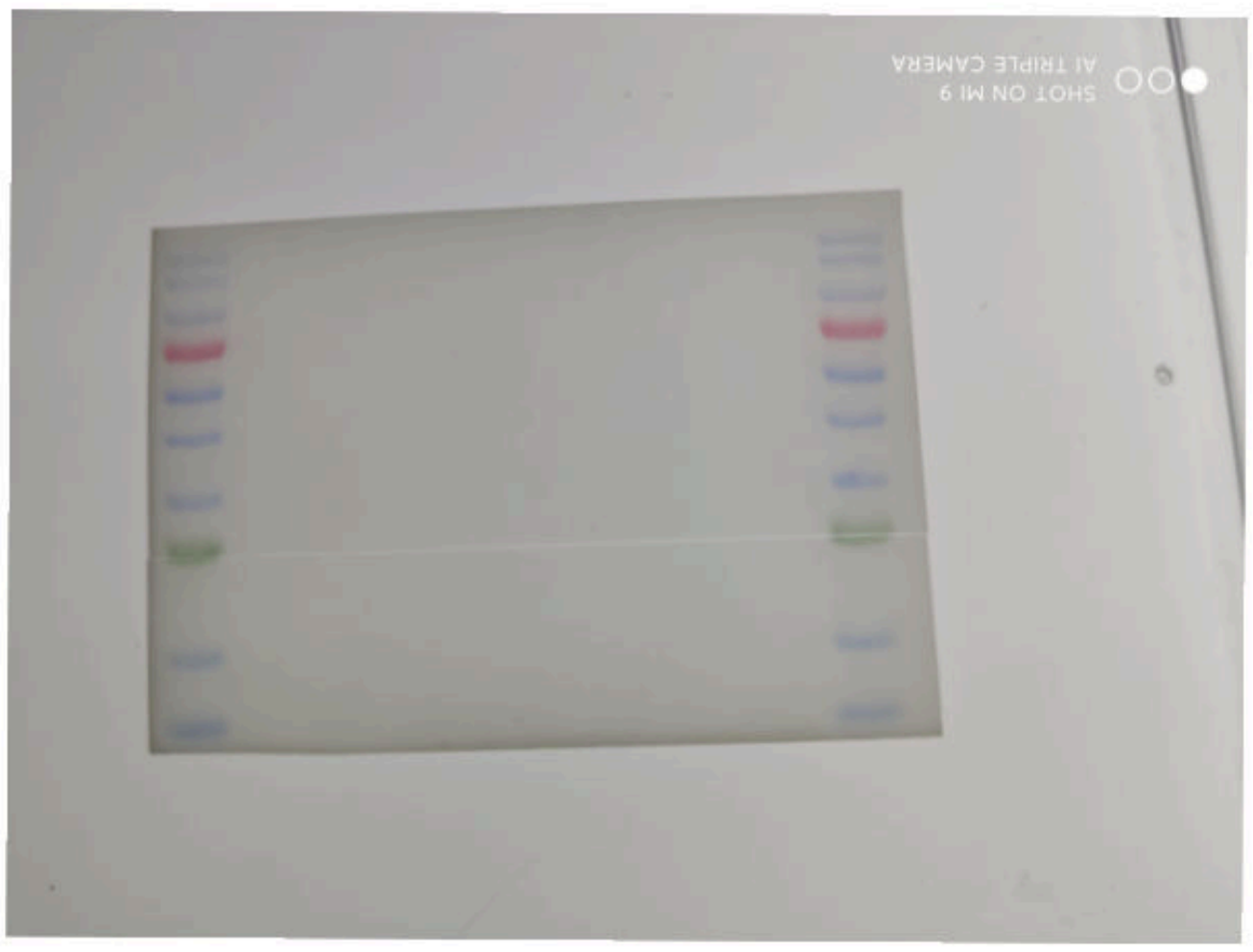

Source: New_validation_WB_source.pdf, original page 5. No brightness/contrast/gamma adjustment applied during compilation.

Eight-condition S100A8/beta-actin blot - repeat 3

siNC and siS100A8 x Control, TPL, LPS, and LPS+TPL. Final mapping: Fig 8A and Fig 8C.

WB

repeat 3

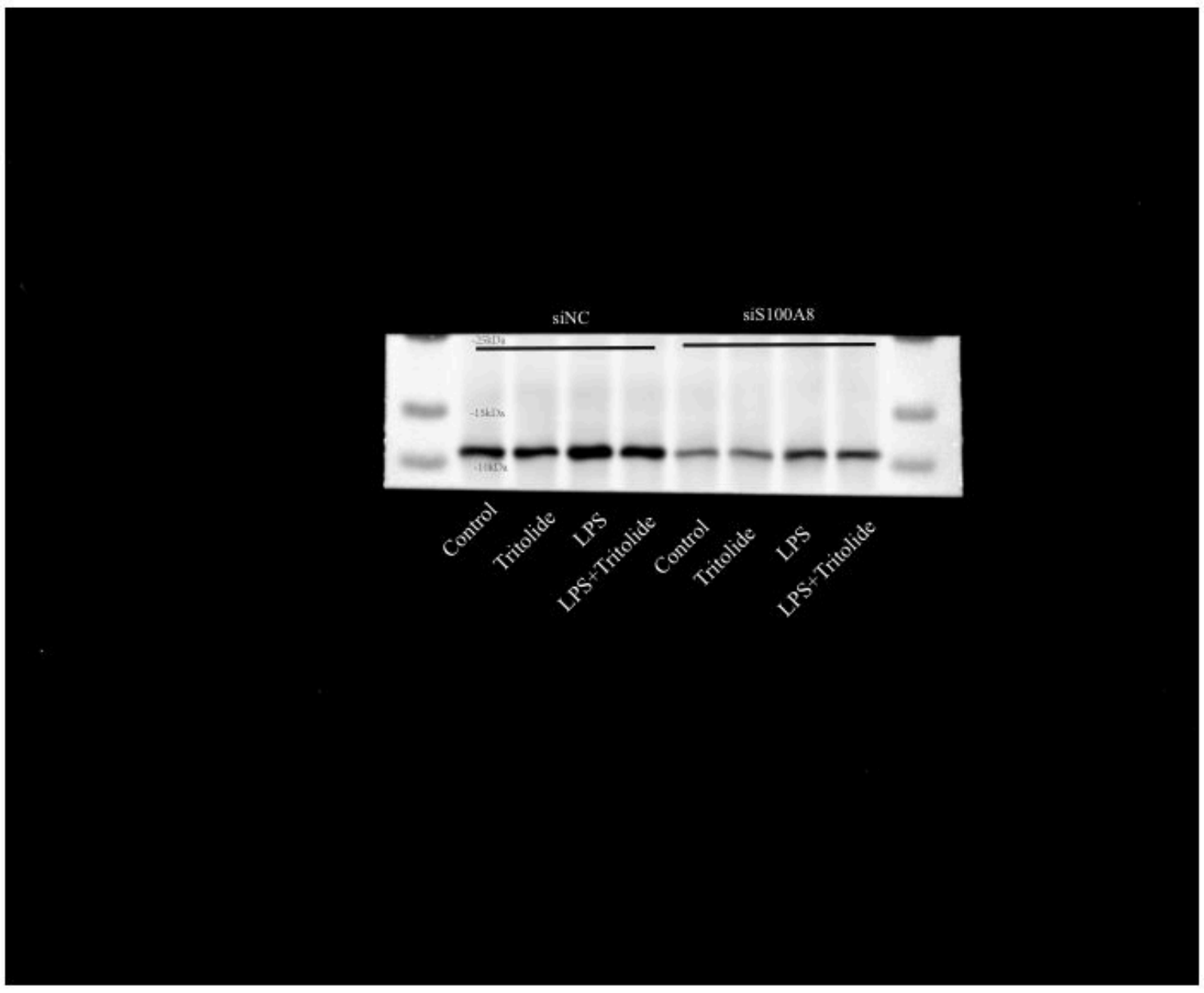

S100A8 11kDa

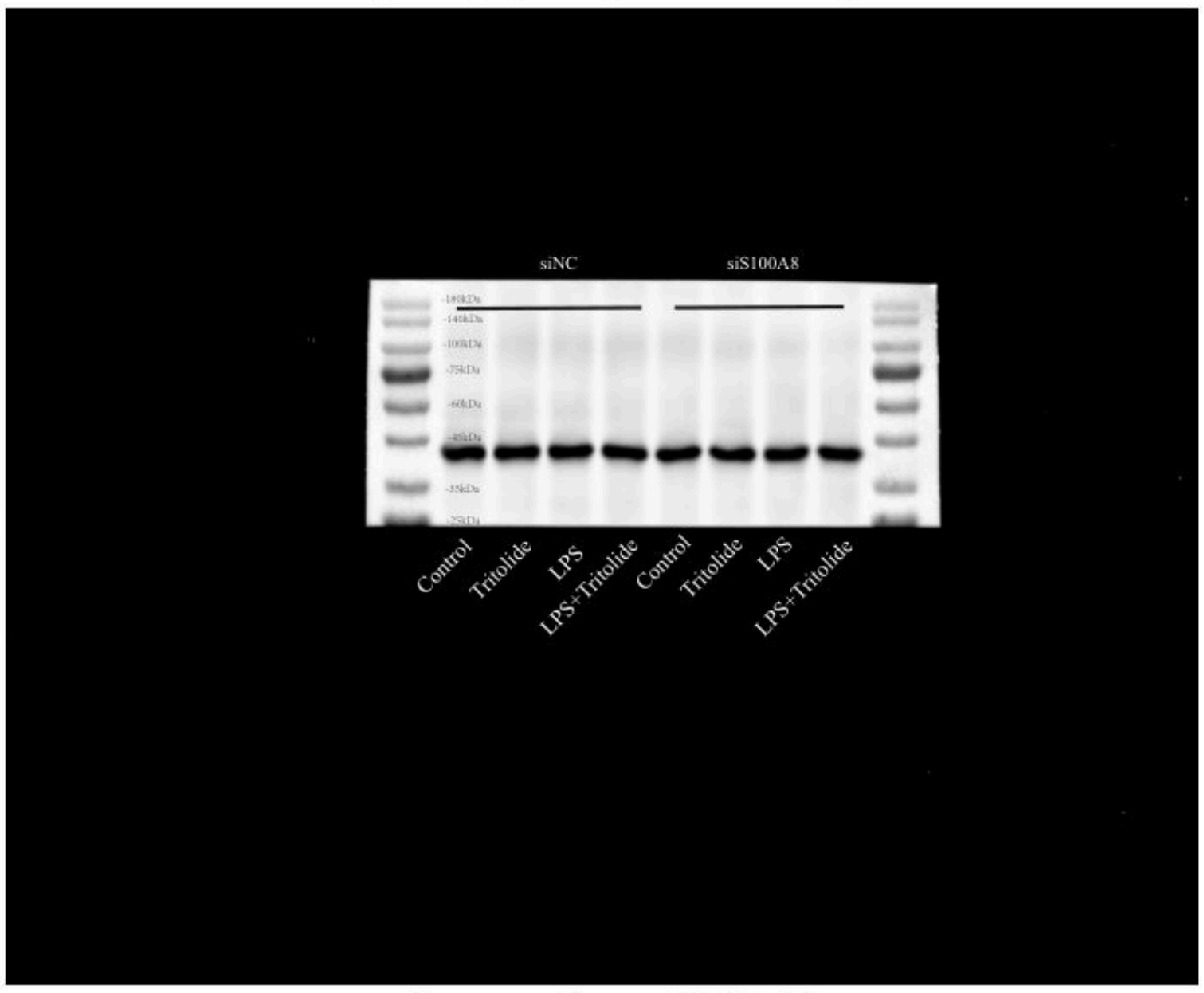

β-actin 42kDa

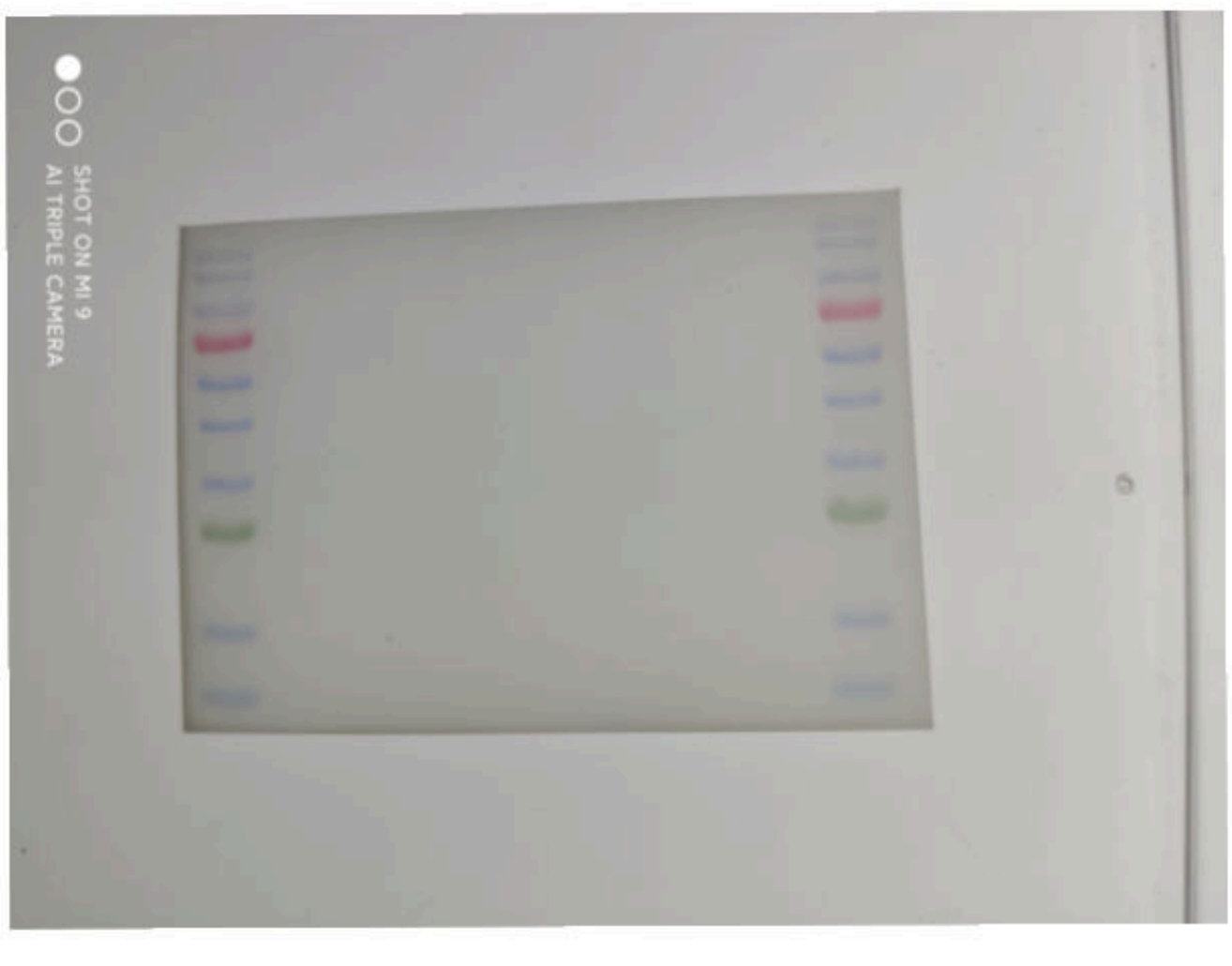

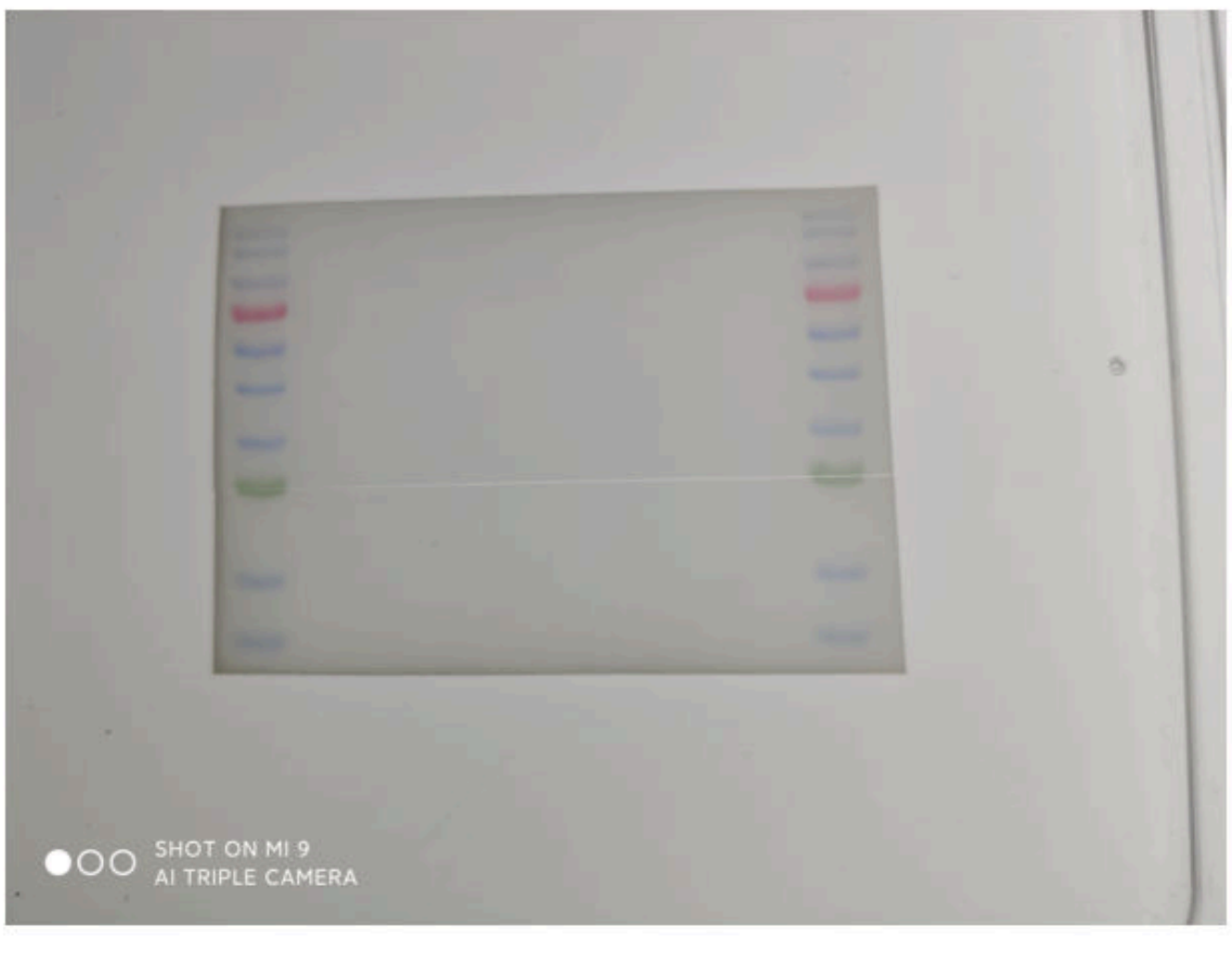

Source: New_validation_WB_source.pdf, original page 6. No brightness/contrast/gamma adjustment applied during compilation.

Four-condition CD74/HLA-DRalpha/S100A8/beta-actin blot - repeat 1

siNC-Control | siNC-LPS | siS100A8-Control | siS100A8-LPS. Final mapping: Fig 8D-G.

WB repeat 1

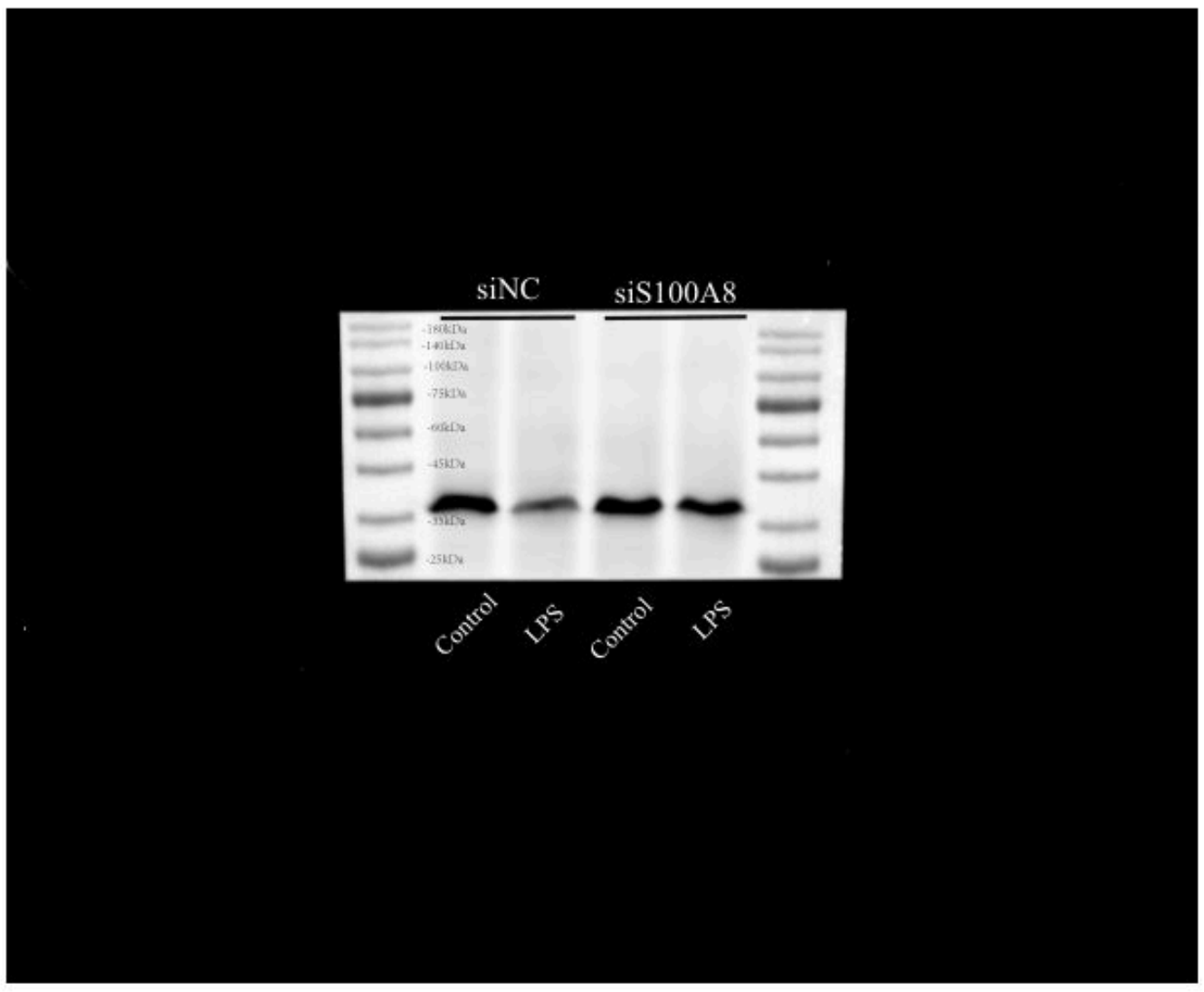

CD74 37kDa

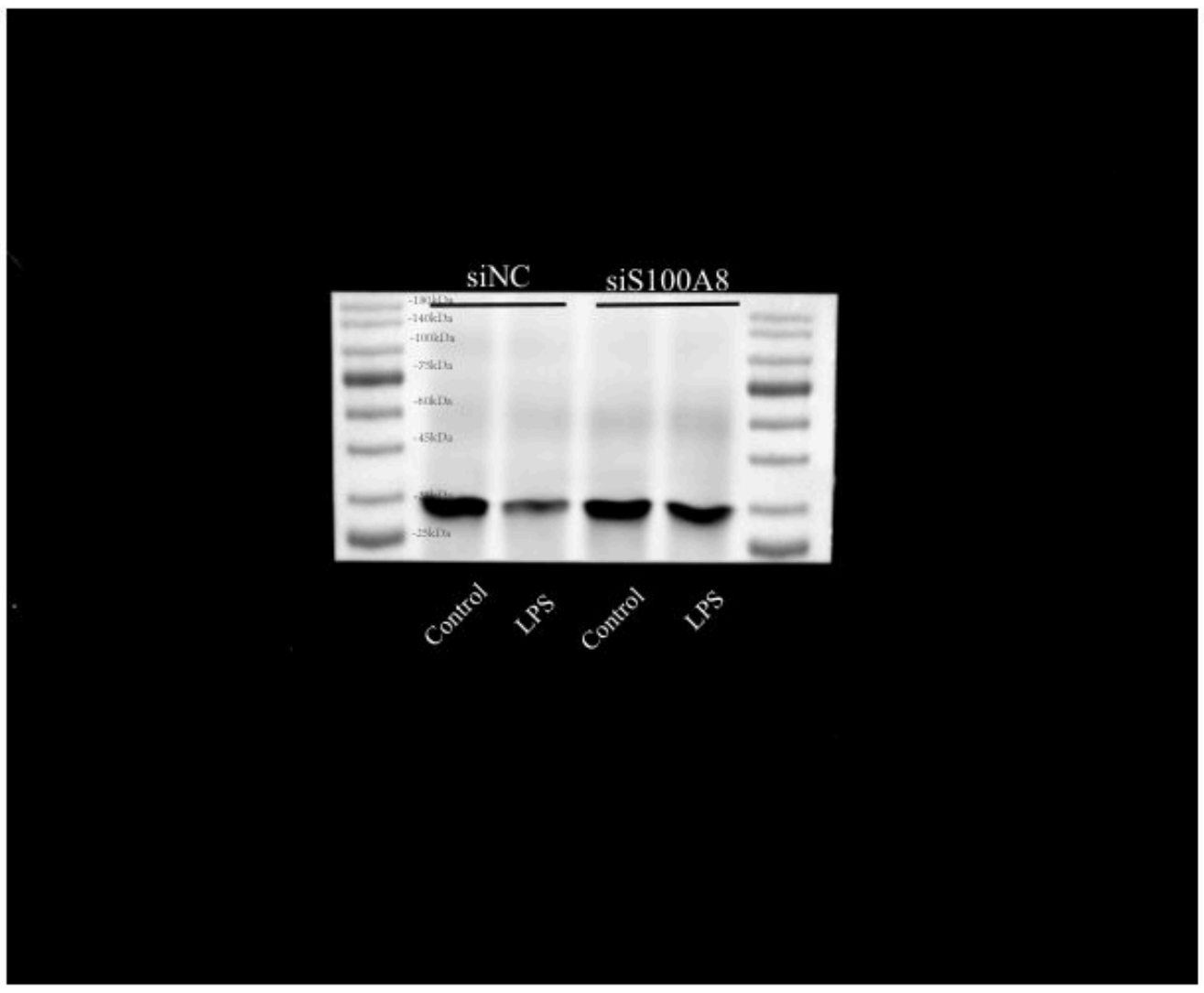

HLA-DRα 33kDa

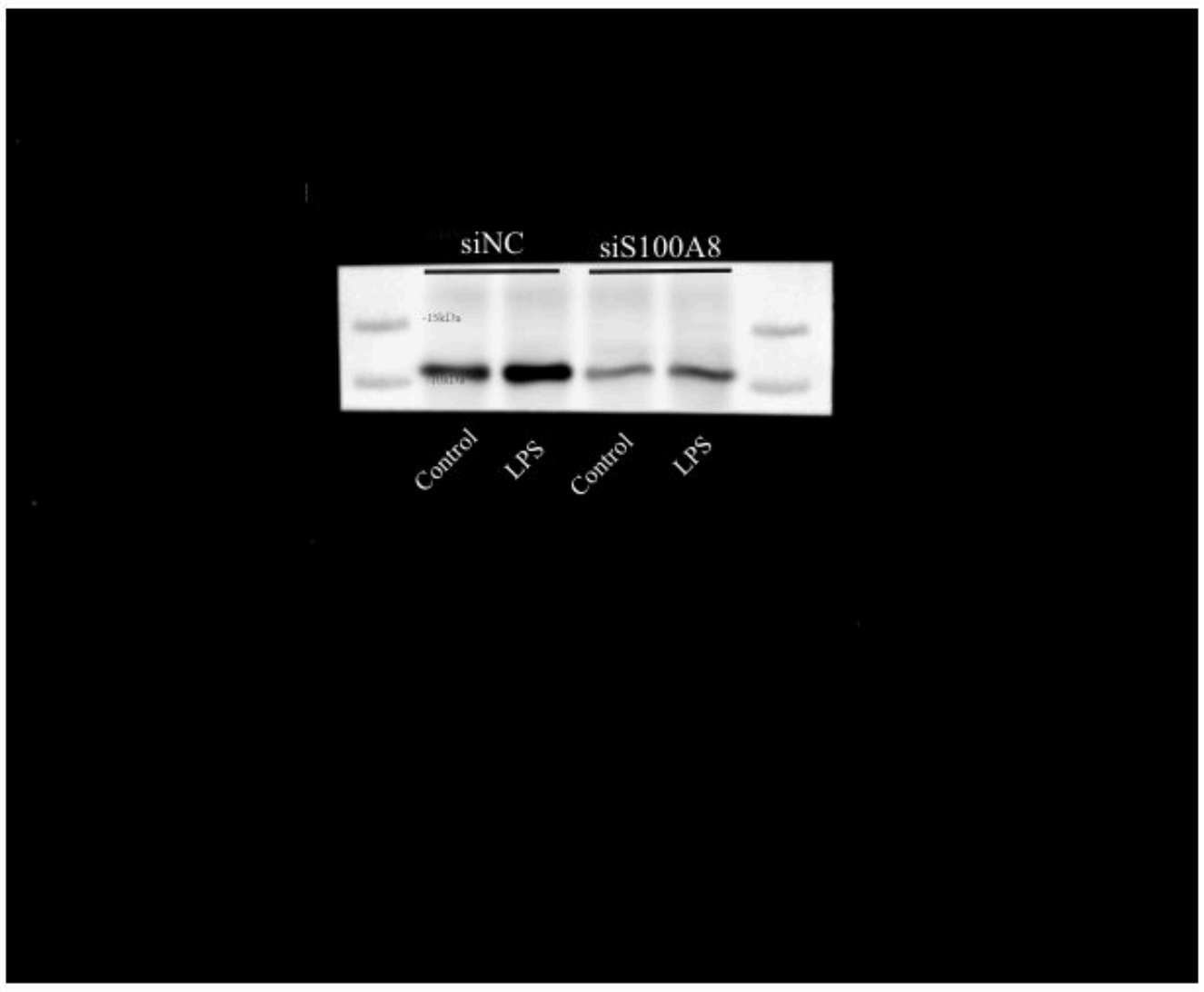

S100A8 11kDa

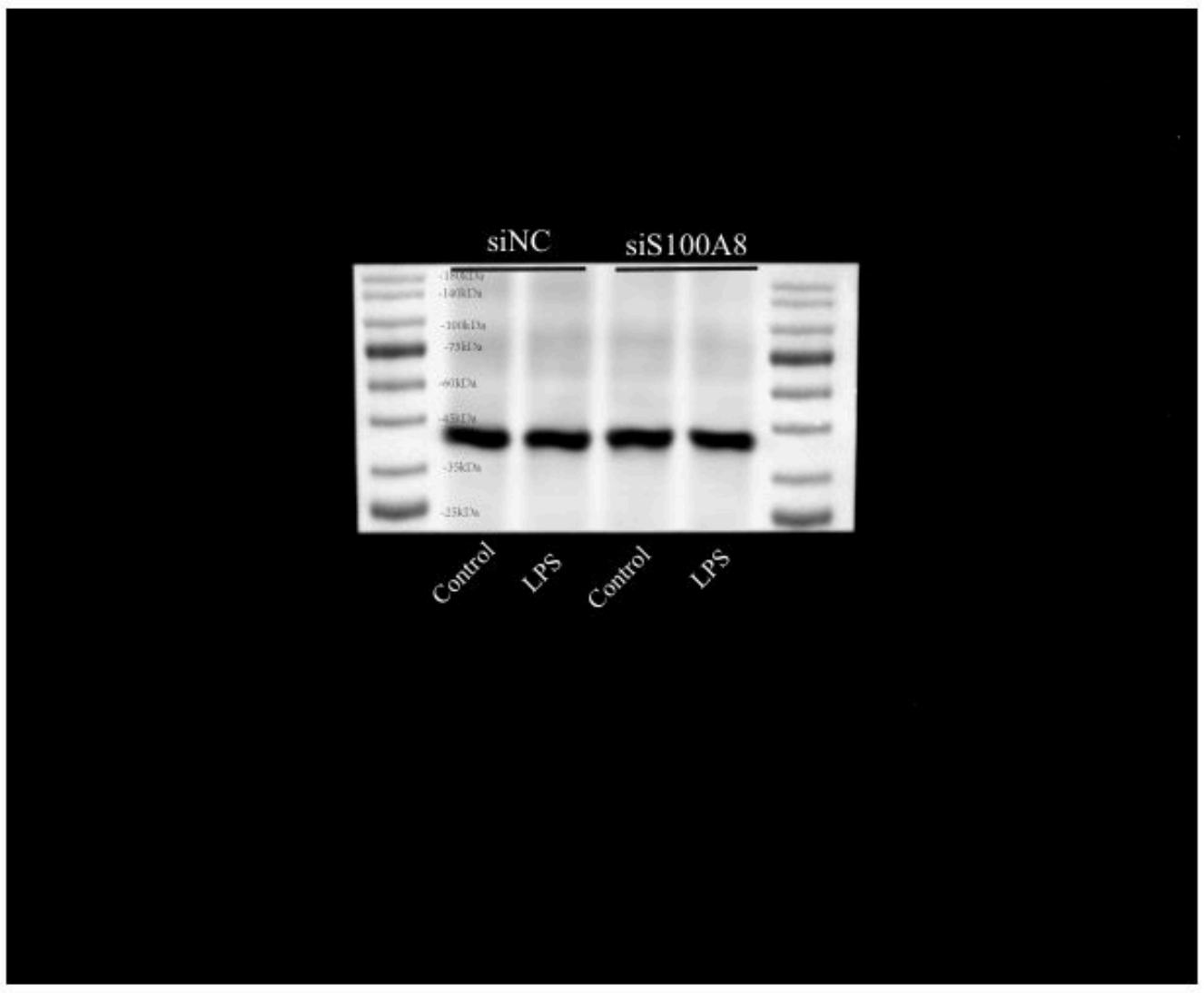

β-actin 42kDa

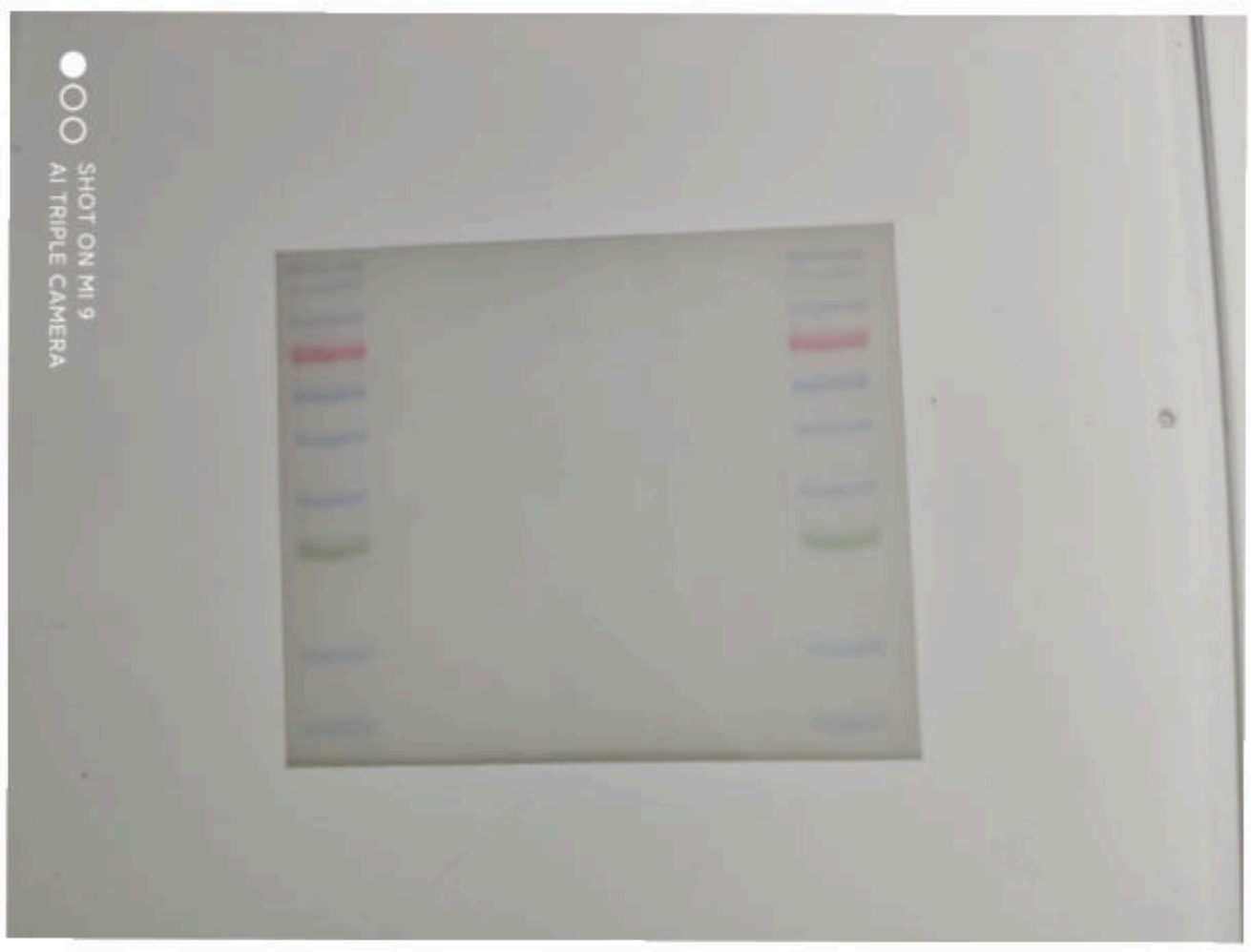

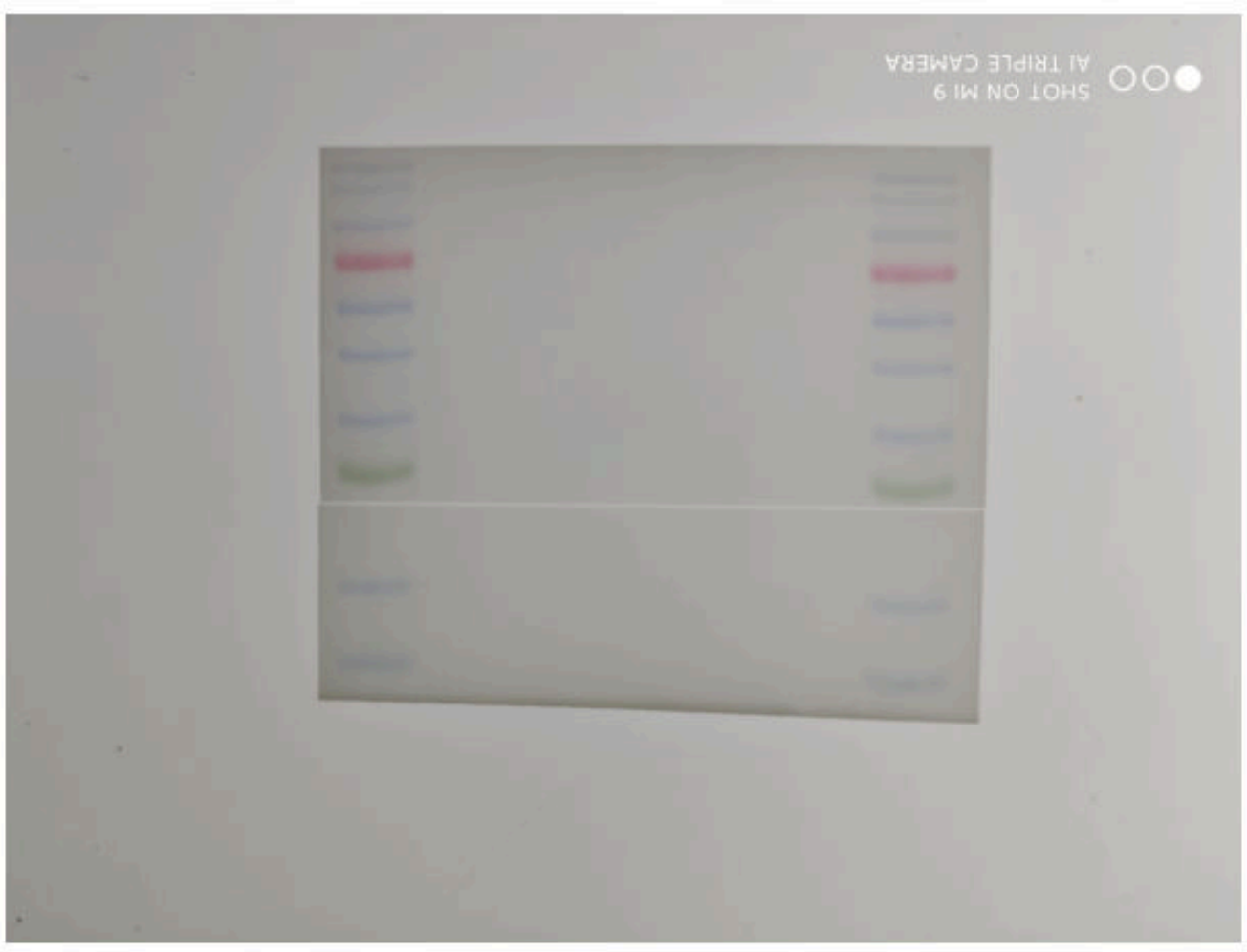

Source: New_validation_WB_source.pdf, original page 7. No brightness/contrast/gamma adjustment applied during compilation.

Four-condition CD74/HLA-DRalpha/S100A8/beta-actin blot - repeat 2

siNC-Control | siNC-LPS | siS100A8-Control | siS100A8-LPS. Final mapping: Fig 8D-G.

WB repeat 2

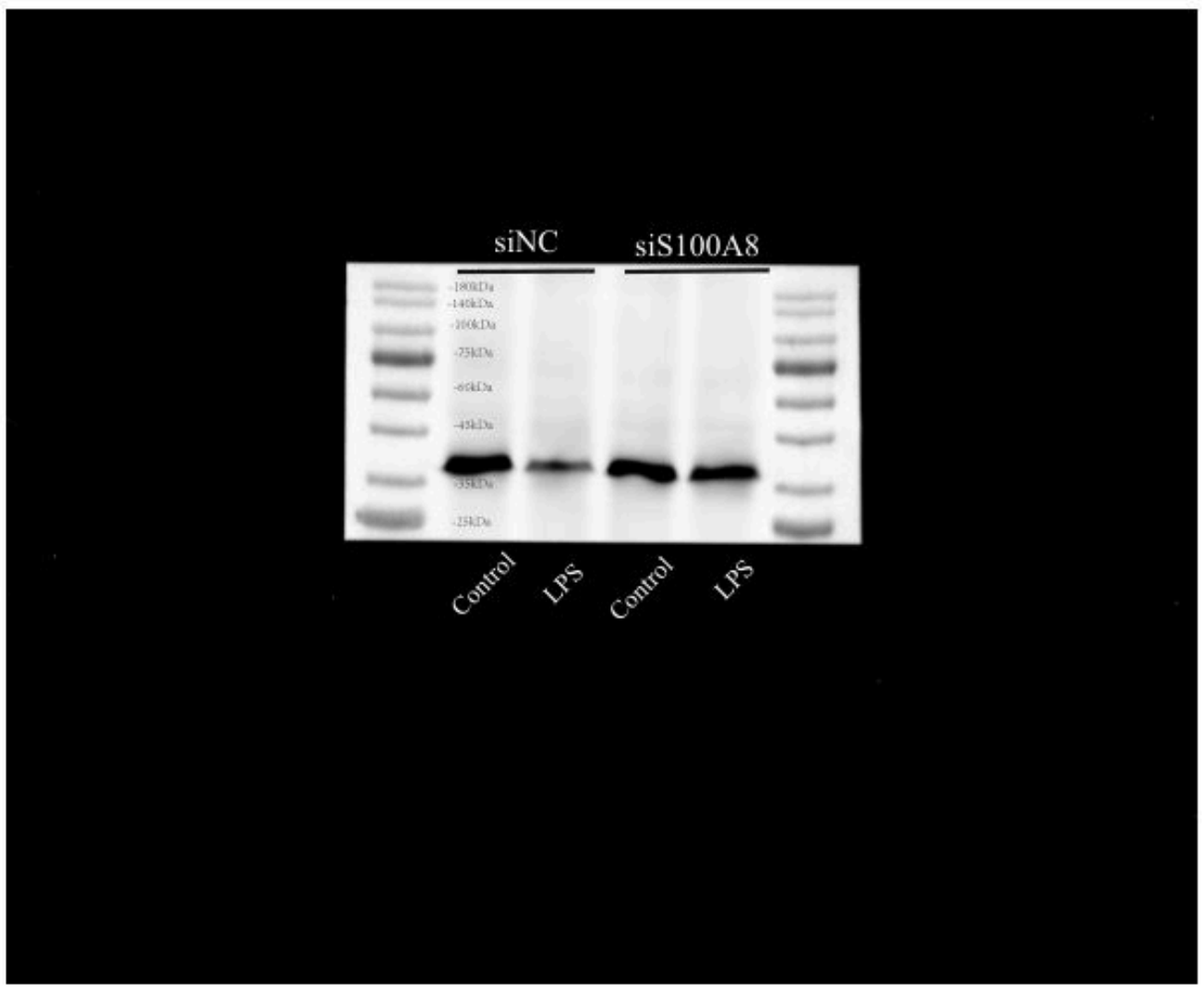

CD74 37kDa

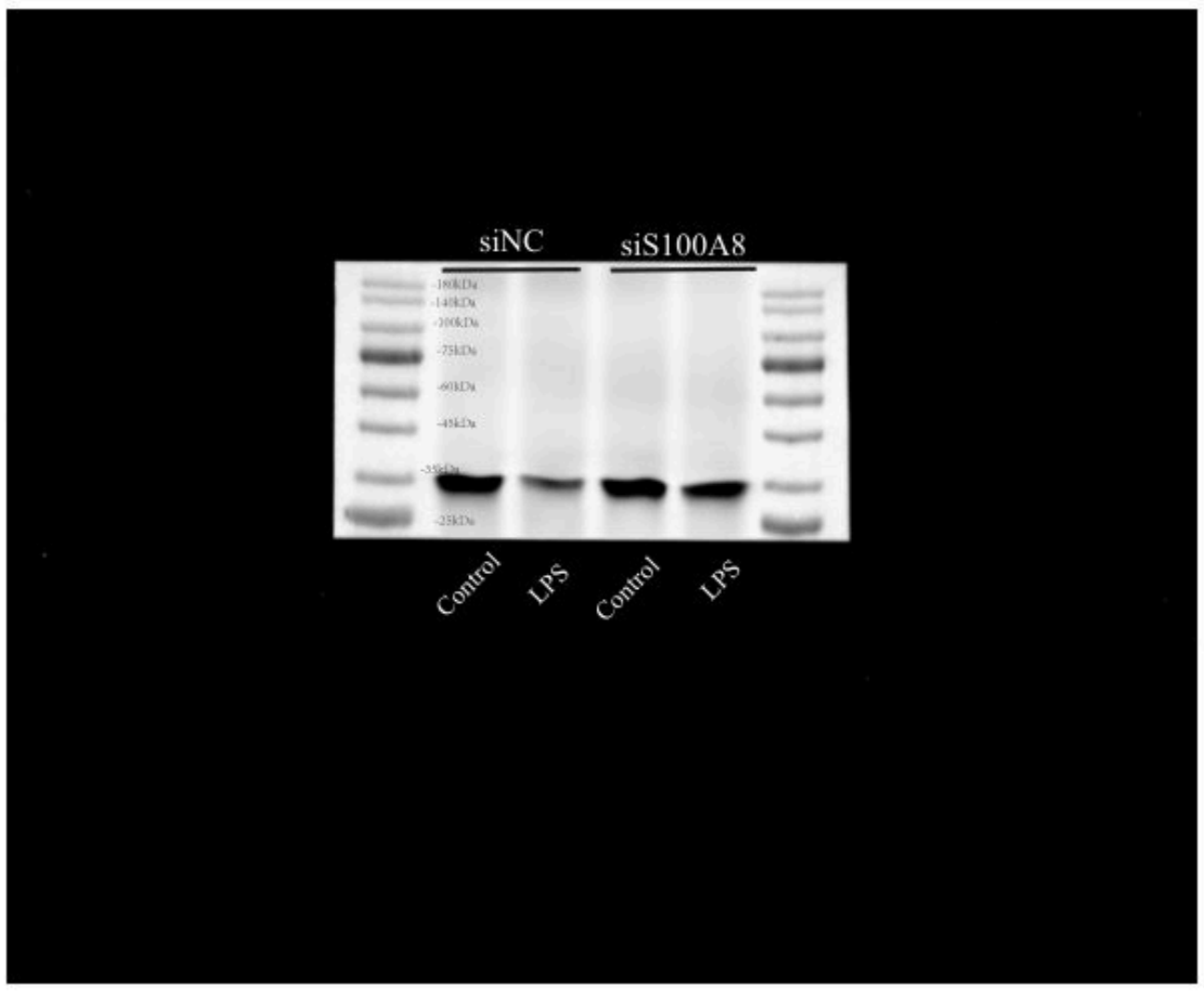

HLA-DRα 33kDa

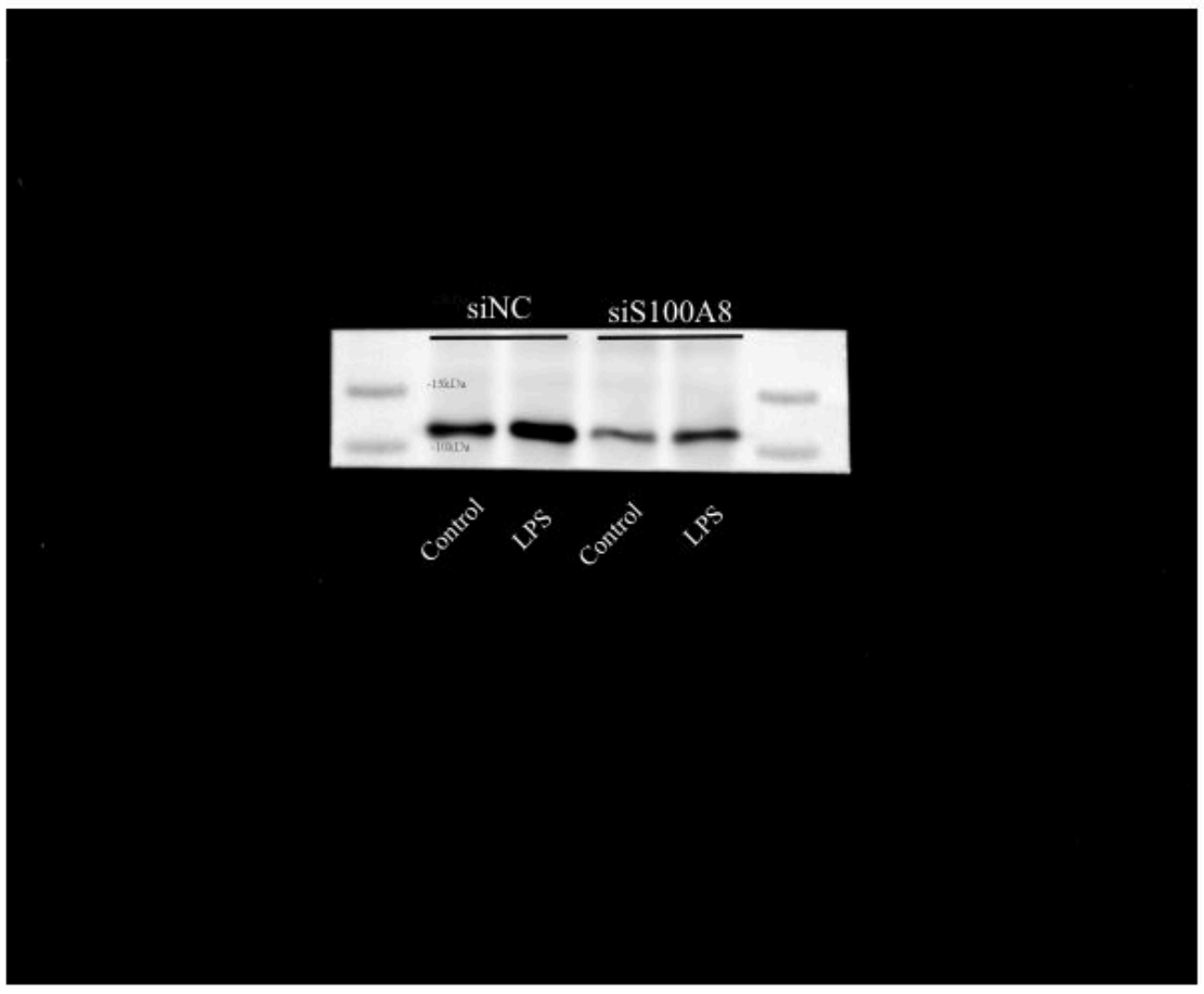

S100A8 11kDa

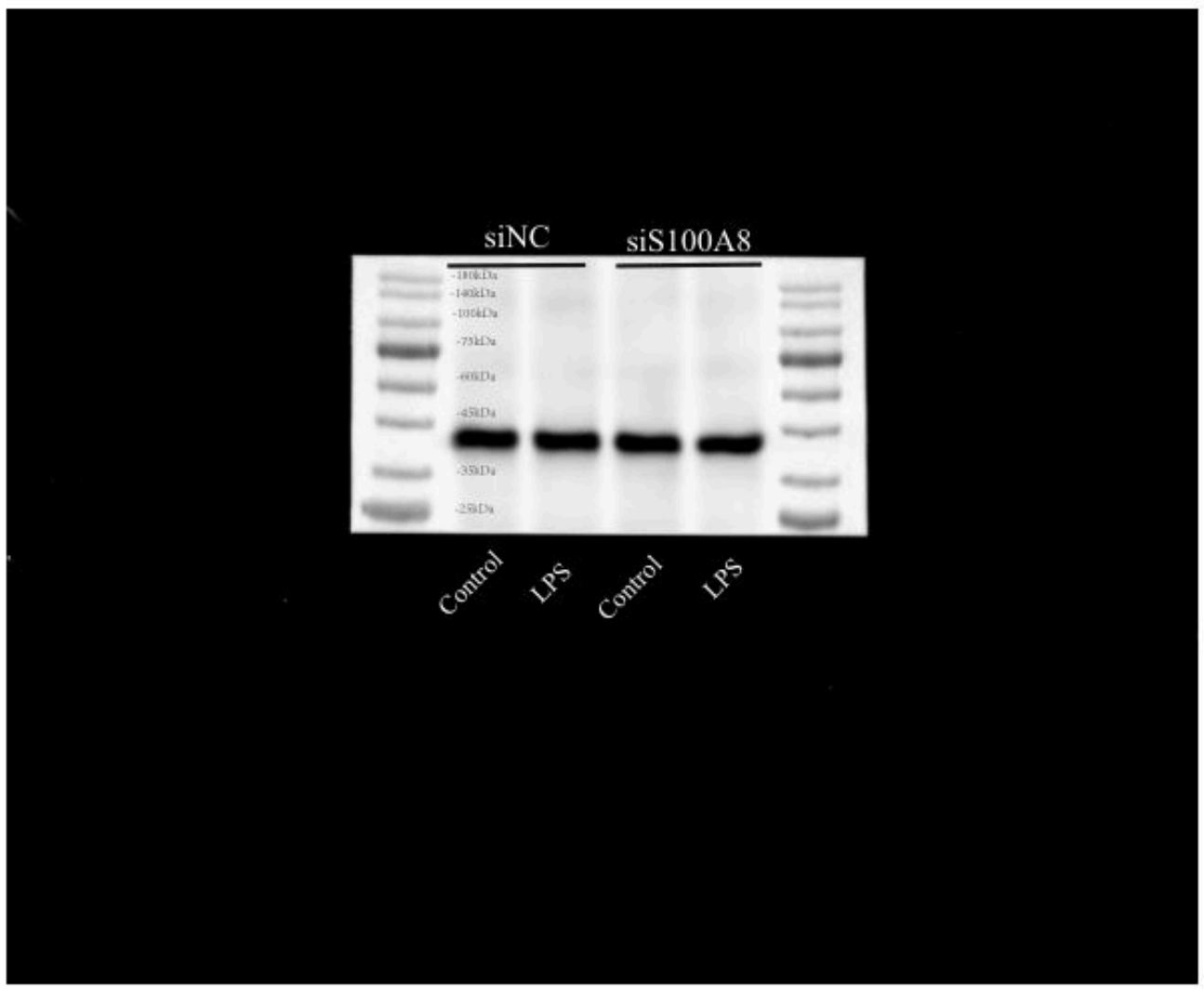

β-actin 42kDa

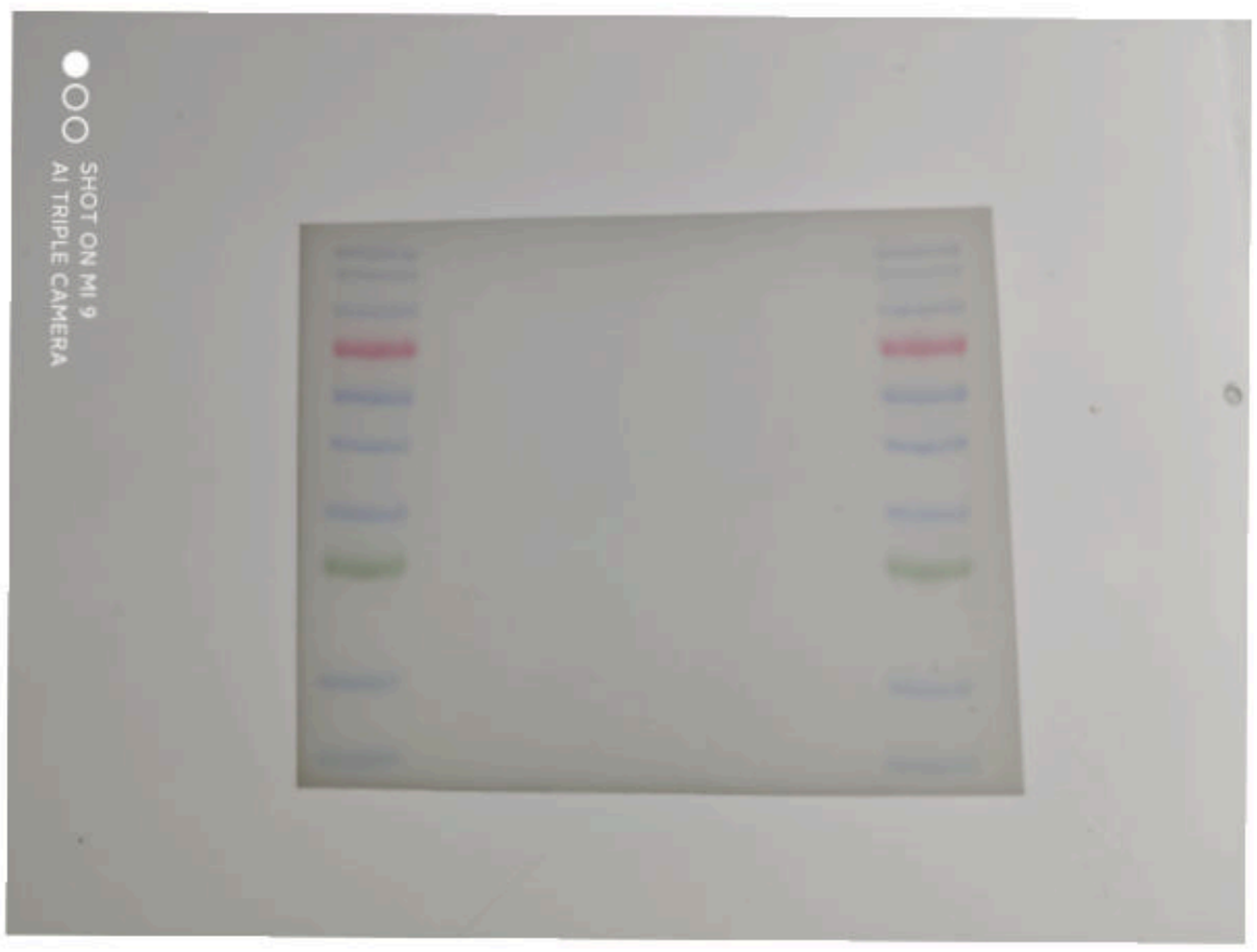

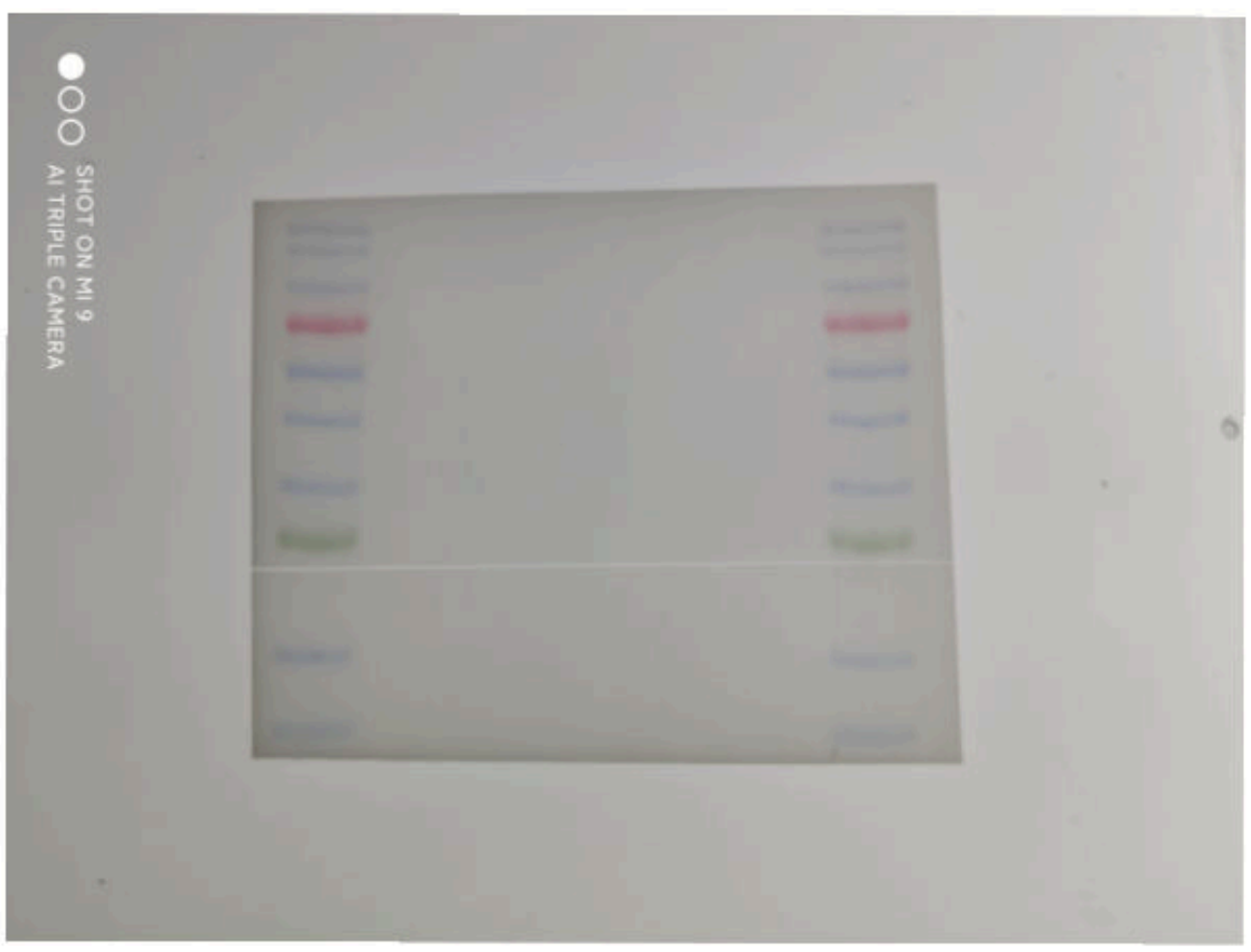

Source: New_validation_WB_source.pdf, original page 8. No brightness/contrast/gamma adjustment applied during compilation.

Four-condition CD74/HLA-DRalpha/S100A8/beta-actin blot - repeat 3

siNC-Control | siNC-LPS | siS100A8-Control | siS100A8-LPS. Final mapping: Fig 8D-G.

WB repeat 3

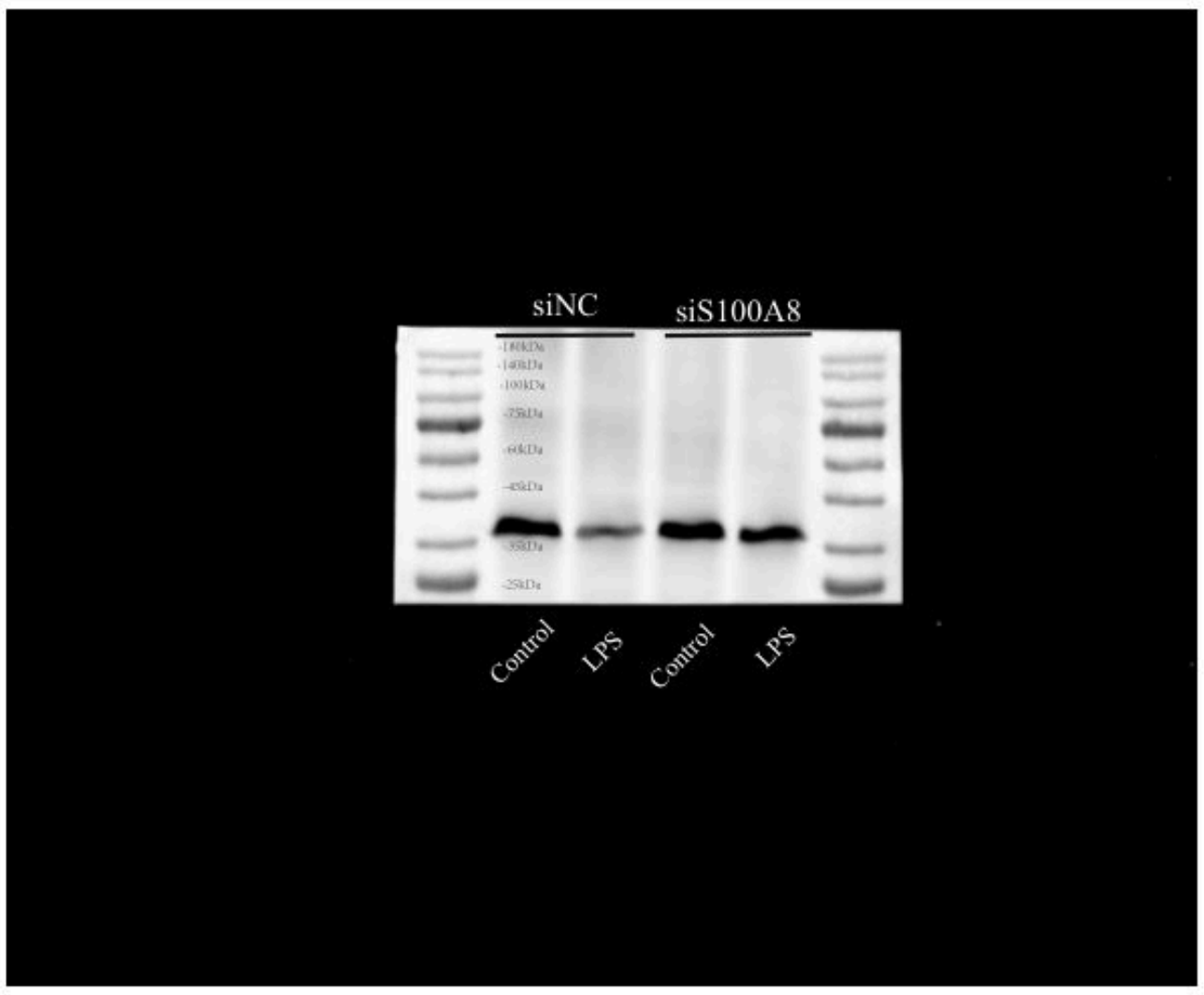

CD74 37kDa

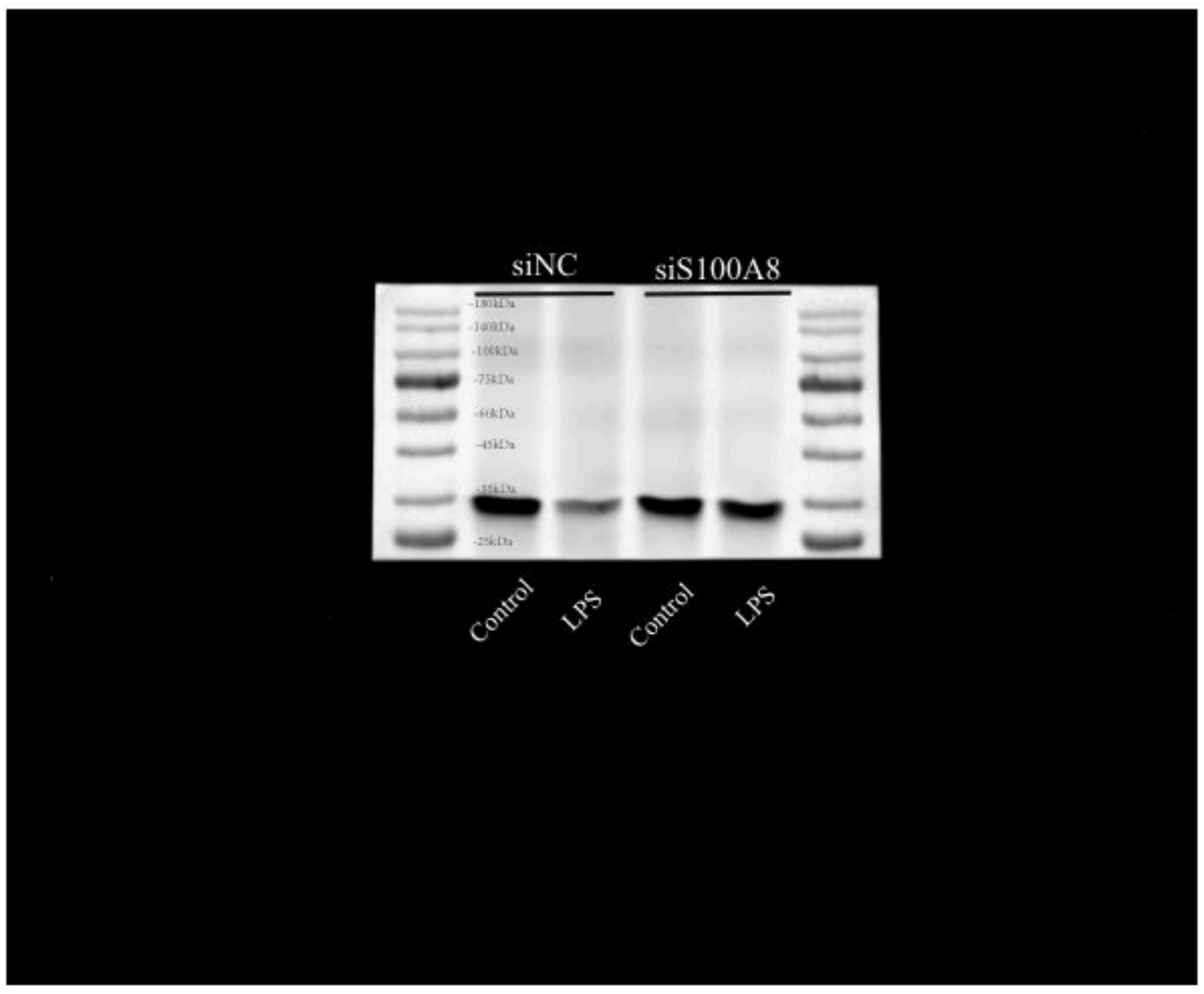

HLA-DRα 33kDa

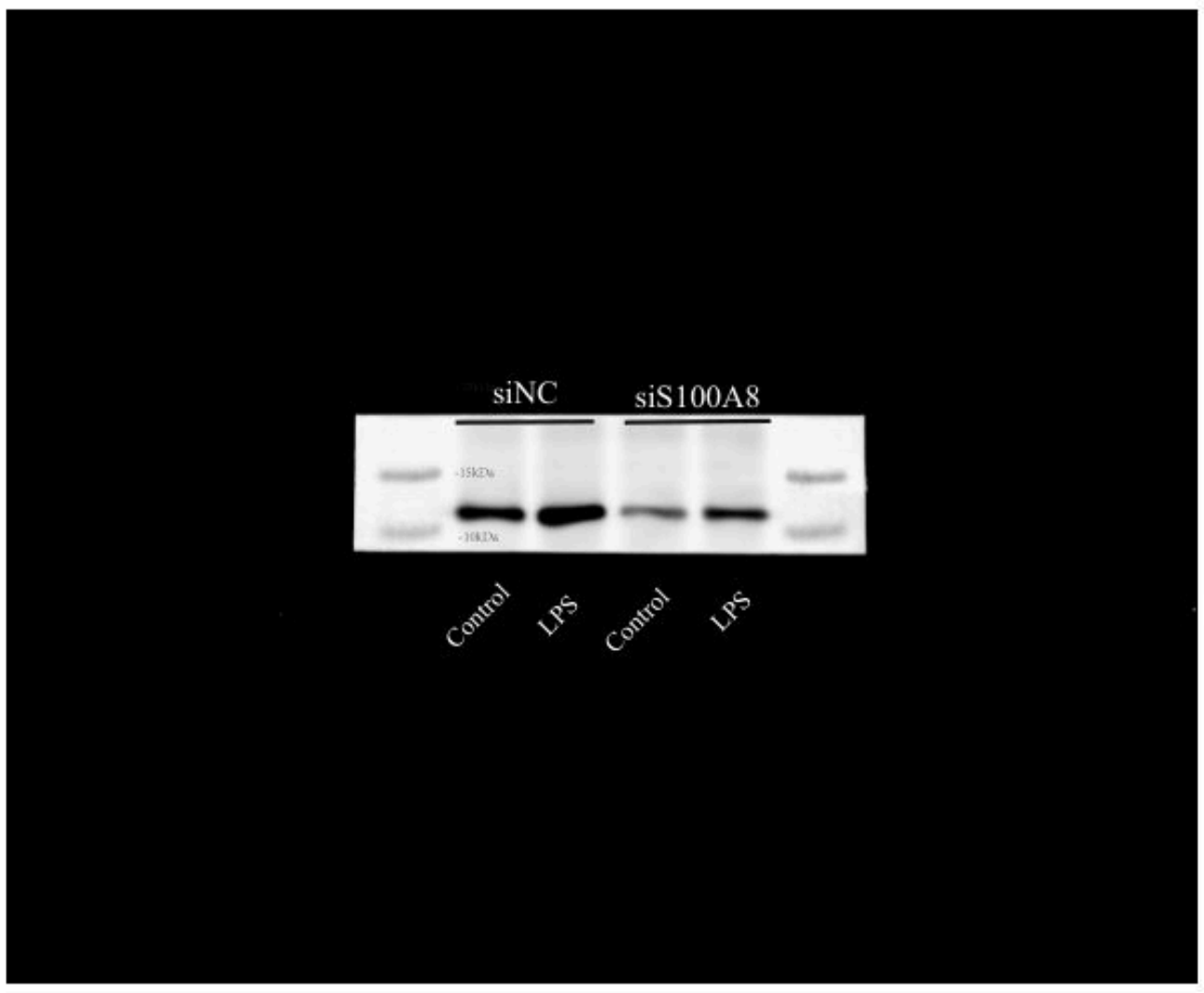

S100A8 11kDa

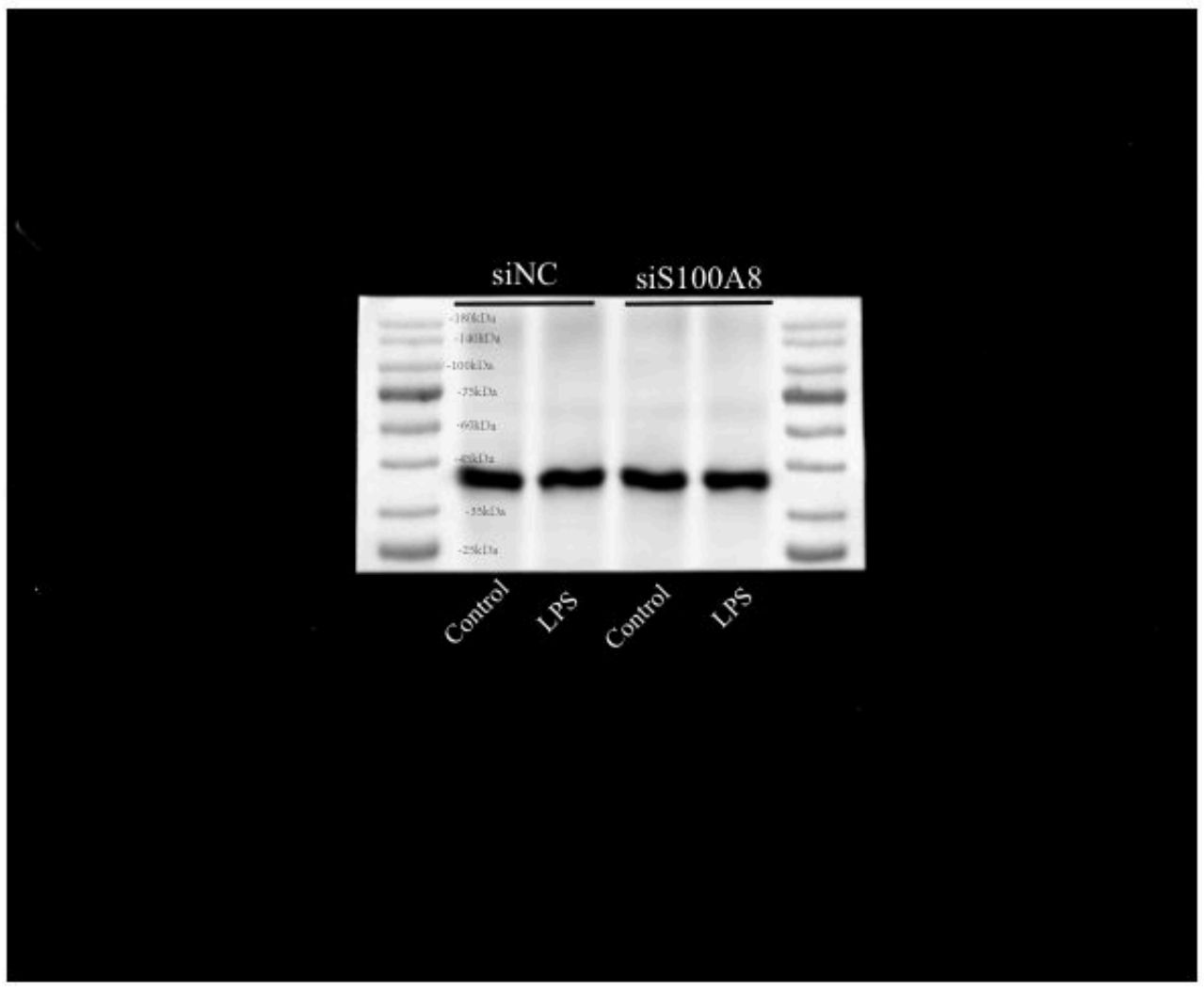

β-actin 42kDa

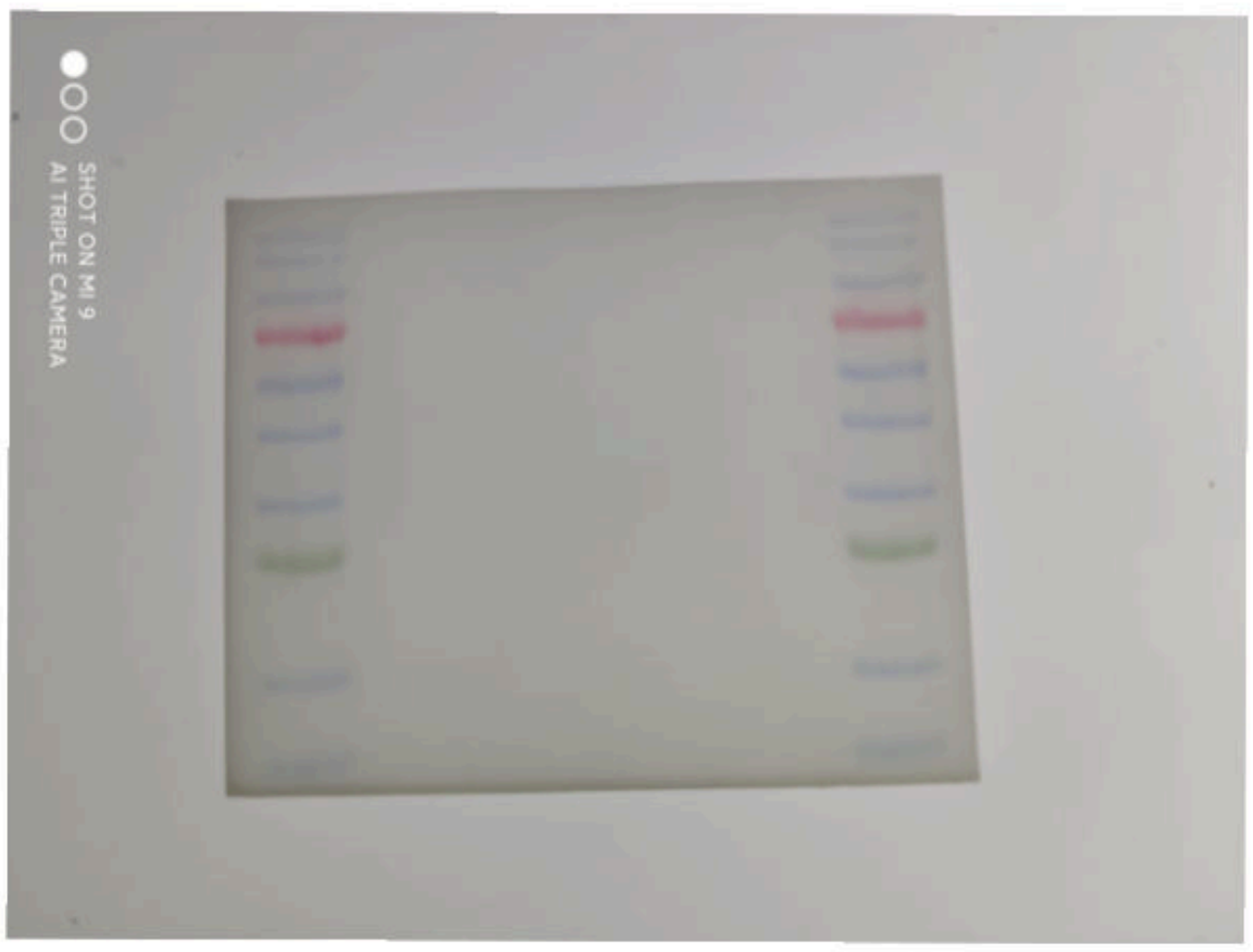

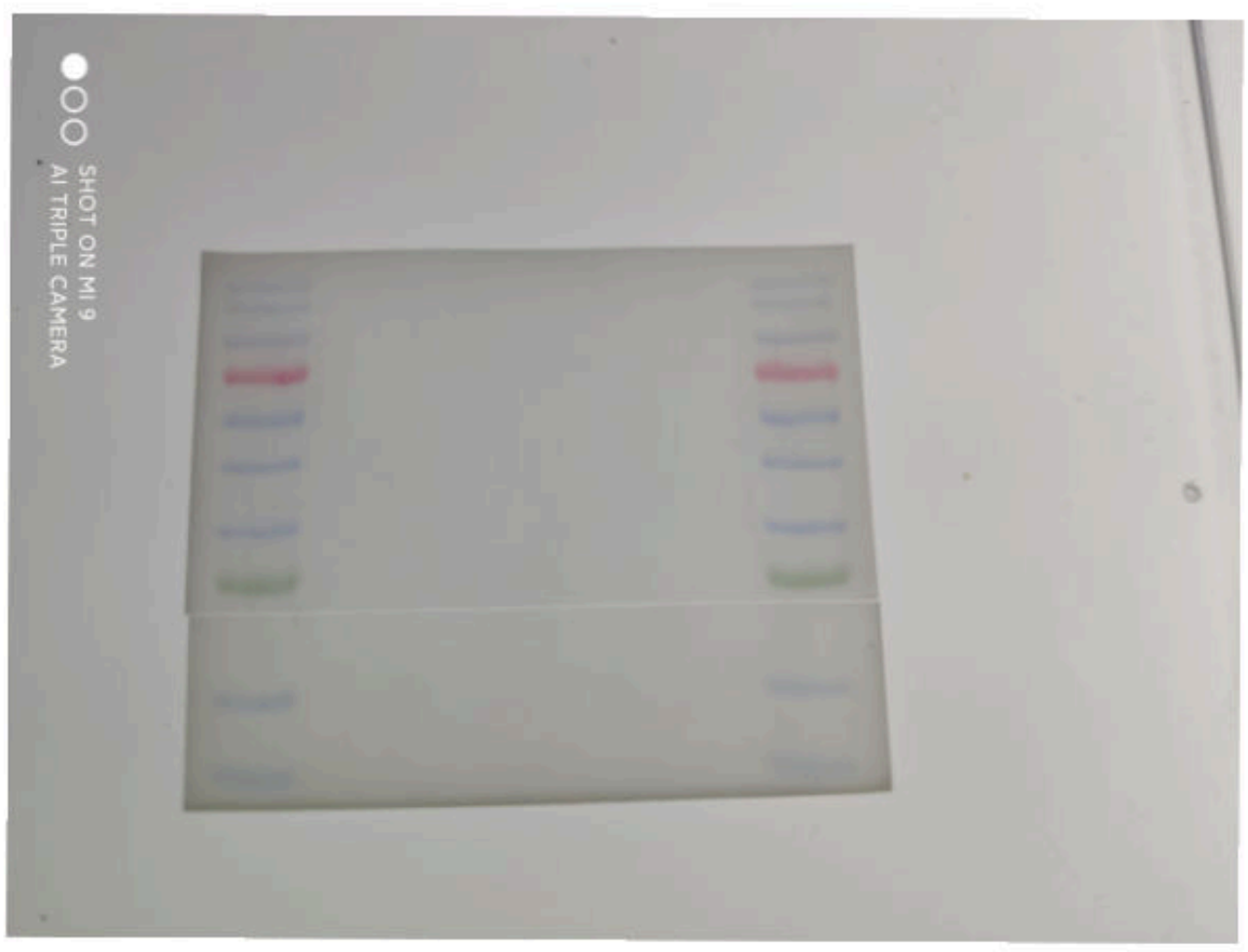

Source: New_validation_WB_source.pdf, original page 9. No brightness/contrast/gamma adjustment applied during compilation.
