# Supplementary material for: Multi-cohort transcriptomics and donor-aware single-cell analyses prioritize S100A8 within an emergency-myeloid, HLA-DR-low state in sepsis
Source: PLoS One. 2026 Oct 21;21(10):e0359578. doi: 10.1371/journal.pone.0359578 (PMC13686462; doi:10.1371/journal.pone.0359578)
Supplement: Supplementary file 10 [file pone.0359578.s017.zip › wgcna_final_rerun/soft_threshold_selection.pdf]

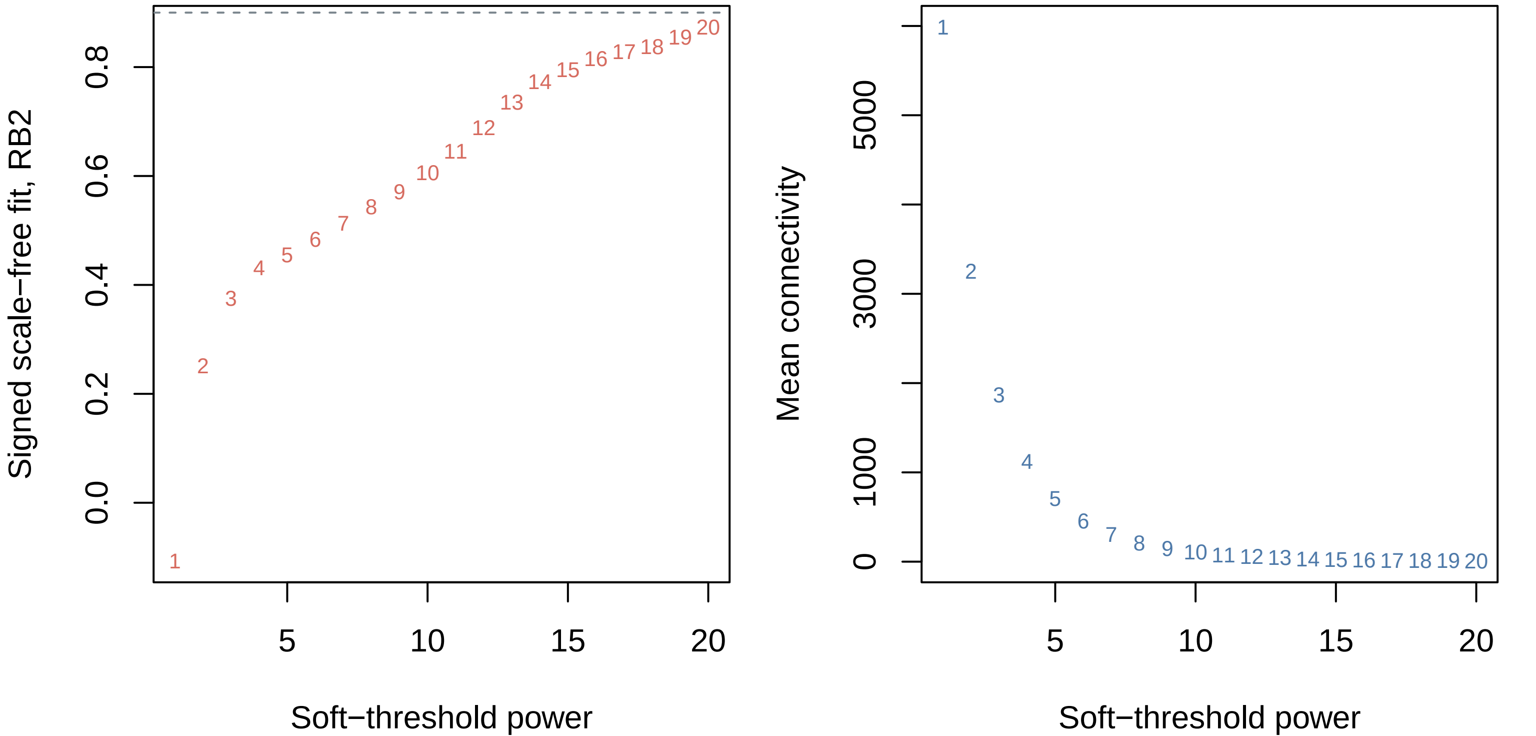
Signed scale−free fit, RB2
0.0
0.2
0.4
0.6
0.8
1
2
3
4
5
6
7
8
9
10
11
12
13
14
15
16
17
18
19
20
5
10
15
20
Soft−threshold power
Mean connectivity
0
1000
3000
5000
1
2
3
4
5
6
7
8
9
10
11
12
13
14
15
16
17
18
19
20
5
10
15
20
Soft−threshold power
