# Supplementary material for: Multi-cohort transcriptomics and donor-aware single-cell analyses prioritize S100A8 within an emergency-myeloid, HLA-DR-low state in sepsis
Source: PLoS One. 2026 Oct 21;21(10):e0359578. doi: 10.1371/journal.pone.0359578 (PMC13686462; doi:10.1371/journal.pone.0359578)
Supplement: Supplementary file 10 [file pone.0359578.s017.zip › wgcna_meanprobe_sensitivity/PCA_all_802.pdf]

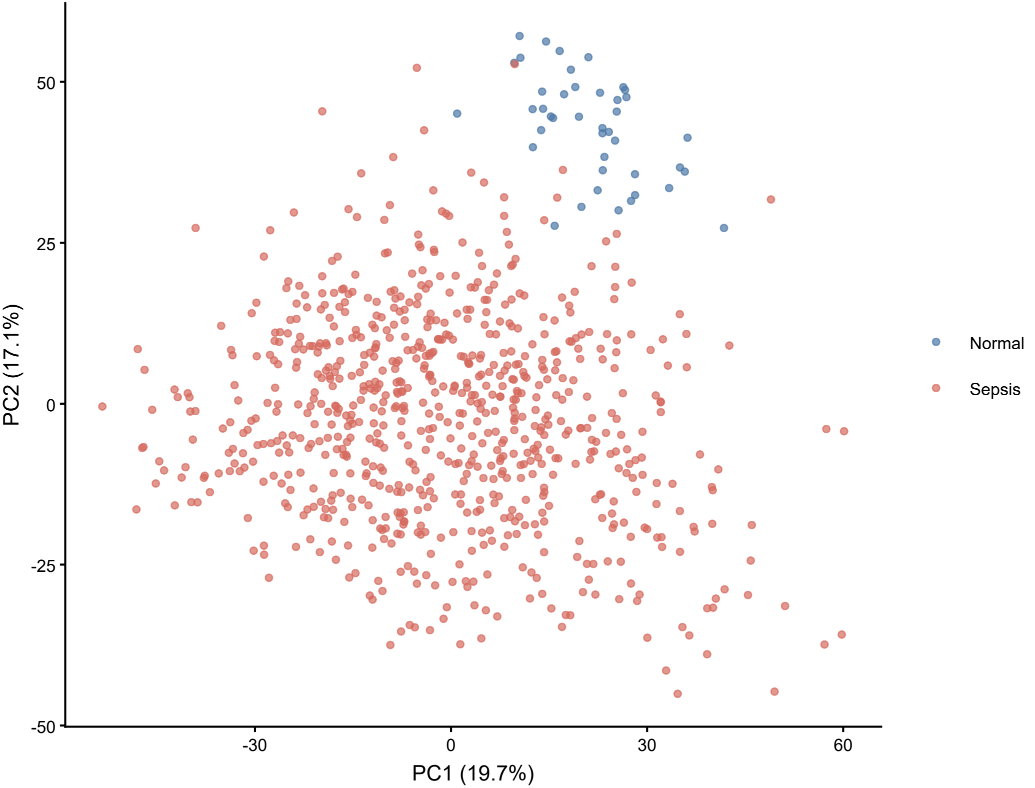

50
25
0
-25
-50
-30
0
30
60
PC2 (17.1%)
PC1 (19.7%)
Normal
Sepsis
