# Supplementary material for: Multi-cohort transcriptomics and donor-aware single-cell analyses prioritize S100A8 within an emergency-myeloid, HLA-DR-low state in sepsis
Source: PLoS One. 2026 Oct 21;21(10):e0359578. doi: 10.1371/journal.pone.0359578 (PMC13686462; doi:10.1371/journal.pone.0359578)

All 802 discovery samples retained

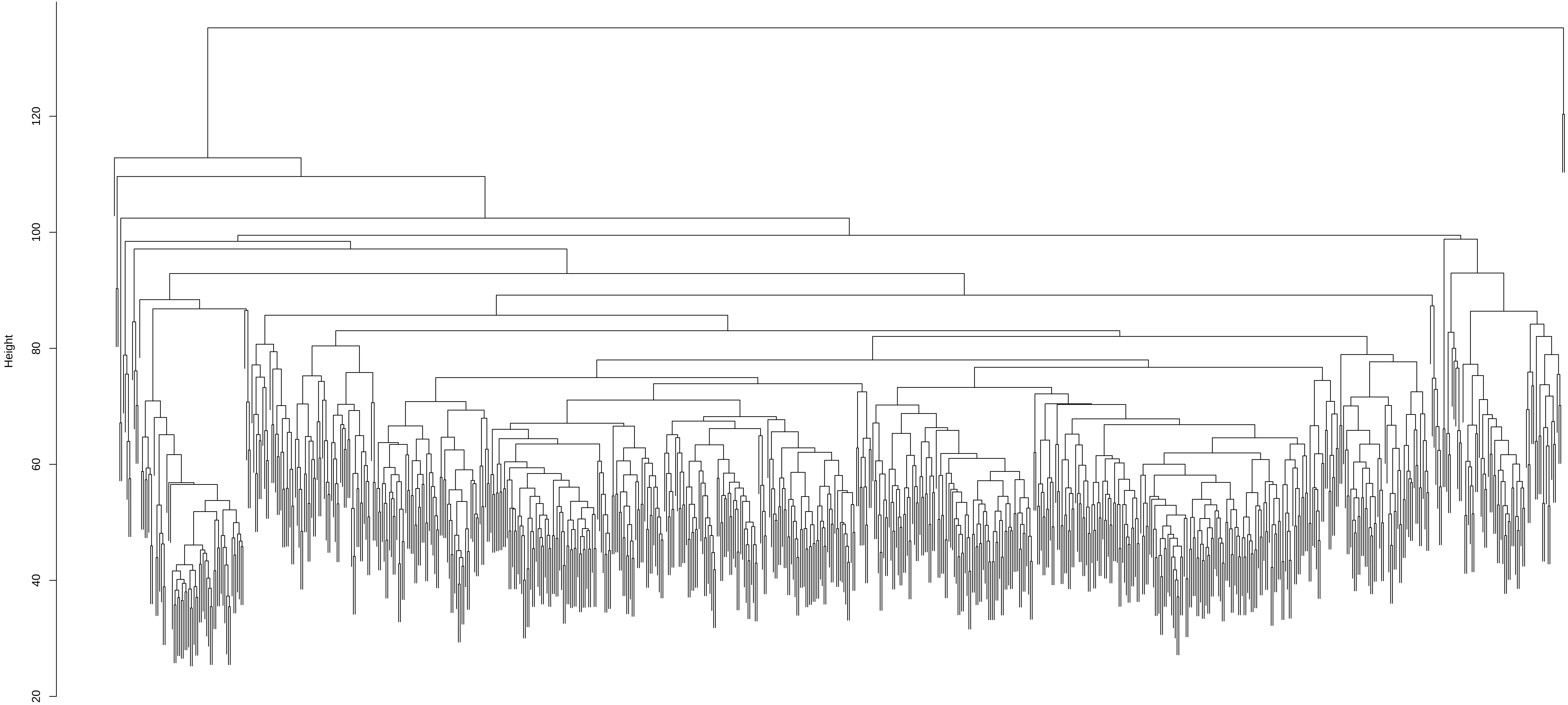

Supplement: Supplementary file 10 [file pone.0359578.s017.zip › wgcna_meanprobe_sensitivity/sample_clustering_all_802.pdf]
